# Efficient *N*-arylation of 4-chloroquinazolines en route to novel 4-anilinoquinazolines as potential anticancer agents

**DOI:** 10.3762/bjoc.17.206

**Published:** 2021-12-22

**Authors:** Rodolfo H V Nishimura, Thiago dos Santos, Valter E Murie, Luciana C Furtado, Leticia V Costa-Lotufo, Giuliano C Clososki

**Affiliations:** 1 Núcleo de Pesquisas em Produtos Naturais e Sintéticos, Departamento de Ciências BioMoleculares, Faculdade de Ciências Farmacêuticas de Ribeirão Preto, Universidade de São Paulo, Av. do Café s/n, Ribeirão Preto-SP 14040-903, Brazil; 2 Departamento de Química, Faculdade de Filosofia, Ciências e Letras de Ribeirão Preto-SP, Universidade de São Paulo, Av. dos Bandeirantes 3900, Ribeirão Preto-SP 14090-901, Brazil,; 3 Instituto de Ciências Biomédicas, Universidade de São Paulo, Av. Lineu Prestes 1524, 05508-900 São Paulo-SP, Brazil

**Keywords:** 4-anilinoquinazoline, anticancer agents, *N*-arylation, 4-chloroquinazoline, microwave irradiation

## Abstract

Microwave-mediated *N*-arylation of 4-chloroquinazolines in THF/H_2_O rapidly and efficiently afforded a library of novel 6-halo-2-phenyl-substituted 4-anilinoquinazolines. The methodology was compatible with numerous *ortho-*, *meta-*, and *para*-substituted *N*-methylanilines as well as substituted anilines and furnished the corresponding 4-anilinoquinazolines in good yields. Preliminary screening of the synthesized compounds against tumor cells (HCT-116 and T98G) showed promising antiproliferative properties.

## Introduction

**Figure 1:**
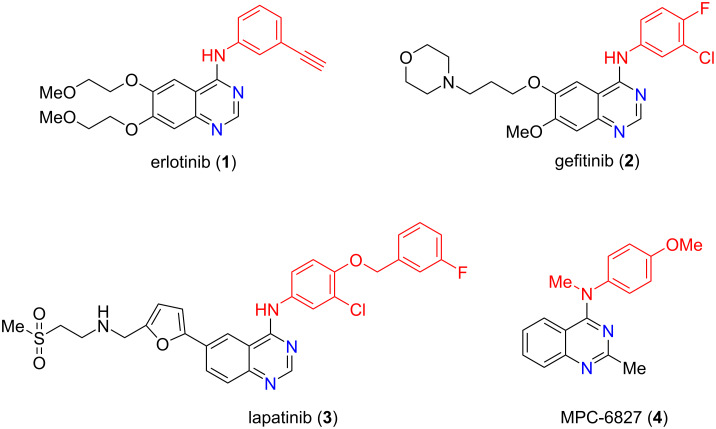
Some antitumor agents containing the 4-anilinoquinazoline moiety.

*N*-Heterocyclic compounds are commonly present in pharmaceuticals, bioactive natural products, agrochemicals, and synthetic drugs [1–2]. Quinazoline, a benzo-fused *N*-heterocyclic framework (benzo-1,3-diazine) with relevant biological activities, is recognized as a privileged scaffold in medicinal chemistry [3–4]. Among important quinazoline derivatives, 4-anilinoquinazolines have been widely investigated as antitumor agents because they can inhibit some receptor tyrosine kinases (RTKs) expressed by malignant tumors, including platelet-derived growth factor receptor beta (PDGFR-β), vessel epidermal growth factor receptor (VEGFR-2), and epidermal growth factor receptor (EGFR) [5–6]. In addition, these compounds may act as vascular disrupting agents and tubulin polymerization inhibitors, contributing to apoptosis [7]. Figure 1 highlights the structures of three EGFR inhibitors approved by the United States Food and Drug Administration (FDA) and one known tubulin inhibitor: erlotinib (**1**), gefitinib (**2**), lapatinib (**3**), and MPC-6827 – verubulin (**4**) [5,7].

Given that 4-anilinoquinazolines are potential antitumor agents, several synthetic methodologies have been developed to prepare these compounds. Amination using primary [6,8–13] or secondary [14–16] amines and 4-chloroquinazolines is among the most employed procedures.

**Scheme 1:**
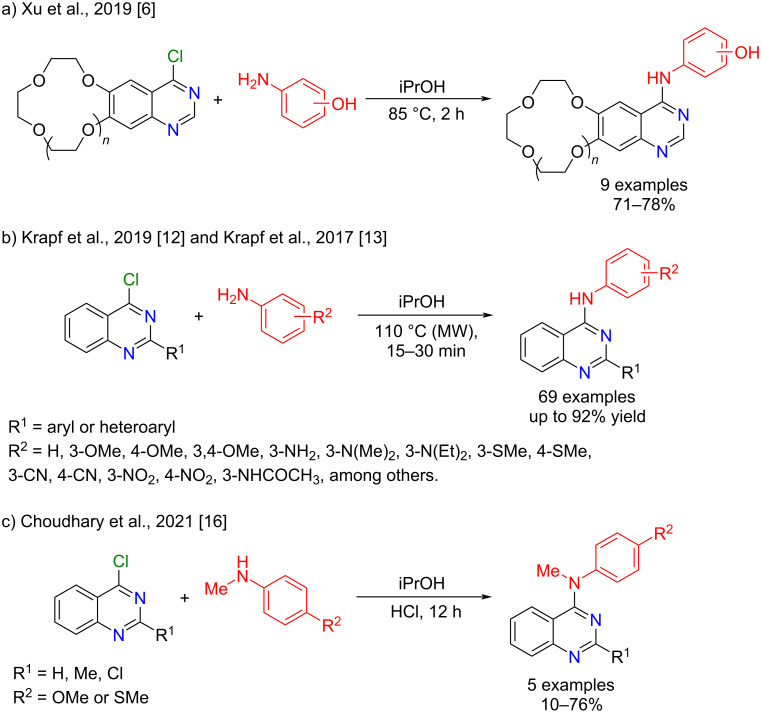
Examples of *N*-arylation reactions using 4-chloroquinazolines as substrates.

Electron-rich amines (e.g., primary aliphatic amines or hydroxy-substituted anilines) [6,9] readily react with 4-chloroquinazolines to give 4-aminoquinazolines in moderate to good yields under milder reaction conditions (Scheme 1a). On the other hand, long reaction times and low yields may be observed when electron-poor amines are applied in these reactions [17–18]. These limitations can be overcome by using microwave irradiation [12–13,18–20], which promotes fast and efficient anilination reactions when a wide range of anilines bearing both electron-donating and electron-withdrawing groups are employed as nucleophiles (Scheme 1b) [12–13]. Moreover, 4-anilinoquinazolines can be prepared from *N*-methylanilines under basic [14] or acidic [15–16] (Scheme 1c) conditions.

**Scheme 2:**
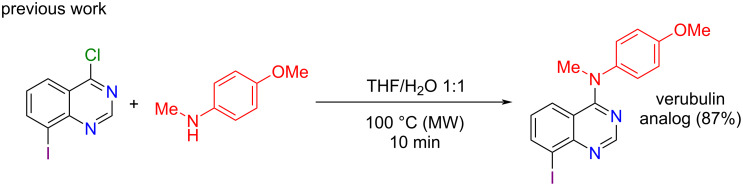
Synthesis of verubulin analog.

Due to our interest in the functionalization of aromatic and heterocyclic compounds of medicinal relevance [21–26], we recently reported the preparation of an iodo-substituted analog of the anticancer agent verubulin. Thus, after regioselective 4-chloroquinazoline metalation by an in situ trapping metalation strategy, reaction quenching with iodine allowed us to isolate 4-chloro-8-iodoquinazoline in 83% yield. Surprisingly, further reaction of 4-chloro-8-iodoquinazoline with 4-methoxy-*N*-methylaniline was not efficient under various reaction conditions and afforded the desired product in low yields even in basic medium (AcONa). To address this issue, we investigated a microwave-mediated base-free amination strategy involving a mixture of THF and water (1:1) as solvent system, which furnished the desired verubulin analog in 87% yield (Scheme 2) [27].

Inspired by this preliminary result, we decided to investigate this methodology to synthesize other bioactive derivatives, such as 6-halo-2-phenyl-substituted 4-anilinoquinazolines – the presence of an aryl group at C2 and bromo at C6 as substituents in quinazoline ring has been related to increased antiproliferative action of this class of compounds [28]. Therefore, we wish to report the preparation of a library of novel quinazoline-based antitumor candidates through reaction of 4-chloro-6-halo-2-phenylquinazolines with substituted anilines or *N*-methylanilines bearing electron-withdrawing or -donating groups at the *ortho*-, *meta-*, or *para-*positions.

## Results and Discussion

**Scheme 3:**
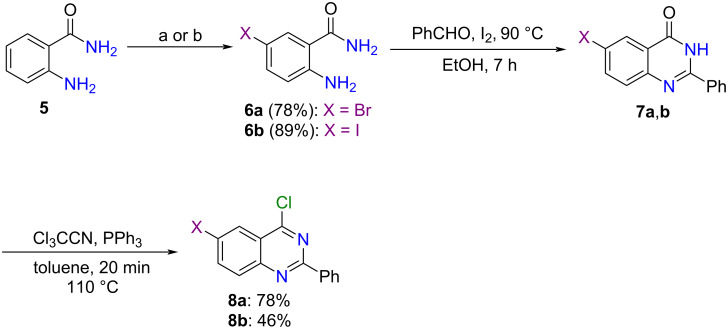
Synthesis of 4-chloro-6-halo-2-phenylquinazolines **8a** and **8b**. Conditions: a) NBS, CH_3_CN, 30 min, 25 °C; b) H_2_O, H_2_O_2_, I_2_, 24 h, 50 °C.

We started by synthesizing 6-bromo-4-chloro-2-phenylquinazoline (**8a**) and 4-chloro-6-iodo-2-phenylquinazoline (**8b**) as substrates for the *N*-arylation study. While anthranilamide (**5**) bromination with *N*-bromosuccinimide in acetonitrile at room temperature [29] furnished 2-amino-5-bromobenzamide (**6a**) in 78% yield, iodination of **5** with iodine in the presence of hydrogen peroxide in water [30] at 50 °C provided 2-amino-5-iodobenzamide (**6b**) in 89% yield. After that, the cyclocondensation [29] of the halogenated anthranilamides **6a**,**b** with benzaldehyde followed by dehydrogenation promoted by iodine gave the corresponding quinazolin-4(3*H*)-ones **7a**,**b**, which we used in the next step without purification. Finally, chlorination [31] of quinazolin-4(3*H*)-ones **7a**,**b** by employing a combination of Cl_3_CCN/PPh_3_ afforded 6-bromo-4-chloro-2-phenylquinazoline (**8a**) and 4-chloro-6-iodo-2-phenylquinazoline (**8b**) in 78 and 46% yields (over two steps), respectively (Scheme 3).

With the 4-chloroquinazolines **8a** and **8b** in hands, we investigated how they reacted with some *N*-methylanilines (**9a**–**k**) by using our established microwave-mediated protocol (Table 1). The reaction of **8a** or **8b** with *N*-methylanilines bearing a methoxy group at the *para-* (**9a**) or *meta-* (**9b**) position furnished the desired 4-anilinoquinazolines **10a**–**d** within 10 min; yields ranged from 63 to 90% (Table 1, entries 1–4). As expected, substituents at the *ortho-*position of the anilines affected the *N*-arylation reactions due to the steric hindrance exerted by these groups. In fact, reactions with 2-methoxy-substituted *N*-methylaniline **9c** were slower, but we obtained high conversions within 20 min under microwave heating, which allowed us to isolate the 4-anilinoquinazoline derivatives **10e** and **10f** in 87 and 84% yields, respectively (Table 1, entries 5 and 6). In contrast, no *N*-arylated products emerged when we used the *ortho*-methyl-substituted *N*-methylaniline **9d** as nucleophile, even when we conducted the reactions at 120 °C for 1 h (Table 1, entries 7 and 8). Subsequently, we employed the *meta*-methyl-substituted derivative **9e** as nucleophile under the established conditions, to obtain the desired anilinated derivatives **10i** and **10j** in 80 and 84% yields, respectively (Table 1, entries 9 and 10).

**Table 1:** 4-Anilinoquinazoline derivatives **10** obtained from *N*-arylation reactions involving 4-chloroquinazolines **8** and *N*-methylanilines **9**.

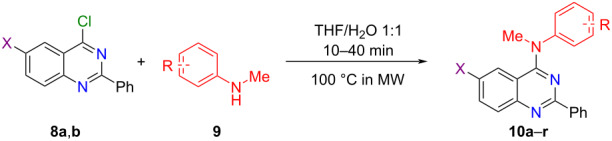				

Entry	X	R	Product	Yield (%)^a^

1	I	4-OMe (**9a**)	**10a**	86^b^
2	Br	4-OMe (**9a**)	**10b**	63^b^
3	I	3-OMe (**9b**)	**10c**	90^b^
4	Br	3-OMe (**9b**)	**10d**	84^b^
5	I	2-OMe (**9c**)	**10e**	87^c^
6	Br	2-OMe (**9c**)	**10f**	84^c^
7	I	2-Me (**9d**)	**10g**	no reaction^d,e^
8	Br	2-Me (**9d**)	**10h**	no reaction^d,e^
9	I	3-Me (**9e**)	**10i**	80^b^
10	Br	3-Me (**9e**)	**10j**	84^b^
11	I	3-Br (**9f**)	**10k**	72^b^
12	Br	3-Br (**9f**)	**10l**	73^b^
13	I	4-F (**9g**)	**10m**	84^f^
14	Br	4-F (**9g**)	**10n**	75^f^
15	I	3-F (**9h**)	**10o**	72^c^
16	Br	3-F (**9h**)	**10p**	70^b^
17	I	2-F (**9i**)	**10q**	no reaction^d,e^
18	Br	2-F (**9i**)	**10r**	no reaction^d,e^
19	I	4-CN (**9j**)	**10s**	no reaction^d,e^
20	Br	4-CN (**9j**)	**10t**	no reaction^d,e^
21	Br	4-NO_2_ (**9k**)	**10u**	no reaction^g^

^a^Isolated yield; ^b^reaction time = 10 min; ^c^reaction time = 20 min; ^d^reaction time = 1 h; ^e^temperature = 120 °C; ^f^reaction time = 40 min; ^g^reaction time = 2 h.

We also evaluated how the halogen substituents present in the anilines affected the *N*-arylation reactions. When we used 3-bromo-*N*-methylaniline (**9f**), we isolated derivatives **10k** and **10l** in 72 and 73% yields, respectively (Table 1, entries 11 and 12). Similarly, the reactions using 4-fluoro- or 3-fluoro-*N*-methylaniline (**9g** and **9h**, respectively) allowed us to isolate the expected derivatives **10m**–**p** in yields ranging from 70 to 84% (Table 1, entries 13–16). The electron-withdrawing effect of the fluoro substituent appeared to affect the reactivity of amine **9g** in those reactions drastically, so a longer reaction time (40 min) was necessary to obtain the desired products in good yield. Moreover, we did not observe any products when we used the 2-fluoro-substituted aniline **9i** even at higher temperature and for longer reaction time (Table 1, entries 17 and 18). Furthermore, no aminated products were observed when **8a** and **8b** were reacted with 4-cyano- or 4-nitro-*N*-methylaniline (Table 1, entries 19–21).

**Scheme 4:**
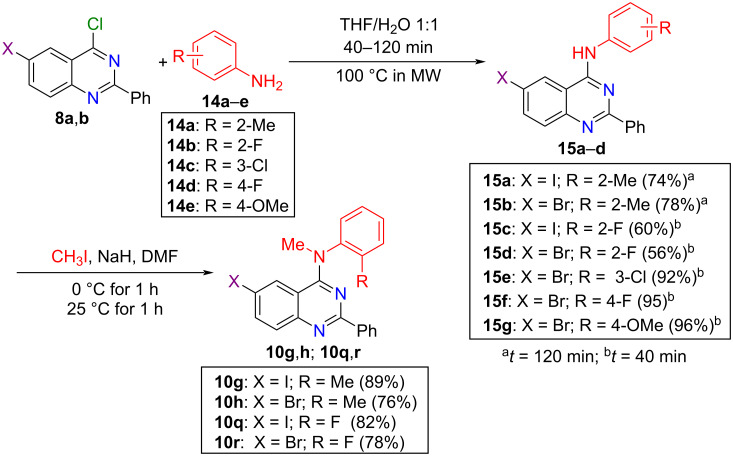
*N*-Arylation reactions using *ortho*-, *meta*-, and *para*-substituted primary anilines of type **14** followed by methylation of *ortho*-derivatives **15a**–**d**, to obtain the desired 4-anilinoquinazolines of type **10**.

Given our interest in preparing 4-anilinoquinazolines **10g**, **10h**, **10q**, and **10r**, we decided to investigate a two-step strategy starting by *N*-arylation using *o*-methyl- and *o*-fluoro-substituted primary anilines, followed by *N*-methylation [15]. The microwave-mediated reaction of 4-chloro-6-halo-2-phenylquinazolines **8a** or **8b** with *o*-toluidine (**14a**) in THF/H_2_O could be accomplished within 2 h and afforded the corresponding quinazoline derivatives **15a** and **15b** in 74% and 78% isolated yields, respectively. In addition, when we used 2-fluoroaniline (**14b**), we obtained derivatives **15c** and **15d** in 60 and 56% yields, respectively, after heating for 40 min (Scheme 4). Then, the derivatives **15a–d** were efficiently *N*-methylated with iodomethane in the presence of sodium hydride, to afford the corresponding methylated 4-anilinoquinazolines **10g**, **10h**, **10q**, and **10r** in yields ranging from 76 to 89% (Scheme 4). Moreover, to demonstrate the applicability of *meta*- and *para*-substituted primary anilines employing our *N*-arylation procedure, we evaluated reactions using 3-chloro-, 4-fluoro- and 4-methoxyaniline (**14c**–**e**, respectively) with 6-bromo-4-chloro-2-phenylquinazoline (**8a**), which provided the respective products **15e**–**g** in yields ranging from 92 to 96% (Scheme 4).

**Scheme 5:**
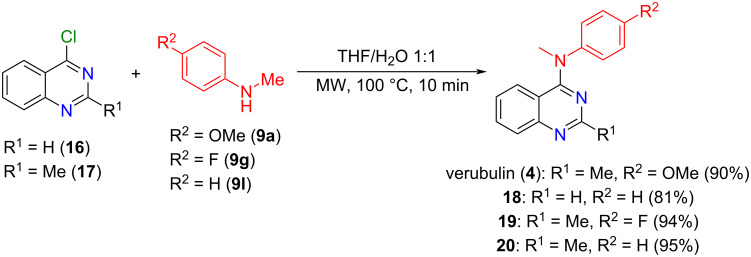
*N*-Arylation reactions using 4-chloroquinazoline (**16**) and 4-chloro-2-methylquinazoline (**17**) to achieve the desired 4-anilinoquinazolines (**4**,**18**–**20**).

Finally, to illustrate the importance and scope of the methodology, we used the commercially available 4-chloroquinazoline (**16**) and 4-chloro-2-methylquinazoline (**17**), which was prepared by chlorination of the corresponding quinazolinone (see Supporting Information File 1), in the *N*-arylation reaction. *N*-Arylation of **16** with *N*-methylaniline (**9l**) afforded *N*-methyl-*N*-phenylquinazolin-4-amine (**18**) in 81% yield (Scheme 5). Subsequently, the reaction of **17** with *N*-methylanilines **9a**, **9g**, and **9l** provided the desired 4-anilinoquinazolines **4**, **19**, and **20** in yields ranging from 90 to 95% (Scheme 5), among which we can highlight the anticancer agent verubulin (**4**).

The methodology reported herein allowed us to prepare numerous novel 4-anilinoquinazolines derivatives in good yield within short reaction times. To the best of our knowledge, only few examples of the use of *ortho*-substituted anilines for the anilination of 4-chloroquinazolines have been reported [6,18]. Thus, the established methodology proved an important alternative to synthesize these compounds from *ortho*-substituted anilines bearing both electron-withdrawing and -donating groups. Furthermore, compared to a previously reported protocol that uses AcONa as base and a THF/H_2_O 3:1 mixture as solvent [11,32], the protocol we developed here is base-free and requires a reduced amount of organic solvent, thereby being a more environmentally friendly methodology.

To evaluate the antiproliferative properties of the 4-anilinoquinazolines we obtained in this study, we screened the synthesized compounds against a set of tumor cell lines including HCT-116 (human colorectal carcinoma), MCF-7 (human breast adenocarcinoma), and T98G (human glioblastoma). Initially, we evaluated all compounds at 50 µM, and we considered active the compounds that inhibited cell proliferation by over 75% for each cell line. Compounds **10b**, **10c**, **10g**, **10k**, **10l**, **15a**, **15b**, and **15d** were active against T98G cells, and compounds **10b**, **15a**, and **15b** were also active against HCT-116 cells. However, none of the tested compounds was able to inhibit MCF-7 cell proliferation (Figure S1 in Supporting Information File 1).

Next, we determined the IC_50_ of the active compounds against both HCT-116 and T98G cells; we employed doxorubicin as positive control. We also investigated the potency of erlotinib hydrochloride, gefitinib, and verubulin against T98G cells, which were the most sensitive to the novel 4-anilinoquinazolines (Figure S2 in Supporting Information File 1). Compared to the reference drug, doxorubicin, derivative **10b** showed promising IC_50_ values (2.8 and 2.0 µM against HCT-116 and T98G cells, respectively). Additionally, the C2 phenyl ring in compound **10b** may be subjected to future replacements for optimization as less bulky groups at this position seem to be of great interest for gain in activity (e.g., 2-methyl group in verubulin). The EGFR inhibitor drugs bearing the 4-anilinoquinazoline moiety did not show potent cytotoxic activity against T98G cells (21.3 µM for erlotinib, and 37.8 µM for gefitinib). However, the tubulin polymerization inhibitor (verubulin), which contains 4-anilinoquinazoline in its chemical structure, was strongly cytotoxic to T98G cells (0.2 nM). In fact, the promising activity of verubulin in glioma is known, which has prompted phase 2 clinical trials in patients with recurrent glioblastoma, but the studies have been interrupted due to the observed adverse events [33]. Nonetheless, further studies aiming at optimizing the activity of compound **10b** and better understanding its mechanism of action and selectivity are underway in our research group.

**Table 2:** Evaluation of HCT-116 and T98G cell growth inhibition upon exposure to 4-anilinoquinazolines and compounds with the same scaffolds (erlotinib, gefitinib, and verubulin), and to positive control doxorubicin (MTT assay after treatment for 72 h).

HCT-116 cell line		

Compound	IC_50_ (µM)^a^	CI 95% (µM)^b^

**10b**	2.8	2.0–4.0
**15a**	26.2	20.7–32.9
**15b**	33.4	26.7–41.5
doxorubicin	0.1	0.1–0.1

T98G cell line		

Compound	IC_50_ (µM)^a^	CI 95% (µM)^b^

**10b**	2.0	1.1–3.8
**10c**	7.7	6.2–nd^c^
**10g**	33.6	26.1–nd^c^
**10k**	38.4	30.9–nd^c^
**10l**	>50	nd^c^
**15a**	28.6	21.6–nd^c^
**15b**	>50	42.5–67.9
**15d**	3.1	2.2–4.2
erlotinib	21.3	16.1–28.1
gefitinib	37.8	nd^c^
verubulin	0.2 (nM)	0.1–0.3 (nM)
doxorubicin	0.6	0.4–0.9

^a^IC_50_: half-maximal inhibitory concentration; ^b^CI 95%: confidence interval; ^c^nd: not determined.

## Conclusion

We have synthesized a library of novel 6-halo-2-phenyl-substituted 4-anilinoquinazolines through *N*-arylation of 4-chloro-substituted substrates with different anilines. The developed base-free protocol was compatible with several anilines bearing substituents at the *ortho-*, *meta-*, or *para-*positions, giving the corresponding *N*-arylated derivatives in yields of up to 96%. Moreover, it allowed fast and efficient reactions that required reduced amounts of organic solvents, so it is more sustainable than the protocols reported in the literature. Preliminary screening against selected tumor cells lines (HCT-116, MCF-7, and T98G) demonstrated the medicinal relevance of the 4-anilinoquinazolines synthesized here: many compounds (**10b**, **10c**, **10g**, **10k**, **10l**, **15a**, **15b**, and **15d**) proved active against at least one of the tested tumor cell lines. Compound **10b** afforded the most promising IC_50_ values (2.8 and 2.0 µM against HCT-116 and T98G cells, respectively). The scope of the developed methodology and its applicability toward the synthesis of other biologically active molecules are currently being investigated in our laboratories.

## Supporting Information

File 1 Experimental procedures, characterization data, copies of ^1^H and ^13^C spectra and additional information of antiproliferative assay.
