# Supplementary material for: Efficient N-arylation of 4-chloroquinazolines en route to novel 4-anilinoquinazolines as potential anticancer agents
Source: Beilstein J Org Chem. 2021 Dec 22;17:2968–75. doi: 10.3762/bjoc.17.206 (PMC8712970; doi:10.3762/bjoc.17.206)
Supplement: File 1 — Experimental procedures, characterization data, copies of 1H and 13C spectra and additional information of antiproliferative assay. [file Beilstein_J_Org_Chem-17-2968-s001.pdf]

BEILSTEIN JOURNAL OF ORGANIC CHEMISTRY

Supporting Information

for

Efficient *N*-arylation of 4-chloroquinazolines en route to novel 4-anilinoquinazolines as potential anticancer agents

Rodolfo H. V. Nishimura, Thiago dos Santos, Valter E. Murie, Luciana C. Furtado, Leticia V. Costa-Lotufo and Giuliano C. Clososki

Beilstein J. Org. Chem. **2021,** *17,* 2968–2975. doi:10.3762/bjoc.17.206

Experimental procedures, characterization data, copies of ^{1}H and ^{13}C spectra and additional information of antiproliferative assay

License and Terms: This is a supporting information file under the terms of the Creative Commons Attribution License (https://creativecommons.org/licenses/by/4.0). Please note that the reuse, redistribution and reproduction in particular requires that the author(s) and source are credited and that individual graphics may be subject to special legal provisions.
The license is subject to the *Beilstein Journal of Organic Chemistry* terms and conditions: (https://www.beilstein-journals.org/bjoc/terms)

Table of contents

1. Experimental S1

2. NMR spectra S23

3. Biological assays S57

4. References S58

1. Experimental

1.1. General information

Commercially available reagents were used without further purification. Flash chromatography was carried out by using silica gel (40–63 µm, 230–400 mesh, 60 Å) purchased from Aldrich. NMR spectra were recorded on a Bruker DPX-300, DRX-400, or DRX-500 spectrometer. $CDCl_3$, CD_3OD, or DMSO-d_6 were used as deuterated solvents. Chemical shifts are reported in ppm relative to the TMS signal (δ = 0,00 ppm) or the solvent residual signal. Data are reported as follows: chemical shifts (multiplicity, coupling constants (Hz), integration). The infrared (IR) spectra were recorded from 4000–600 cm^{-1} on an IR 400 spectrophotometer (PerkinElmer). Samples were measured neat in a zinc selenide crystal ATR and the absorption bands are described in wavenumbers (cm^{-1}). Gas chromatography–mass spectrometry (GC–MS) was performed on a Shimadzu GC (model 2010) coupled to a Shimadzu QP 2010 Ultra MS operated in the electron impact ionization mode (70 eV). The HRMS spectra were measured on a Bruker Daltonics (model microTOF QII – ESI-TOF Mass Spectrometer).

1.2. Synthesis of 4-chloro-6-halo-2-phenylquinazolines

2-Amino-5-bromobenzamide (6a) (CAS: 16313-66-9): Synthesized in a manner analogous to [1]. To a solution of 2-aminobenzamide (50.0 mmol, 6.8 g) in 120 mL of CH_3CN was added NBS (52.5 mmol, 9.3 g). This mixture was stirred at 25 °C for 30 min. After that, the reaction was quenched using ice-cold water and the resulting precipitate was filtered and purified by recrystallization from acetonitrile,

furnishing the compound **6a** in 78% yield as pale yellow solid (8.35 g, 38.8 mmol); ^{1}H NMR (300 MHz, CD_3OD, ppm) δ: 7.66 (d, *J* = 2.3 Hz, 1H), 7.26 (dd, *J* = 8.8, 2.3 Hz, 1H), 6.67 (d, *J* = 8.8 Hz, 1H), 4.90 (s, 4H).

6-Bromo-2-phenylquinazolin-4(3*H*)-one (7a) (CAS: 27398-50-1)**:** Synthesized in a manner analogous to [1]. In a round-bottomed flask, 2-amino-5-bromobenzamide (19.4 mmol, 4.2 g), benzaldehyde (23.3 mmol, 2.4 mL), I_2 (38.8 mmol, 9.9 g), and 150 mL of ethanol were added. This mixture was kept under stirring at 80 °C for 7 h. After that, the reaction was quenched with ice-cold concentrated aqueous $Na_2S_2O_3$ solution. The resulting precipitate was filtered and purified by recrystallization from ethanol. This product was used in the next step without further characterization due to its low solubility.

6-Bromo-4-chloro-2-phenylquinazoline (8a) (CAS: 412923-42-3): Synthesized in a manner analogous to [2]. In a round-bottomed flask, the previously obtained 6-bromo-2-phenylquinazolin-4(3*H*)-one (**7a**), PPh_3 (58.2 mmol, 15.3 g), Cl_3CCN (29.1 mmol, 2.9 mL), and 150 mL of toluene were added. This mixture was kept under stirring at 110 °C for 20 min. After this time, the reaction mixture was filtered using silica and EtOAc and concentrated under reduced pressure to afford the crude product. Then, compound **8a** was isolated in 78% yield as yellow solid (4.83 g, 15.1 mmol) by flash column chromatography using hexanes/ethyl acetate 4:1 as an eluent; ^{1}H NMR (500 MHz, $CDCl_3$, ppm) δ: 8.57-8.55 (m, 2H), 8.39 (d, *J* = 1.9 Hz, 1H), 7.98 (dd, *J* = 8.9, 2.0 Hz, 1H), 7.94 (d, *J* = 8.9 Hz, 1H), 7.53-7.52 (m, 3H); ^{13}C NMR (125 MHz, $CDCl_3$, ppm) δ: 161.4, 160.6, 150.8, 138.5, 136.5, 131.6, 130.8, 128.9 (2C), 128.9 (2C), 128.2, 123.6, 122.1.

2-Amino-5-iodobenzamide (6b) (CAS: 32658-67-6): Synthesized in a manner analogous to [3]. To a suspension containing 2-aminobenzamide (30.0 mmol, 4.1 g), I_2 (30.0 mmol, 7.6 g), and 150 mL of H_2O was added hydrogen peroxide (30 wt %, 60.0 mmol, 6.8 mL). This mixture was stirred at 50 °C for 24 h. After that, the reaction was quenched using a concentrated aqueous $Na_2S_2O_3$ solution and the mixture was extracted with EtOAc (3 ×). The organic layers were dried with anhydrous $MgSO_4$ and concentrated under reduced pressure. After recrystallization from acetonitrile, compound **6b** was obtained in 89% yield as a brown solid (6.99 g, 26.7 mmol); ^{1}H NMR (500 MHz, DMSO-d_6, ppm) δ: 7.81 (d, J = 2.0 Hz, 1H), 7.37 (dd, J = 8.7, 2.0 Hz, 1H), 6.54 (d, J = 8.7 Hz, 1H), 3.36 (s, 4H); ^{13}C NMR (125 MHz, DMSO-d_6, ppm) δ: 169.9, 149.7, 139.8, 136.5, 118.9, 116.1, 74.4.

6-Iodo-2-phenylquinazolin-4(3*H*)-one (7b) (CAS: 82326-76-9)**:** Synthesized in a manner analogous to [1]. In a round-bottomed flask, 2-amino-5-iodobenzamide (14.7 mmol, 3.9 g), benzaldehyde (17.6 mmol, 1.8 mL), I_2 (29.4 mmol, 7.5 g), and 120 mL of ethanol were added. This mixture was kept under stirring at 80 °C for 7 h. After that, the reaction was quenched with ice-cold concentrated aqueous $Na_2S_2O_3$ solution. The resulting precipitate was filtered and purified by recrystallization from ethanol. This product was used in the next step without previous characterization due to its low solubility.

4-Chloro-6-iodo-2-phenylquinazoline (8b) (CAS: 257624-25-2): Synthesized in a manner analogous to [2]. In a round-bottomed flask, the previously obtained

6-iodo-2-phenylquinazolin-4(3*H*)-one (**7b**), PPh_3 (44.1 mmol, 11.5 g), Cl_3CCN (22.0 mmol, 2.2 mL), and 120 mL of toluene were added. This mixture was kept under stirring at 110 °C for 20 min. After this time, the reaction mixture was filtered using silica and EtOAc and concentrated under reduced pressure to afford the crude product. Then, compound **8b** was isolated in 46% yield as yellow solid (2.47 g, 6.74 mmol) by flash column chromatography using hexanes/ethyl acetate 19:1 as eluent; ^{1}H NMR (500 MHz, $CDCl_3$, ppm) δ: 8.62 (d, *J* = 1.7 Hz, 1H), 8.57 – 8.55 (m, 2H), 8.15 (dd, *J* = 8.8, 1.9 Hz, 1H), 7.80 (d, *J* = 8.8 Hz, 1H), 7.54 – 7.52 (m, 3H); ^{13}C NMR (125 MHz, $CDCl_3$, ppm) δ: 161.1, 160.6, 151.1, 143.8, 136.5, 134.8, 131.6, 130.6, 129.0 (2C), 128.9 (2C), 124.0, 93.4;

1.3. Synthesis of 6-halo-2-phenyl-substituted 4-anilinoquinazolines

Typical procedure 1 (TP1) – *N*-arylation of 4-chloro-6-halo-2-phenylquinazolines in microwave reactor.

In a microwave vial, the given aniline (1.05 equiv), 4-chloro-6-halo-2-phenylquinazoline (1.0 equiv), and a mixture of THF/H_2O 1:1 (6 $mL \cdot mmol^{-1}$ of quinazoline) were added. The reaction mixture was stirred further for the indicated times at 100 °C in a microwave reactor after which the reaction was quenched with saturated aqueous $NaHCO_3$ solution. The aqueous phase was extracted with AcOEt (3 ×), the organic layers were dried with $MgSO_4$, and concentrated under reduced pressure. After that, flash column chromatography using suited hexanes/ethyl acetate mixtures afforded the isolated products.

Typical procedure 2 (TP2) – *N*-methylation of 4-anilinoquinazolines.

In a round-bottomed flask, the given 4-anilinoquinazoline (1.0 equiv) was dissolved in DMF (15 mL·mmol^{-1}), subsequently, NaH (2.0 equiv) and CH_3I (24 equiv) were added at 0 °C. The reaction mixture was stirred at 0 °C for 1 h and at 25 °C for an additional 1 h, after which the reaction was quenched with concentrated aqueous NaCl solution. The aqueous phase was extracted with AcOEt (3 ×), the organic layers were dried with $MgSO_4$, and concentrated under reduced pressure. After that, flash column chromatography using suited hexanes/ethyl acetate mixtures afforded the isolated products.

6-Iodo-*N*-(4-methoxyphenyl)-*N*-methyl-2-phenylquinazolin-4-amine (10a): According to TP1, the following amount of each reagent was used: 4-chloro-6-iodo-2-phenylquinazoline (0.50 mmol, 183 mg), 4-methoxy-*N*-methylaniline (0.53 mmol, 72 mg). Reaction time: 10 min; mp (ºC): 141 – 143 °C; Appearance: Yellow solid; Yield: 202 mg (0.43 mmol, 86%); eluent: Hexanes/Ethyl acetate (9:1); ^{1}H NMR (400 MHz, $CDCl_3$, ppm) δ 8.61 – 8.58 (m, 2H), 7.76 (dd, *J* = 8.8, 1.8 Hz, 1H), 7.59 (d, *J* = 8.8 Hz, 1H), 7.53 – 7.48 (m, 3H), 7.23 (d, *J* = 1.8 Hz, 1H), 7.14 (d, *J* = 9.0 Hz, 2H), 6.97 (d, *J* = 9.0 Hz, 2H), 3.87 (s, 3H), 3.71 (s, 3H); ^{13}C NMR (100 MHz, $CDCl_3$, ppm) δ 160.3, 160.1, 158.9, 151.9, 140.7, 140.3, 138.6, 135.9, 130.6, 130.5, 128.6 (2C), 128.5 (2C), 127.6 (2C), 117.0, 115.6 (2C), 88.4, 55.9, 42.7; IR (ATR, cm^{-1}): 3064, 2926, 1484, 1371, 1244, 1097, 1033, 827, 706, 690; GC-MS (EI, 70 eV) *m/z* (%): 467 (100), 452 (7), 204 (25), 136 (55), 121 (43); HRMS (ESI/Q-TOF) *m/z*, calculated for $C_{22}H_{19}IN_3O^+$ $[M+H]^+$: 468.0567; found 468.0567.

6-Bromo-*N*-(4-methoxyphenyl)-*N*-methyl-2-phenylquinazolin-4-amine (10b): According to TP1, the following amount of each reagent was used: 6-bromo-4-chloro-2-phenylquinazoline (0.24 mmol, 77 mg), 4-methoxy-*N*-methylaniline (0.26 mmol, 35 mg). Reaction time: 10 min; mp (ºC): 151 – 153 °C; Appearance: Pale yellow solid; Yield: 64 mg (0.15 mmol, 63%); eluent: Hexanes/Ethyl acetate (19:1); ^{1}H NMR (400 MHz, $CDCl_3$, ppm) δ 8.61 – 8.59 (m, 2H), 7.74 (d, *J* = 8.9 Hz, 1H), 7.61 (dd, *J* = 8.9, 2.2 Hz, 1H), 7.53 – 7.48 (m, 3H), 7.15 (d, *J* = 9.0 Hz, 2H), 7.07 (d, *J* = 2.0 Hz, 1H), 6.96 (d, *J* = 9.0 Hz, 2H), 3.86 (s, 3H), 3.71 (s, 3H); ^{13}C NMR (100 MHz, $CDCl_3$, ppm) δ 160.7, 160.0, 158.7, 151.5, 140.8, 138.6, 135.1, 130.5, 130.5, 129.1, 128.5 (3C), 127.6 (2C), 117.3, 116.5, 115.6 (3C), 55.8, 42.9; IR (ATR, cm^{-1}): 3066, 2931, 1487, 1372, 1245, 1033, 837, 708; GC-MS (EI, 70 eV) *m/z* (%): 421 (85), 419 (80), 340 (10), 285 (29), 136 (100), 121 (75), 77 (80); HRMS (ESI/Q-TOF) *m/z*, calculated for $C_{22}H_{19}BrN_3O^+$ $[M+H]^+$: 420.0706; found 420.0707.

6-Iodo-*N*-(3-methoxyphenyl)-*N*-methyl-2-phenylquinazolin-4-amine (10c): According to TP1, the following amount of each reagent was used: 4-chloro-6-iodo-2-phenylquinazoline (0.20 mmol, 73 mg), 3-methoxy-*N*-methylaniline (0.21 mmol, 29 mg). Reaction time: 10 min; mp (ºC): 115 – 117 °C; Appearance: Yellow solid; Yield: 84 mg (0.18 mmol, 90%); eluent: Hexanes/Ethyl acetate (9:1); ^{1}H NMR (400 MHz, $CDCl_3$, ppm) δ 8.61 – 8.59 (m, 2H), 7.79 (dd, *J* = 8.8, 1.9 Hz, 1H), 7.62 (d, *J* = 8.8 Hz, 1H), 7.54 – 7.48 (m, 3H), 7.36 (d, *J* = 2.0 Hz, 1H), 7.33 (t, *J* = 8.1 Hz, 1H), 6.91 (ddd, *J* = 8.3, 2.4, 0.7 Hz, 1H), 6.80 (ddd, *J* = 7.9, 1.9, 0.7 Hz, 1H), 6.75 (t, *J* = 2.2 Hz, 1H), 3.79 (s, 3H), 3.75 (s, 3H); ^{13}C NMR (100 MHz, $CDCl_3$, ppm) δ 161.2, 160.4, 160.1, 151.8, 149.0, 140.6, 138.5, 135.8, 131.0, 130.6 (2C), 128.6 (2C), 128.5 (2C), 118.5, 117.2, 112.7, 112.2, 88.6, 55.8,

42.4; IR (ATR, cm^{-1}): 3044, 2934, 1526, 1487, 1378, 1220, 1048, 823, 698; GC-MS (EI, 70 eV) *m/z* (%): 467 (100), 340 (21), 233 (18), 205 (19), 136 (69), 77 (18); HRMS (ESI/Q-TOF) *m/z*, calculated for $C_{22}H_{19}IN_3O^+$ $[M+H]^+$: 468.0567; found 468.0562.

6-Bromo-*N*-(3-methoxyphenyl)-*N*-methyl-2-phenylquinazolin-4-amine (10d): According to TP1, the following amount of each reagent was used: 6-bromo-4-chloro-2-phenylquinazoline (0.20 mmol, 64 mg), 3-methoxy-*N*-methylaniline (0.21 mmol, 29 mg). Reaction time: 10 min; mp (ºC): 131 – 133 °C; Appearance: Yellow solid; Yield: 71 mg (0.17 mmol, 84%); eluent: Hexanes/Ethyl acetate (19:1); ^1H NMR (400 MHz, $CDCl_3$, ppm) δ 8.62 – 8.59 (m, 2H), 7.76 (d, *J* = 8.9 Hz, 1H), 7.63 (dd, *J* = 8.9, 2.2 Hz, 1H), 7.54 – 7.49 (m, 3H), 7.33 (t, *J* = 8.1 Hz, 1H), 7.17 (d, *J* = 2.1 Hz, 1H), 6.89 (ddd, *J* = 8.4, 2.4, 0.7 Hz, 1H), 6.80 (ddd, *J* = 7.9, 2.0, 0.8 Hz, 1H), 6.76 (t, *J* = 2.2 Hz, 1H), 3.78 (s, 3H), 3.75 (s, 3H); ^{13}C NMR (100 MHz, $CDCl_3$, ppm) δ 161.2, 160.8, 160.1, 151.5, 149.1, 138.5, 135.3, 131.0, 130.6, 130.5, 129.0, 128.5 (3C), 118.5, 117.5, 116.7, 112.6, 100.1, 112.1, 55.7, 42.5; IR (ATR, cm^{-1}): 2923, 1553, 1485, 1390, 1227, 1097, 948, 828, 705; GC-MS (EI, 70 eV) *m/z* (%): 421 (63), 419 (65), 340 (14), 286 (22), 210 (15), 136 (100), 77 (47); HRMS (ESI/Q-TOF) *m/z*, calculated for $C_{22}H_{19}BrN_3O^+$ $[M+H]^+$: 420.0706; found 420.0712.

6-Iodo-*N*-(2-methoxyphenyl)-*N*-methyl-2-phenylquinazolin-4-amine (10e): According to TP1, the following amount of each reagent was used: 4-chloro-6-iodo-2-phenylquinazoline (0.32 mmol, 117 mg), 2-methoxy-*N*-methylaniline (0.33 mmol, 45 mg). Reaction time: 20 min; mp (ºC): 180 – 182 °C; Appearance: Yellow solid; Yield: 130 mg (0.28 mmol, 87%); eluent: Hexanes/Ethyl acetate (19:1); ^1H NMR (400 MHz, $CDCl_3$, ppm) δ 8.63 – 8.60 (m, 2H), 7.75 (dd, *J* = 8.8, 1.9 Hz,

1H), 7.60 (d, *J* = 8.8 Hz, 1H), 7.53 – 7.48 (m, 3H), 7.41 (ddd, *J* = 8.3, 7.6, 1.7 Hz, 1H), 7.24 (d, *J* = 1.9 Hz, 1H), 7.21 (dd, *J* = 8.1, 1.7 Hz, 1H), 7.06 – 7.02 (m, 2H), 3.68 (s, 3H), 3.67 (s, 3H); ^{13}C NMR (100 MHz, $CDCl_3$, ppm) δ 160.5, 160.0, 154.9, 151.5, 140.2, 138.8, 135.9, 134.7, 130.5, 130.4, 129.2, 128.6 (2C), 128.5 (3C), 121.9, 117.2, 112.9, 88.1, 55.8, 41.1; IR (ATR, cm^{-1}): 3064, 2976, 1484, 1371, 1232, 1095, 1019, 839, 750, 703; GC-MS (EI, 70 eV) *m/z* (%): 467 (6), 436 (100), 309 (17), 204 (11), 136 (10), 77 (14); HRMS (ESI/Q-TOF) *m/z*, calculated for $C_{22}H_{19}IN_3O^+$ $[M+H]^+$: 468.0567; found 468.0568.

6-Bromo-*N*-(2-methoxyphenyl)-*N*-methyl-2-phenylquinazolin-4-amine (10f): According to TP1, the following amount of each reagent was used: 6-bromo-4-chloro-2-phenylquinazoline (0.34 mmol, 109 mg), 2-methoxy-*N*-methylaniline (0.35 mmol, 48 mg). Reaction time: 20 min; mp (ºC): 193 – 195 °C; Appearance: Yellow solid; Yield: 120 mg (0.29 mmol, 84%); eluent: Hexanes/Ethyl acetate (19:1); ^{1}H NMR (400 MHz, $CDCl_3$, ppm) δ 8.63 – 8.61 (m, 2H), 7.74 (d, *J* = 8.9 Hz, 1H), 7.60 (dd, *J* = 8.9, 2.2 Hz, 1H), 7.54 – 7.48 (m, 3H), 7.40 (ddd, *J* = 8.3, 7.6, 1.7 Hz, 1H), 7.21 (dd, *J* = 8.1, 1.7 Hz, 1H), 7.06 – 7.02 (m, 3H), 3.68 (s, 3H), 3.68 (s, 3H); ^{13}C NMR (100 MHz, $CDCl_3$, ppm) δ 160.9, 160.0, 154.9, 151.2, 138.8, 135.9, 135.0, 130.4, 130.4, 129.2, 128.6 (2C), 128.5 (3C), 127.9, 121.9, 117.1, 116.7, 112.9, 55.8, 41.2; IR (ATR, cm^{-1}): 3059, 2941, 1530, 1486, 1252, 1025, 873, 743, 703; GC-MS (EI, 70 eV) *m/z* (%): 421 (4), 419 (4), 390 (100), 388 (100), 285 (10), 194 (10), 136 (16), 77 (44); HRMS (ESI/Q-TOF) *m/z*, calculated for $C_{22}H_{19}BrN_3O^+$ $[M+H]^+$: 420.0706; found 420.0704.

6-Iodo-*N*-methyl-2-phenyl-*N*-(3-tolyl)quinazolin-4-amine (10i): According to TP1, the following amount of each reagent was used: 4-chloro-6-iodo-2-phenylquinazoline (0.25 mmol, 92 mg), *N*,3-dimethylaniline (0.26 mmol, 32 mg).

Reaction time: 10 min; mp (ºC): 168 – 170 °C; Appearance: White solid; Yield: 90 mg (0.20 mmol, 80%); eluent: Hexanes/Ethyl acetate (19:1); ^{1}H NMR (400 MHz, $CDCl_3$, ppm) δ 8.61 – 8.59 (m, 2H), 7.77 (dd, *J* = 8.8, 1.9 Hz, 1H), 7.62 (d, *J* = 8.8 Hz, 1H), 7.54 – 7.48 (m, 3H), 7.33 (t, *J* = 7.7 Hz, 1H), 7.27 (d, *J* = 1.9 Hz, 1H), 7.19 (d, *J* = 7.6 Hz, 1H), 7.04 (s, 1H), 7.03 – 6.99 (m, 1H), 3.75 (s, 3H), 2.37 (s, 3H); ^{13}C NMR (100 MHz, $CDCl_3$, ppm) δ 160.3, 160.1, 151.8, 147.7, 140.5, 140.5, 138.6, 136.0, 130.5, 130.1, 128.6 (2C), 128.5 (3C), 128.0, 126.9, 123.3, 117.1, 88.3, 42.5, 21.5; IR (ATR, cm^{-1}): 3068, 2915, 1551, 1483, 1389, 1306, 1093, 942, 832, 703; GC-MS (EI, 70 eV) *m/z* (%): 451 (100), 324 (26), 205 (25), 120 (94), 101 (25), 77 (21); HRMS (ESI/Q-TOF) *m/z*, calculated for $C_{22}H_{19}IN_3^+$ $[M+H]^+$: 452.0618; found 452.0617.

6-Bromo-*N*-methyl-2-phenyl-*N*-(3-tolyl)quinazolin-4-amine (10j): According to TP1, the following amount of each reagent was used: 6-bromo-4-chloro-2-phenylquinazoline (0.25 mmol, 80 mg), *N*,3-dimethylaniline (0.26 mmol, 32 mg). Reaction time: 10 min; mp (ºC): 142 – 144 °C; Appearance: White solid; Yield: 85 mg (0.21 mmol, 84%); eluent: Hexanes/Ethyl acetate (19:1); ^{1}H NMR (400 MHz, $CDCl_3$, ppm) δ 8.62 – 8.59 (m, 2H), 7.75 (d, *J* = 8.9 Hz, 1H), 7.61 (dd, *J* = 8.9, 2.2 Hz, 1H), 7.54 – 7.48 (m, 3H), 7.31 (t, *J* = 7.7 Hz, 1H), 7.17 (d, *J* = 7.6 Hz, 1H), 7.08 (d, *J* = 2.1 Hz, 1H), 7.04 (s, 1H), 7.01 (d, *J* = 7.5 Hz, 1H), 3.74 (s, 3H), 2.36 (s, 3H); ^{13}C NMR (100 MHz, $CDCl_3$, ppm) δ 160.7, 160.1, 151.5, 147.8, 140.5, 138.6, 135.2, 130.5, 130.5, 130.1, 129.1, 128.5 (2C), 128.5 (2C), 127.9, 126.8, 123.3, 117.3, 116.6, 42.6, 21.5; IR (ATR, cm^{-1}): 2917, 1553, 1484, 1390, 1306, 1094, 943, 834, 703; GC-MS (EI, 70 eV) *m/z* (%): 405 (40), 403 (38), 286 (16), 284 (16), 205 (12), 120 (100), 77 (51); HRMS (ESI/Q-TOF) *m/z*, calculated for $C_{22}H_{19}BrN_3^+$ $[M+H]^+$: 404.0757; found 404.0757.

***N*-(3-Bromophenyl)-6-iodo-*N*-methyl-2-phenylquinazolin-4-amine (10k)**: According to TP1, the following amount of each reagent was used: 4-chloro-6-iodo-2-phenylquinazoline (0.31 mmol, 114 mg), 3-bromo-*N*-methylaniline (0.32 mmol, 59 mg). Reaction time: 10 min; mp (ºC): 164 – 166 °C; Appearance: White solid; Yield: 115 mg (0.22 mmol, 72%); eluent: Hexanes/Ethyl acetate (19:1); ^{1}H NMR (400 MHz, $CDCl_3$, ppm) δ 8.60 – 8.58 (m, 2H), 7.82 (dd, *J* = 8.8, 1.9 Hz, 1H), 7.65 (d, *J* = 8.8 Hz, 1H), 7.54 – 7.49 (m, 3H), 7.47 (ddd, *J* = 8.0, 1.8, 0.9 Hz, 1H), 7.40 (t, *J* = 2.0 Hz, 1H), 7.36 (d, *J* = 1.9 Hz, 1H), 7.27 (t, *J* = 8.0 Hz, 1H), 7.08 (ddd, *J* = 8.0, 2.1, 0.9 Hz, 1H), 3.74 (s, 3H); ^{13}C NMR (100 MHz, $CDCl_3$, ppm) δ 160.4, 160.1, 151.8, 149.4, 140.9, 138.2, 135.5, 131.3, 130.7 (2C), 129.8, 128.9, 128.6 (2C), 128.6 (2C), 124.5, 123.5, 117.0, 89.0, 42.4; IR (ATR, cm^{-1}): 3041, 2921, 1526, 1486, 1375, 1314, 1010, 825, 764, 700; GC-MS (EI, 70 eV) *m/z* (%): 517 (100), 515 (100), 390 (26), 388 (27), 332 (81), 217 (46), 205 (76), 154 (50), 101 (70), 77 (55); HRMS (ESI/Q-TOF) *m/z*, calculated for $C_{21}H_{16}BrIN_3^+$ $[M+H]^+$: 515.9567; found 515.9566.

6-Bromo-*N*-(3-bromophenyl)-*N*-metil-2-phenylquinazolin-4-amine (10l): According to TP1, the following amount of each reagent was used: 6-bromo-4-chloro-2-phenylquinazoline (0.29 mmol, 93 mg), 3-bromo-*N*-methylaniline (0.30 mmol, 56 mg). Reaction time: 10 min; mp (ºC): 160 – 161 °C; Appearance: White solid; Yield: 100 mg (0.21 mmol, 73%); eluent: Hexanes/Ethyl acetate (19:1); ^{1}H NMR (400 MHz, $CDCl_3$, ppm) δ 8.61 – 8.58 (m, 2H), 7.80 (d, *J* = 8.9 Hz, 1H), 7.67 (dd, *J* = 8.9, 2.2 Hz, 1H), 7.54 – 7.49 (m, 3H), 7.45 (ddd, *J* = 8.0, 1.8, 1.0 Hz, 1H), 7.40 (t, *J* = 2.0 Hz, 1H), 7.26 (t, *J* = 8.0 Hz, 1H), 7.17 (d, *J* = 2.1 Hz, 1H), 7.09 (ddd, *J* = 8.0, 2.1, 0.9 Hz, 1H), 3.74 (s, 3H); ^{13}C NMR (100 MHz, $CDCl_3$, ppm) δ 160.9, 160.1, 151.6, 149.5, 138.3, 135.7, 131.3, 130.8, 130.7, 129.7, 128.9,

128.7, 128.6 (4C), 124.4, 123.6, 118.0, 116.6, 42.4; IR (ATR, cm^{-1}): 3039, 2919, 1529, 1488, 1376, 1315, 1070, 1012, 874, 827, 770, 700; GC-MS (EI, 70 eV) *m/z* (%): 471 (36), 469 (73), 467 (36), 390 (14), 388 (16), 286 (56), 284 (56), 186 (41), 104 (44), 77 (100); HRMS (ESI/Q-TOF) *m/z*, calculated for $C_{21}H_{16}Br_2N_3^+$ $[M+H]^+$: 467.9705; found 467.9708.

***N*-(4-Fluorophenyl)-6-iodo-*N*-methyl-2-phenylquinazolin-4-amine (10m)**: According to TP1, the following amount of each reagent was used: 4-chloro-6-iodo-2-phenylquinazoline (0.30 mmol, 110 mg), 4-fluoro-*N*-methylaniline (0.31 mmol, 39 mg). Reaction time: 40 min; mp (ºC): 169 – 171 °C; Appearance: Pale yellow solid; Yield: 115 mg (0.25 mmol, 84%); eluent: Hexanes/Ethyl acetate (19:1); ^{1}H NMR (400 MHz, $CDCl_3$, ppm) δ 8.61 – 8.58 (m, 2H), 7.79 (dd, *J* = 8.8, 1.9 Hz, 1H), 7.63 (d, *J* = 8.8 Hz, 1H), 7.54 – 7.49 (m, 3H), 7.29 (d, *J* = 1.9 Hz, 1H), 7.21 – 7.11 (m, 4H), 3.72 (s, 3H); ^{13}C NMR (100 MHz, $CDCl_3$, ppm) δ 161.3 (d, *J* = 248.3 Hz), 160.4 (2C), 151.9, 144.1 (d, *J* = 2.3 Hz), 140.6, 138.4, 135.6, 130.7, 130.6, 128.6 (2C), 128.6 (2C), 127.9 (d, *J* = 8.4 Hz, 2C), 117.2 (d, *J* = 22.7 Hz, 2C), 116.9, 88.7, 42.7; IR (ATR, cm^{-1}): 3075, 2924, 1482, 1367, 1218, 1094, 833, 707; GC-MS (EI, 70 eV) *m/z* (%): 455 (100), 332 (31), 227 (15), 205 (31), 124 (65), 101 (27), 77 (19); HRMS (ESI/Q-TOF) *m/z*, calculated for $C_{21}H_{16}FIN_3^+$ $[M+H]^+$: 456.0367; found 456.0365.

6-Bromo-*N*-(4-fluorophenyl)-*N*-methyl-2-phenylquinazolin-4-amine (10n): According to TP1, the following amount of each reagent was used: 6-bromo-4-chloro-2-phenylquinazoline (0.23 mmol, 74 mg), 4-fluoro-*N*-methylaniline (0.24 mmol, 30 mg). Reaction time: 40 min; mp (ºC): 159 – 161 °C; Appearance: White solid; Yield: 70 mg (0.17 mmol, 75%); eluent: Hexanes/Ethyl acetate (19:1); ^{1}H NMR (400 MHz, $CDCl_3$, ppm) δ 8.61 – 8.58 (m, 2H), 7.77 (d, *J* = 8.9 Hz, 1H), 7.63

(dd, J = 8.9, 2.2 Hz, 1H), 7.54 – 7.49 (m, 3H), 7.21 – 7.11 (m, 4H), 7.09 (d, J = 2.1 Hz, 1H), 3.72 (s, 3H); ^{13}C NMR (100 MHz, $CDCl_3$, ppm) δ 161.3 (d, J = 247.7 Hz), 160.8 (2C), 151.6, 144.2 (d, J = 2.0 Hz), 138.4, 135.4, 130.8, 130.6, 128.8, 128.6 (4C), 127.9 (d, J = 8.4 Hz, 2C), 117.6, 117.3 (d, J = 22.7 Hz, 2C), 116.4, 42.8; IR (ATR, cm^{-1}): 3077, 2934, 1485, 1368, 1219, 1095, 835, 707; GC-MS (EI, 70 eV) *m/z* (%): 409 (79), 407 (77), 286 (40), 284 (38), 205 (26), 124 (100), 104 (30), 77 (68); HRMS (ESI/Q-TOF) *m/z*, calculated for $C_{21}H_{16}BrFN_3^+$ [M+H]$^+$: 408.0506; found 408.0506.

***N*-(3-Fluorophenyl)-6-iodo-*N*-methyl-2-phenylquinazolin-4-amine (10o)**: According to TP1, the following amount of each reagent was used: 4-chloro-6-iodo-2-phenylquinazoline (0.23 mmol, 84 mg), 3-fluoro-*N*-methylaniline (0.24 mmol, 30 mg). Reaction time: 20 min; mp (ºC): 151 – 153 °C; Appearance: Yellow solid; Yield: 75 mg (0.16 mmol, 72%); eluent: Hexanes/Ethyl acetate (9:1); ^{1}H NMR (400 MHz, $CDCl_3$, ppm) δ 8.61 – 8.58 (m, 2H), 7.82 (dd, J = 8.8, 1.9 Hz, 1H), 7.66 (d, J = 8.8 Hz, 1H), 7.55 – 7.49 (m, 3H), 7.40 – 7.34 (m, 2H), 7.05 (tdd, J = 8.3, 2.3, 1.0 Hz, 1H), 6.97 – 6.93 (m, 2H), 3.75 (s, 3H); ^{13}C NMR (100 MHz, $CDCl_3$, ppm) δ 163.7 (d, J = 248.3 Hz), 160.6, 160.2, 151.8, 149.6 (d, J = 10.0 Hz), 140.9, 138.3, 135.5, 131.3 (d, J = 9.2 Hz), 130.7 (2C), 128.6 (2C), 128.6 (2C), 121.5 (d, J = 2.1 Hz), 117.1, 113.7 (d, J = 21.3 Hz), 113.2 (d, J = 22.9 Hz), 89.0, 42.3; IR (ATR, cm^{-1}): 3073, 2925, 1549, 1479, 1389, 1363, 1307, 1095, 1069, 952, 834, 791, 702; GC-MS (EI, 70 eV) *m/z* (%): 455 (100), 332 (40), 227 (13), 205 (37), 164 (16), 124 (61), 101 (34), 77 (24); HRMS (ESI/Q-TOF) *m/z*, calculated for $C_{21}H_{16}FIN_3^+$ [M+H]$^+$: 456.0367; found 456.0368.

6-Bromo-*N*-(3-fluorophenyl)-*N*-methyl-2-phenylquinazolin-4-amine (10p): According to TP1, the following amount of each reagent was used: 6-bromo-4-

chloro-2-phenylquinazoline (0.35 mmol, 112 mg), 3-fluoro-*N*-methylaniline (0.36 mmol, 45 mg). Reaction time: 10 min; mp (ºC): 133 – 135 °C; Appearance: White solid; Yield: 100 mg (0.24 mmol, 70%); eluent: Hexanes/Ethyl acetate (19:1); ^{1}H NMR (400 MHz, $CDCl_3$, ppm) δ 8.61 – 8.58 (m, 2H), 7.79 (d, *J* = 8.9 Hz, 1H), 7.66 (dd, *J* = 8.9, 2.2 Hz, 1H), 7.55 – 7.49 (m, 3H), 7.39 – 7.33 (m, 1H), 7.18 (d, *J* = 2.1 Hz, 1H), 7.03 (tdd, *J* = 8.3, 2.4, 1.0 Hz, 1H), 6.97 – 6.93 (m, 2H), 3.75 (s, 3H); ^{13}C NMR (100 MHz, $CDCl_3$, ppm) δ 163.7 (d, *J* = 249.0 Hz), 161.0, 160.2, 151.6, 149.7 (d, *J* = 9.3 Hz), 138.3, 135.6, 131.4 (d, *J* = 9.4 Hz), 130.8, 130.7, 128.7, 128.6 (4C), 121.5 (d, *J* = 2.1 Hz), 117.9, 116.7, 113.7 (d, *J* = 21.3 Hz), 113.1 (d, *J* = 22.9 Hz), 42.4; IR (ATR, cm^{-1}): 3073, 2922, 1552, 1480, 1390, 1308, 1096, 952, 836, 791, 701; GC-MS (EI, 70 eV) *m/z* (%): 409 (73), 407 (74), 286 (50), 284 (49), 205 (31), 124 (100), 104 (36), 77 (85); HRMS (ESI/Q-TOF) *m/z*, calculated for $C_{21}H_{16}BrFN_3^+$ $[M+H]^+$: 408.0506; found 408.0507.

6-Iodo-2-phenyl-*N*-(2-tolyl)quinazolin-4-amine (15a): According to TP1, the following amount of each reagent was used: 4-chloro-6-iodo-2-phenylquinazoline (0.47 mmol, 172 mg), 2-toluidine (0.49 mmol, 52 mg). Reaction time: 2 h; mp (ºC): 203 – 205 °C; Appearance: White solid; Yield: 153 mg (0.35 mmol, 74%); eluent: Hexanes/Ethyl acetate (9:1); ^{1}H NMR (400 MHz, $CDCl_3$, ppm) δ 8.43 – 8.40 (m, 2H), 8.19 (d, *J* = 1.8 Hz, 1H), 8.01 (dd, *J* = 8.8, 1.8 Hz, 1H), 7.96 (d, *J* = 8.1 Hz, 1H), 7.71 (d, *J* = 8.8 Hz, 1H), 7.45 – 7.42 (m, 3H), 7.36 – 7.32 (m, 2H), 7.23 – 7.19 (m, 2H), 2.38 (s, 3H); ^{13}C NMR (100 MHz, $CDCl_3$, ppm) δ 160.9, 156.7, 150.5, 141.7, 138.3, 136.4, 131.5, 131.2, 130.9, 130.6, 129.8, 128.6 (2C), 128.5 (2C), 126.8, 125.8, 124.7, 115.9, 90.1, 18.4; IR (ATR, cm^{-1}): 3425, 3025, 1557, 1415, 1358, 1064, 831, 740, 708; GC-MS (EI, 70 eV) *m/z* (%): 437 (100), 422

(23), 334 (32), 295 (14), 206 (20), 155 (22), 101 (26), 77 (26); HRMS (ESI/Q-TOF) *m/z*, calculated for $C_{21}H_{17}IN_3^+$ [M+H]$^+$: 438.0462; found 438.0462.

6-Bromo-2-phenyl-*N*-(2-tolyl)quinazolin-4-amine (15b) (CAS: 449195-85-1): According to TP1, the following amount of each reagent was used: 6-bromo-4-chloro-2-phenylquinazoline (0.36 mmol, 115 mg), 2-toluidine (0.37 mmol, 40 mg). Reaction time: 2 h; mp (ºC): 176 – 178 °C; Appearance: White solid; Yield: 110 mg (0.28 mmol, 78%); eluent: Hexanes/Ethyl acetate (19:1); ^{1}H NMR (400 MHz, $CDCl_3$, ppm) δ 8.44 – 8.40 (m, 2H), 8.0 – 7.97 (m, 2H), 7.85 (d, *J* = 1.1 Hz, 2H), 7.45 – 7.42 (m, 3H), 7.37 – 7.32 (m, 2H), 7.22 (dd, *J* = 7.4, 1.0 Hz, 1H), 7.20 – 7.17 (m, 1H), 2.39 (s, 3H); ^{13}C NMR (100 MHz, $CDCl_3$, ppm) δ 160.8, 157.0, 150.1, 138.3, 136.5, 136.3, 131.3, 131.2, 130.9, 130.6, 128.6 (2C), 128.5 (2C), 126.8, 125.7, 124.6, 123.3, 119.2, 115.2, 18.4; IR (ATR, cm^{-1}): 3058, 2977, 1551, 1518, 1356, 1308, 872, 826, 745, 705; GC-MS (EI, 70 eV) *m/z* (%): 391 (92), 389 (96), 375 (21), 373 (24), 288 (39), 286 (42), 206 (28), 155 (23), 104 (40), 77 (100); HRMS (ESI/Q-TOF) *m/z*, calculated for $C_{21}H_{17}BrN_3^+$ [M+H]$^+$: 390.0600; found 390.0601.

***N*-(2-Fluorophenyl)-6-iodo-2-phenylquinazolin-4-amine (15c)**: According to TP1, the following amount of each reagent was used: 4-chloro-6-iodo-2-phenylquinazoline (0.45 mmol, 165 mg), 2-fluoroaniline (0.46 mmol, 51 mg). Reaction time: 40 min; mp (ºC): 169 – 170 °C; Appearance: White solid; Yield: 120 mg (0.27 mmol, 60%); eluent: Hexanes/Ethyl acetate (19:1); ^{1}H NMR (400 MHz, $CDCl_3$, ppm) δ 8.74 (td, *J* = 8.2, 1.6 Hz, 1H), 8.50 – 8.46 (m, 2H), 8.19 (d, *J* = 1.7 Hz, 1H), 8.01 (dd, *J* = 8.8, 1.8 Hz, 1H), 7.69 (d, *J* = 8.8 Hz, 1H), 7.56 (d, *J* = 3.2 Hz, 1H), 7.51 – 7.47 (m, 3H), 7.29 (t, *J* = 7.8 Hz, 1H), 7.21 (ddd, *J* = 11.1, 8.2, 1.5 Hz, 1H), 7.16 – 7.10 (m, 1H); ^{13}C NMR (100 MHz, $CDCl_3$, ppm) δ 160.7,

155.8, 153.6 (d, *J* = 242.9 Hz), 150.4, 141.9, 138.3, 131.2, 130.8, 129.5, 128.7 (2C), 128.6 (2C), 127.0 (d, *J* = 9.3 Hz), 124.6 (d, *J* = 3.5 Hz), 124.3 (d, *J* = 7.8 Hz), 123.1, 116.0, 115.2 (d, *J* = 19.5 Hz), 90.7; IR (ATR, cm^{-1}): 3427, 3054, 1551, 1522, 1355, 1256, 1122, 826, 748, 699; GC-MS (EI, 70 eV) *m/z* (%): 441 (64), 422 (100), 295 (20), 204 (21), 101 (31), 77 (19); HRMS (ESI/Q-TOF) *m/z*, calculated for $C_{20}H_{14}FIN_3^+$ $[M+H]^+$: 442.0211; found 442.0211.

6-Bromo-*N*-(2-fluorophenyl)-2-phenylquinazolin-4-amine (15d): According to TP1, the following amount of each reagent was used: 6-bromo-4-chloro-2-phenylquinazoline (0.50 mmol, 160 mg), 2-fluoroaniline (0.51 mmol, 57 mg). Reaction time: 40 min; mp (ºC): 154 – 156 °C; Appearance: White solid; Yield: 110 mg (0.28 mmol, 56%); eluent: Hexanes/Ethyl acetate (98:2); ^{1}H NMR (400 MHz, $CDCl_3$, ppm) δ 8.76 (td, *J* = 8.2, 1.6 Hz, 1H), 8.50 – 8.48 (m, 2H), 8.00 (s, 1H), 7.85 (d, *J* = 1.0 Hz, 2H), 7.56 (d, *J* = 3.3 Hz, 1H), 7.51 – 7.48 (m, 3H), 7.30 (t, *J* = 7.8 Hz, 1H), 7.21 (ddd, *J* = 11.2, 8.2, 1.5 Hz, 1H), 7.16 – 7.10 (m, 1H); ^{13}C NMR (100 MHz, $CDCl_3$, ppm) δ 160.7, 156.2, 153.6 (d, *J* = 244.1 Hz), 150.0, 138.3, 136.6, 131.3, 130.7, 128.6 (4C), 127.1 (d, *J* = 9.2 Hz), 124.6 (d, *J* = 2.9 Hz), 124.3 (d, *J* = 7.4 Hz), 123.1 (d, *J* = 10.2 Hz), 119.7, 115.3 (2C), 115.2 (d, *J* = 19.4 Hz); IR (ATR, cm^{-1}): 3449, 3027, 1553, 1523, 1401, 1358, 1318, 1183, 829, 736, 697; GC-MS (EI, 70 eV) *m/z* (%): 395 (41), 393 (42), 376 (89), 374 (100), 147 (14), 101 (18), 77 (80); HRMS (ESI/Q-TOF) *m/z*, calculated for $C_{20}H_{14}BrFN_3^+$ $[M+H]^+$: 394.0350; found 394.0347.

6-Bromo-*N*-(3-chlorophenyl)-2-phenylquinazolin-4-amine (15e) (CAS: 449195-91-9): According to TP1, the following amount of each reagent was used: 6-bromo-4-chloro-2-phenylquinazoline (0.50 mmol, 160 mg), 3-chloroaniline (0.53 mmol, 67 mg). Reaction time: 40 min; mp (ºC): 182.4 – 184.5 °C;

Appearance: White solid; Yield: 189 mg (0.46 mmol, 92%); eluent: No further purification process was needed; ^{1}H NMR (400 MHz, DMSO-d_6, ppm) δ 10.00 (s, 1H), 8.84 (d, *J* = 2.0 Hz, 1H), 8.43 – 8.40 (m, 2H), 8.24 (t, *J* = 2.0 Hz, 1H), 7.97 (dd, *J* = 8.9, 2.1 Hz, 1H), 7.92 – 7.89 (m, 1H), 7.79 (d, *J* = 8.9 Hz, 1H), 7.52 – 7.46 (m, 4H), 7.23 – 7.20 (m, 1H); ^{13}C NMR (100 MHz, DMSO-d_6, ppm) δ 159.3, 156.8, 149.4, 140.7, 137.8, 136.3, 132.8, 130.7, 130.4, 130.2, 128.5 (2C), 127.9 (2C), 125.5, 123.2, 121.5, 120.1, 118.6, 115.3; IR (ATR, cm^{-1}): 3029, 1568, 1555, 1520, 1427, 1417, 1355, 1235, 829, 763, 698; HRMS (ESI/Q-TOF) *m/z*, calculated for $C_{20}H_{14}BrClN_3^+$ $[M+H]^+$: 412.0034; found 412.0018.

6-Bromo-*N*-(4-fluorophenyl)-2-phenylquinazolin-4-amine (15f) (CAS: 449196-33-2): According to TP1, the following amount of each reagent was used: 6-bromo-4-chloro-2-phenylquinazoline (0.50 mmol, 160 mg), 4-fluoroaniline (0.53 mmol, 58 mg). Reaction time: 40 min; mp (ºC): 180.3 – 182.5 °C; Appearance: White solid; Yield: 188 mg (0.48 mmol, 95%); eluent: Hexanes/Ethyl acetate (9:1); ^{1}H NMR (400 MHz, DMSO-d_6, ppm) δ 9.97 (s, 1H), 8.83 (d, *J* = 2.0 Hz, 1H), 8.41 – 8.38 (m, 2H), 7.98 – 7.92 (m, 3H), 7.78 (d, *J* = 8.9 Hz, 1H), 7.51 – 7.49 (m, 3H), 7.31 (t, *J* = 8.9 Hz, 2H); ^{13}C NMR (100 MHz, DMSO-d_6, ppm) δ 159.4, 158.5 (d, *J* = 240.7 Hz, 1C), 157.0, 149.4, 137.9, 136.2, 135.3 (d, *J* = 2.6 Hz, 1C), 130.5, 130.3, 128.5 (2C), 128.0 (2C), 125.5, 124.3 (d, *J* = 8.0 Hz, 2C), 118.4, 115.2, 115.2 (d, *J* = 22.3 Hz, 2C); IR (ATR, cm^{-1}): 3047, 3027, 1559, 1526, 1508, 1417, 1357, 1223, 1156, 826, 773, 696; HRMS (ESI/Q-TOF) *m/z*, calculated for $C_{20}H_{14}BrFN_3^+$ $[M+H]^+$: 394.0350; found 394.0353.

6-Bromo-*N*-(4-methoxyphenyl)-2-phenylquinazolin-4-amine (15g) (CAS: 449195-96-4): According to TP1, the following amount of each reagent was used: 6-bromo-4-chloro-2-phenylquinazoline (0.50 mmol, 160 mg), 4-methoxyaniline (0.53 mmol, 65 mg). Reaction time: 40 min; Appearance: Yellow oil; Yield: 195 mg (0.48 mmol, 96%); eluent: Hexanes/Ethyl acetate (9:1); ^{1}H NMR (400 MHz, $CDCl_3$, ppm) δ 8.41 – 8.36 (m, 2H), 7.89 (s, 1H), 7.76 – 7.71 (m, 2H), 7.62 (d, *J* = 9.0 Hz, 2H), 7.40 – 7.37 (m, 3H), 7.24 (s, 1H), 6.90 (d, *J* = 9.0 Hz, 2H), 3.78 (s, 3H); ^{13}C NMR (100 MHz, $CDCl_3$, ppm) δ 160.9, 156.7, 156.7, 149.8, 138.4, 136.2, 131.3, 131.0, 130.6, 128.6 (2C), 128.5 (2C), 123.7 (2C), 123.3, 119.1, 115.1, 114.3 (2C), 55.7; IR (ATR, cm^{-1}): 2925, 1560, 1558, 1507, 1420, 1229, 829, 707, 668; HRMS (ESI/Q-TOF) *m/z*, calculated for $C_{21}H_{17}BrN_3O^+$ [M+H]$^+$: 406.0550; found 406.0556.

6-Iodo-*N*-methyl-2-phenyl-*N*-(2-tolyl)quinazolin-4-amine (10g): According to TP2, the following amount of each reagent was used: 6-iodo-2-phenyl-*N*-(2-tolyl)quinazolin-4-amine (0.20 mmol, 88 mg), NaH (0.40 mmol, 16 mg), DMF (3 mL), CH_3I (4.8 mmol, 0.30 mL); mp (ºC): 185 – 187 °C; Appearance: Pale yellow solid; Yield: 80 mg (0.18 mmol, 89%); eluent: Hexanes/Ethyl acetate (9:1); ^{1}H NMR (400 MHz, $CDCl_3$, ppm) δ 8.62 – 8.60 (m, 2H), 7.76 (dd, *J* = 8.8, 1.9 Hz, 1H), 7.60 (d, *J* = 8.8 Hz, 1H), 7.54 – 7.48 (m, 3H), 7.40 – 7.38 (m, 2H), 7.35 – 7.30 (m, 1H), 7.18 (d, *J* = 7.5 Hz, 1H), 7.01 (d, *J* = 1.9 Hz, 1H), 3.68 (s, 3H), 2.18 (s, 3H); ^{13}C NMR (100 MHz, $CDCl_3$, ppm) δ 160.1, 159.7, 151.7, 145.8, 140.4, 138.7, 135.4, 134.7, 132.2, 130.6, 130.5, 128.6 (2C), 128.5 (3C), 128.2, 127.8, 116.9, 88.5, 41.3, 17.9; IR (ATR, cm^{-1}): 3058, 2923, 1483, 1369, 1091, 826, 769, 702; GC-MS (EI, 70 eV) *m/z* (%): 451 (79), 436 (100), 333 (32), 309 (19), 225

(18), 205 (25), 120 (43), 104 (47), 77 (25); HRMS (ESI/Q-TOF) *m/z*, calculated for $C_{22}H_{19}IN_3^+$ $[M+H]^+$: 452.0618; found 452.0618.

6-Bromo-*N*-methyl-2-phenyl-*N*-(2-tolyl)quinazolin-4-amine (10h): According to TP2, the following amount of each reagent was used: 6-Bromo-2-phenyl-*N*-(2-tolyl)quinazolin-4-amine (0.13 mmol, 51 mg), NaH (0.26 mmol, 10 mg), DMF (2 mL), CH_3I (3.1 mmol, 0.19 mL); mp (ºC): 186 – 188 °C; Appearance: White solid; Yield: 40 mg (0.10 mmol, 76%); eluent: Hexanes/Ethyl acetate (19:1); ^1H NMR (400 MHz, $CDCl_3$, ppm) δ 8.63 – 8.60 (m, 2H), 7.75 (d, *J* = 8.9 Hz, 1H), 7.60 (dd, *J* = 8.9, 2.2 Hz, 1H), 7.54 – 7.49 (m, 3H), 7.39 – 7.37 (m, 2H), 7.33 – 7.29 (m, 1H), 7.18 (d, *J* = 7,6 Hz, 1H), 6.80 (d, *J* = 2.1 Hz, 1H), 3.68 (s, 3H), 2.19 (s, 3H); ^{13}C NMR (100 MHz, $CDCl_3$, ppm) δ 160.1, 160.0, 151.4, 145.9, 138.7, 135.4, 135.2, 132.2, 130.5, 130.5, 128.5 (2C), 128.5 (3C), 128.2, 127.9, 127.9, 117.4, 116.4, 41.4, 17.9; IR (ATR, cm^{-1}): 3059, 2922, 1485, 1369, 1092, 828, 703; GC-MS (EI, 70 eV) *m/z* (%): 405 (53), 403 (53), 390 (96), 388 (100), 285 (50), 120 (71), 104 (85), 77 (83); HRMS (ESI/Q-TOF) *m/z*, calculated for $C_{22}H_{19}BrN_3^+$ $[M+H]^+$: 404.0757; found 404.0757.

***N*-(2-Fluorophenyl)-6-iodo-*N*-methyl-2-phenylquinazolin-4-amine (10q)**: According to TP2, the following amount of each reagent was used: *N*-(2-fluorophenyl)-6-iodo-2-phenylquinazolin-4-amine (0.25 mmol, 110 mg), NaH (0.50 mmol, 20 mg), DMF (4 mL), CH_3I (6.0 mmol, 0.37 mL); mp (ºC): 152 – 154 °C; Appearance: White solid; Yield: 93 mg (0.20 mmol, 82%); eluent: Hexanes/Ethyl acetate (19:1); ^1H NMR (400 MHz, $CDCl_3$, ppm) δ 8.62 – 8.60 (m, 2H), 7.80 (dd, *J* = 8.8, 1.9 Hz, 1H), 7.64 (d, *J* = 8.8 Hz, 1H), 7.54 – 7.49 (m, 3H), 7.42 – 7.37 (m, 1H), 7.29 (d, *J* = 1.8 Hz, 1H), 7.27 – 7.21 (m, 3H), 3.73 (s, 3H); ^{13}C NMR (100 MHz, $CDCl_3$, ppm) δ 160.4, 160.0, 157.8 (d, *J* = 251.5 Hz), 151.7,

140.7, 138.5, 135.2 (d, J = 12.1 Hz), 134.4, 130.8, 130.6, 129.1 (d, J = 7.8 Hz, 2C), 128.7, 128.6, 128.5 (2C), 125.7 (d, J = 2.8 Hz), 117.6 (d, J = 19.9 Hz), 116.9, 88.8, 41.4; IR (ATR, cm^{-1}): 3062, 2928, 1529, 1482, 1368, 1313, 1091, 839, 756, 704; GC-MS (EI, 70 eV) *m/z* (%): 455 (100), 436 (100), 332 (21), 205 (37), 124 (70), 101 (40), 77 (40); HRMS (ESI/Q-TOF) *m/z*, calculated for $C_{21}H_{16}FIN_3^+$ $[M+H]^+$: 456.0367; found 456.0368.

6-Bromo-*N*-(2-fluorophenyl)-*N*-methyl-2-phenylquinazolin-4-amine (10r): According to TP2, the following amount of each reagent was used: 6-Bromo-*N*-(2-fluorophenyl)-2-phenylquinazolin-4-amine (0.25 mmol, 99 mg), NaH (0.50 mmol, 20 mg), DMF (4 mL), CH_3I (6.0 mmol, 0.37 mL); mp (ºC): 150 – 152 °C; Appearance: White solid; Yield: 80 mg (0.20 mmol, 78%); eluent: Hexanes/Ethyl acetate (19:1); ^1H NMR (400 MHz, $CDCl_3$, ppm) δ 8.62 – 8.60 (m, 2H), 7.79 (d, J = 8.9 Hz, 1H), 7.65 (dd, J = 8.9, 2.2 Hz, 1H), 7.54 – 7.49 (m, 3H), 7.41 – 7.35 (m, 1H), 7.27 – 7.21 (m, 3H), 7.08 (d, J = 2.1 Hz, 1H), 3.73 (s, 3H); ^{13}C NMR (100 MHz, $CDCl_3$, ppm) δ 160.8, 160.0, 157.8 (d, J = 250.3 Hz), 151.4, 138.5, 135.4, 135.3 (d, J = 11.6 Hz), 130.8, 130.6, 129.1 (d, J = 7.4 Hz, 2C), 128.7, 128.6 (2C), 128.5 (2C), 127.7, 125.7 (d, J = 3.2 Hz), 117.7 (d, J = 20.4 Hz), 116.4, 41.5; IR (ATR, cm^{-1}): 3064, 2925, 1532, 1484, 1370, 1314, 1091, 840, 758, 704; GC-MS (EI, 70 eV) *m/z* (%): 409 (52), 407 (56), 390 (76), 388 (76), 285 (27), 205 (24), 124 (93), 77 (100); HRMS (ESI/Q-TOF) *m/z*, calculated for $C_{21}H_{16}BrFN_3^+$ $[M+H]^+$: 408.0506; found 408.0507.

Synthesis of MPC-6827 – verubulin (4), 4-chloro-2-methylquinazoline (17) and derivatives 18–20.

4-Chloro-2-methylquinazoline (17) (CAS: 6484-24-8)**:** Synthesized in a manner analogous to [2]. In a two necked round-bottomed flask connected to a condenser, 2-methylquinazolin-4(3*H*)-one (10.0 mmol, 1.60 g), PPh_3 (30.0 mmol, 7.86 g), Cl_3CCN (15.0 mmol, 1.50 mL), and 50 mL of toluene were added. The reaction mixture was stirred at 110 °C for 20 min and after this time filtrated through a column containing silica gel using ethyl acetate as eluent. The filtrate was concentrated under reduced pressure and purified by flash column chromatography using a mixture of hexanes/ethyl acetate 4:1 as eluent. The product was obtained in 67% yield as pale yellow solid (1.19 g, 6.7 mmol). ^{1}H NMR (400 MHz, $CDCl_3$, ppm) δ 8.22 (ddd, *J* = 8.4, 1.2, 0.6 Hz, 1H), 7.96 (ddd, *J* = 8.4, 1.4, 0.6, 1H), 7.92 (ddd, *J* = 8.1, 6.7, 1.4 Hz, 1H), 7.65 (ddd, *J* = 8.2, 6.7, 1.4 Hz, 1H), 2.86 (s, 3H); ^{13}C NMR (100 MHz, $CDCl_3$, ppm) δ 163.6, 162.3, 151.6, 135.0, 128.1 (2C), 125.8, 121.9, 26.2.

***N*-(4-Methoxyphenyl)-*N*,2-dimethyl-quinazolin-4-amine (verubulin, 4),** (CAS: 827031-83-4): According to TP1, the following amount of each reagent was used: 4-chloro-2-methylquinazoline (0.50 mmol, 89 mg), 4-methoxy-*N*-methylaniline (0.51 mmol, 70 mg). Reaction time: 10 min; Appearance: Yellow solid; Yield: 126 mg (0.45 mmol, 90%); eluent: Hexanes/Ethyl acetate (4:1 to 1:1); ^{1}H NMR (400 MHz, $CDCl_3$, ppm) δ 7.73 (dd, *J* = 8.4, 0.6 Hz, 1H), 7.52 (ddd, *J* = 8.4, 6.7, 1.6 Hz, 1H), 7.11 (d, *J* = 9.0 Hz, 2H), 7.01 (ddd, *J* = 8.5, 1.6, 0.6 Hz, 1H), 6.96 (ddd, *J* = 8.0, 6.7, 1.3 Hz, 1H), 6.90 (d, *J* = 9.0 Hz, 2H), 3.83 (s, 3H), 3.58 (s, 3H), 2.72 (s, 3H); ^{13}C NMR (100 MHz, $CDCl_3$, ppm) δ 163.5, 161.8, 158.1, 152.3, 141.7,

131.8, 127.8, 127.4 (2C), 126.3, 124.0, 115.3 (2C), 114.8, 55.6, 42.8, 26.6; GC-MS (EI, 70 eV) *m/z* (%): 279 (100), 264 (14), 248 (8), 171 (21), 136 (49), 121 (42);

***N*-Methyl-*N*-phenylquinazolin-4-amine (18)** (CAS: 111157-70-1): According to TP1, the following amount of each reagent was used: 4-chloroquinazoline (0.50 mmol, 82 mg), *N*-methylaniline (0.53 mmol, 56 mg). Reaction time: 10 min; Appearance: Pale yellow solid; Yield: 95 mg (0.40 mmol, 81%); eluent: Hexanes/Ethyl acetate (1:1); ^{1}H NMR (400 MHz, $CDCl_3$, ppm) δ 8.85 (s, 1H), 7.83 (d, *J* = 8.3 Hz, 1H), 7.57 (ddd, *J* = 8.3, 5.9, 2.4 Hz, 1H), 7.40 – 7.35 (m, 2H), 7.27 (tt, *J* = 7.6, 1.2 Hz, 1H), 7.19 – 7.16 (m, 2H), 7.07 – 7.03 (m, 2H), 3.64 (s, 3H); ^{13}C NMR (100 MHz, $CDCl_3$, ppm) δ 162.0, 154.5, 151.7, 148.7, 132.0, 130.2 (2C), 128.6, 126.6, 126.4, 125.9 (2C), 125.1, 116.9, 42.5; GC-MS (EI, 70 eV) *m/z* (%): 235 (31), 234 (100), 219 (7), 106 (25), 77 (14).

***N*-(4-Fluorophenyl)-*N*,2-dimethylquinazolin-4-amine (19)** (CAS: 827031-62-9): According to TP1, the following amount of each reagent was used: 4-chloro-2-methylquinazoline (0.50 mmol, 89 mg), 4-fluoro-*N*-methylaniline (0.52 mmol, 65 mg). Reaction time: 10 min; Appearance: Pale yellow solid; Yield: 126 mg (0.47 mmol, 94%); eluent: Hexanes/Ethyl acetate (4:1 to 1:1); ^{1}H NMR (400 MHz, $CDCl_3$, ppm) δ 7.76 (d, *J* = 8.4 Hz, 1H), 7.57 – 7.52 (m, 1H), 7.16 – 7.11 (m, 2H), 7.09 – 7.04 (m, 2H), 7.01 – 6.99 (m, 2H), 3.60 (s, 3H), 2.74 (s, 3H); ^{13}C NMR (100 MHz, $CDCl_3$, ppm) δ 163.5, 161.9, 160.8 (d, *J* = 246.9 Hz, 1C), 152.3, 145.0, 132.0, 128.0, 127.6 (d, *J* = 8.4 Hz, 2C), 126.1, 124.3, 117.0 (d, *J* = 22.7 Hz, 2C), 114.7, 42.6, 26.6; GC-MS (EI, 70 eV) *m/z* (%): 267 (78), 266 (100), 251 (11), 144 (49), 124 (46), 103 (40).

***N*,2-Dimethyl-*N*-phenylquinazolin-4-amine (20):** According to TP1, the following amount of each reagent was used: 4-chloro-2-methylquinazoline (0.50 mmol, 89 mg), *N*-methylaniline (0.53 mmol, 56 mg). Reaction time: 10 min; mp (ºC): 79.2 – 80.9 °C; Appearance: Yellow solid; Yield: 118 mg (0.47 mmol, 95%); eluent: Hexanes/Ethyl acetate (4:1 to 1:1); ^{1}H NMR (400 MHz, $CDCl_3$, ppm) δ 7.75 (d, *J* = 8.3 Hz, 1H), 7.53 (ddd, *J* = 8.3, 6.7, 1.5 Hz, 1H), 7.38 – 7.34 (m, 2H), 7.27 – 7.23 (m, 1H), 7.17 – 7.15 (m, 2H), 7.01 (dd, *J* = 8.5, 1.4 Hz, 1H), 6.95 (ddd, *J* = 8.1, 6.7, 1.2 Hz, 1H), 3.63 (s, 3H), 2.74 (s, 3H); ^{13}C NMR (100 MHz, $CDCl_3$, ppm) δ 163.6, 162.0, 152.3, 148.9, 131.9, 130.0 (2C), 127.8, 126.3, 126.2, 125.9 (2C), 124.2, 115.0, 42.4, 26.6; IR (ATR, cm^{-1}): 2920, 1563, 1551, 1484, 1380, 1102, 775, 761, 704; GC-MS (EI, 70 eV) *m/z* (%): 249 (56), 248 (100), 233 (9), 144 (27), 106 (38), 77 (17); HRMS (ESI/Q-TOF) *m/z*, calculated for $C_{16}H_{16}N_3^+$ $[M+H]^+$: 250.1339; found 250.1338.

2. NMR spectra

^{1}H NMR (300 MHz, CD_3OD, ppm) of 2-amino-5-bromobenzamide (**6a**)

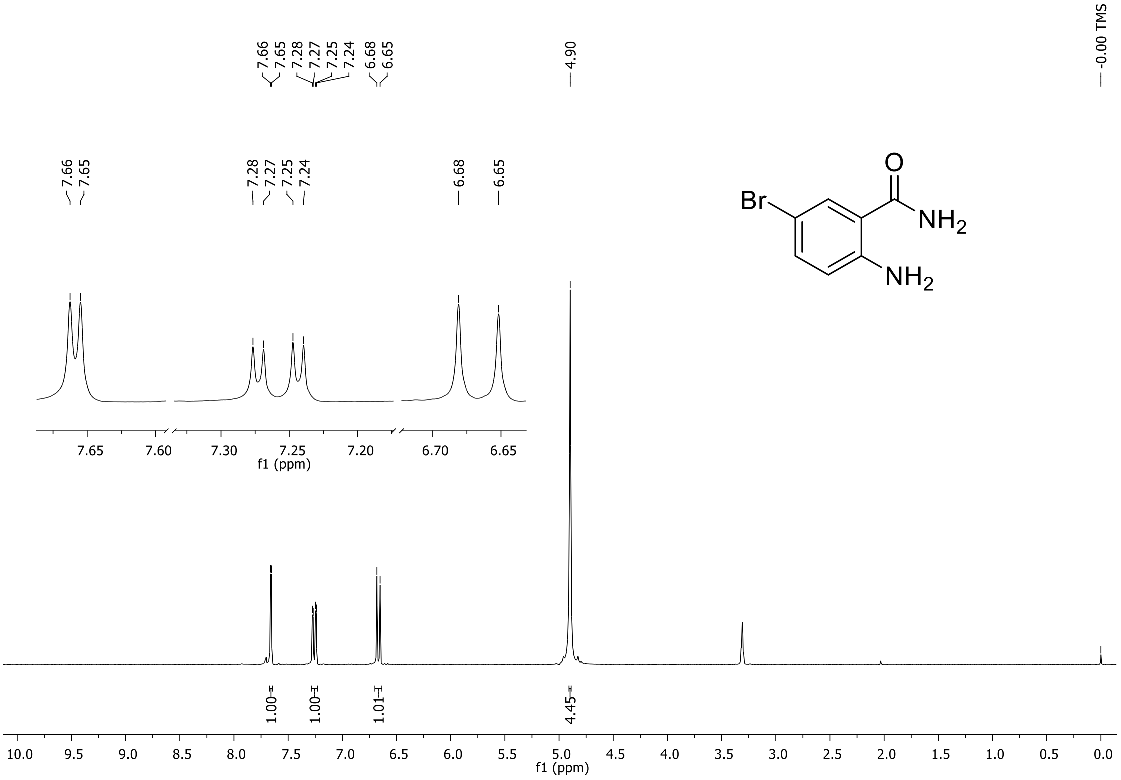

^{1}H NMR (500 MHz, $CDCl_3$, ppm) of 6-bromo-4-chloro-2-phenylquinazoline (**8a**)

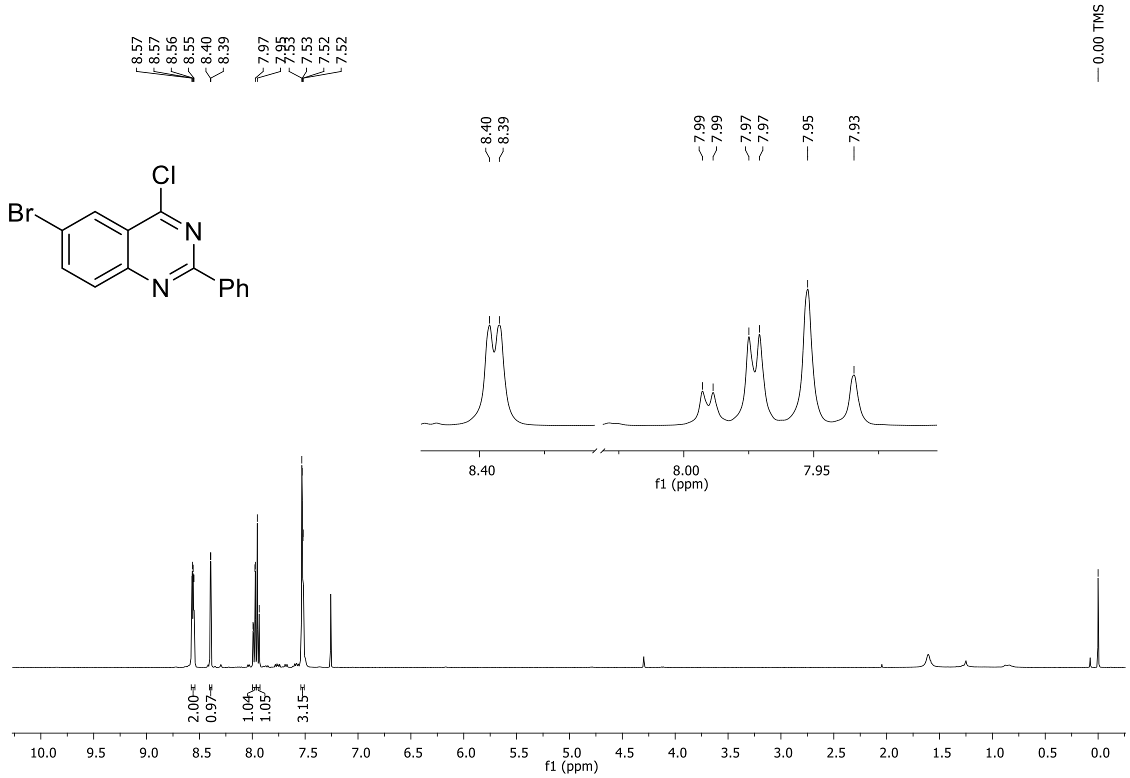

^{13}C NMR (125 MHz, $CDCl_3$, ppm) of 6-bromo-4-chloro-2-phenylquinazoline (**8a**)

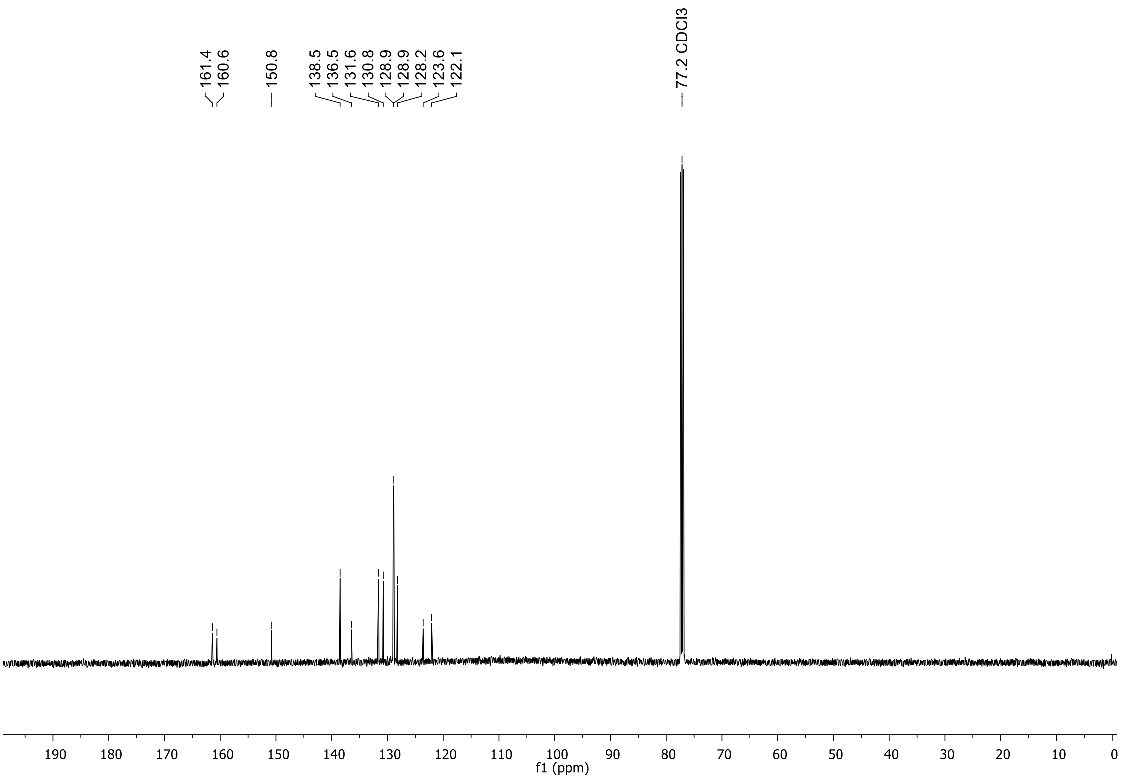

^{1}H NMR (500 MHz, DMSO-d6, ppm) of 2-amino-5-iodobenzamide (**6b**)

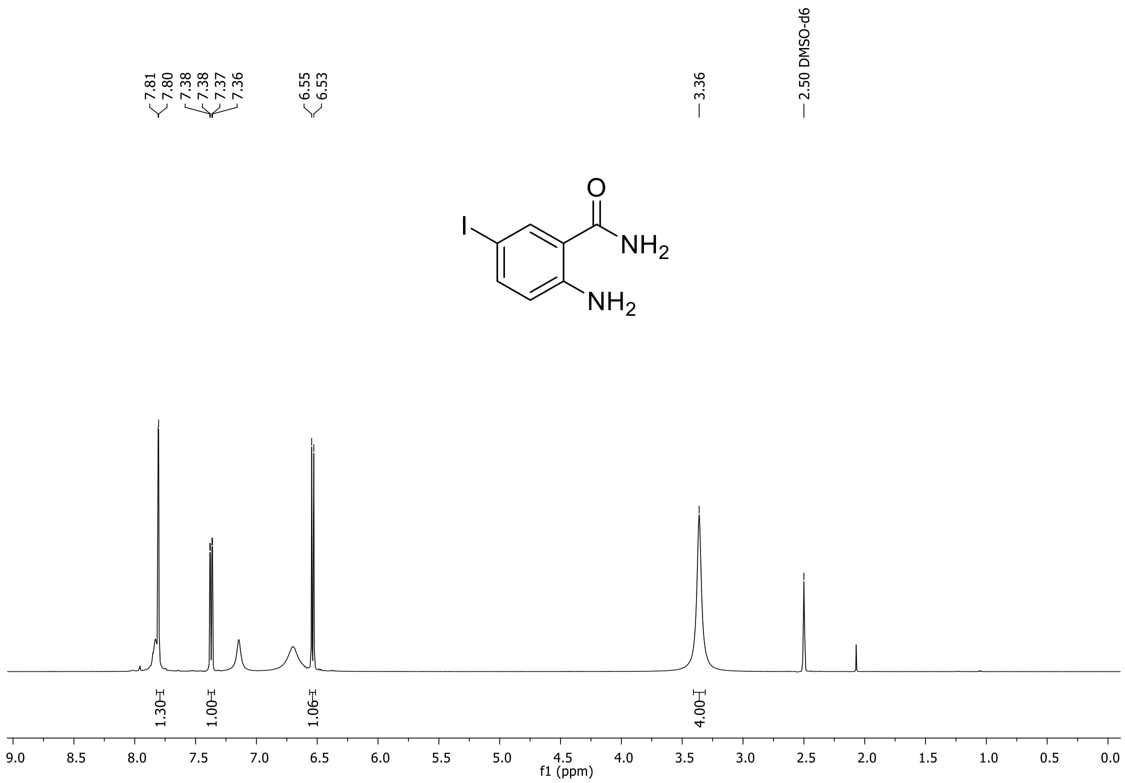

^{13}C NMR (125 MHz, DMSO-d6, ppm) of 2-amino-5-iodobenzamide (**6b**)

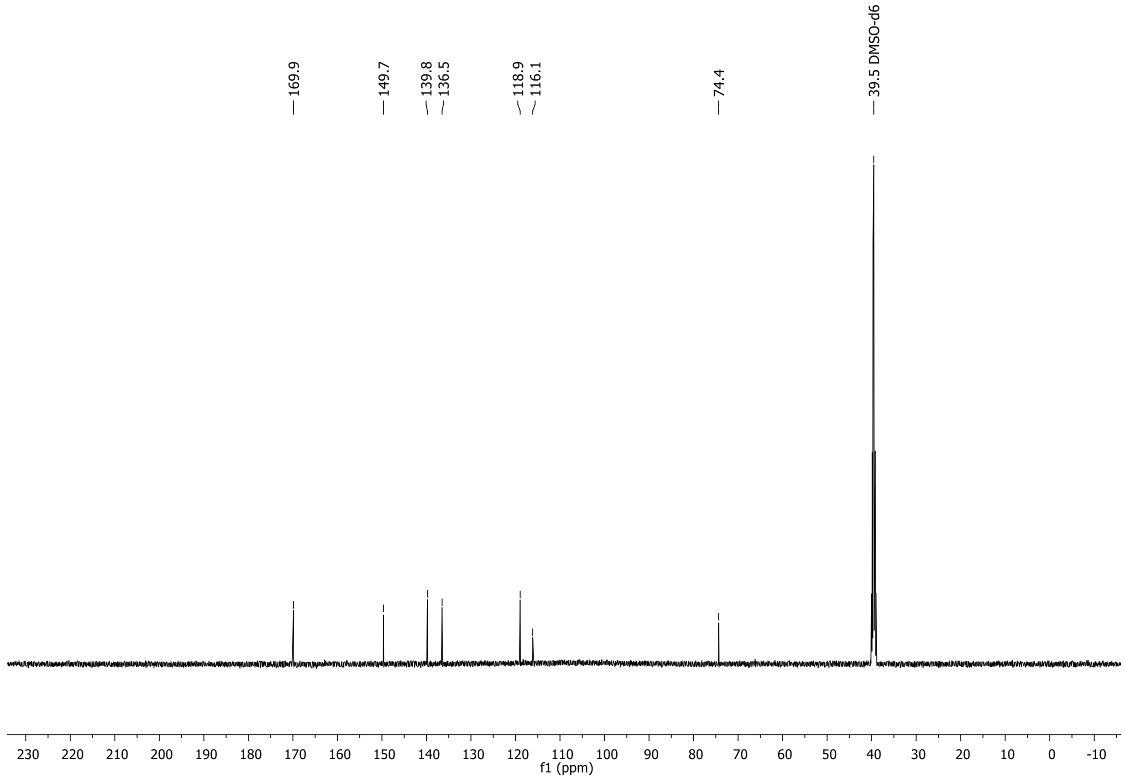

^{1}H NMR (500 MHz, $CDCl_3$, ppm) of 4-chloro-6-iodo-2-phenylquinazoline (**8b**)

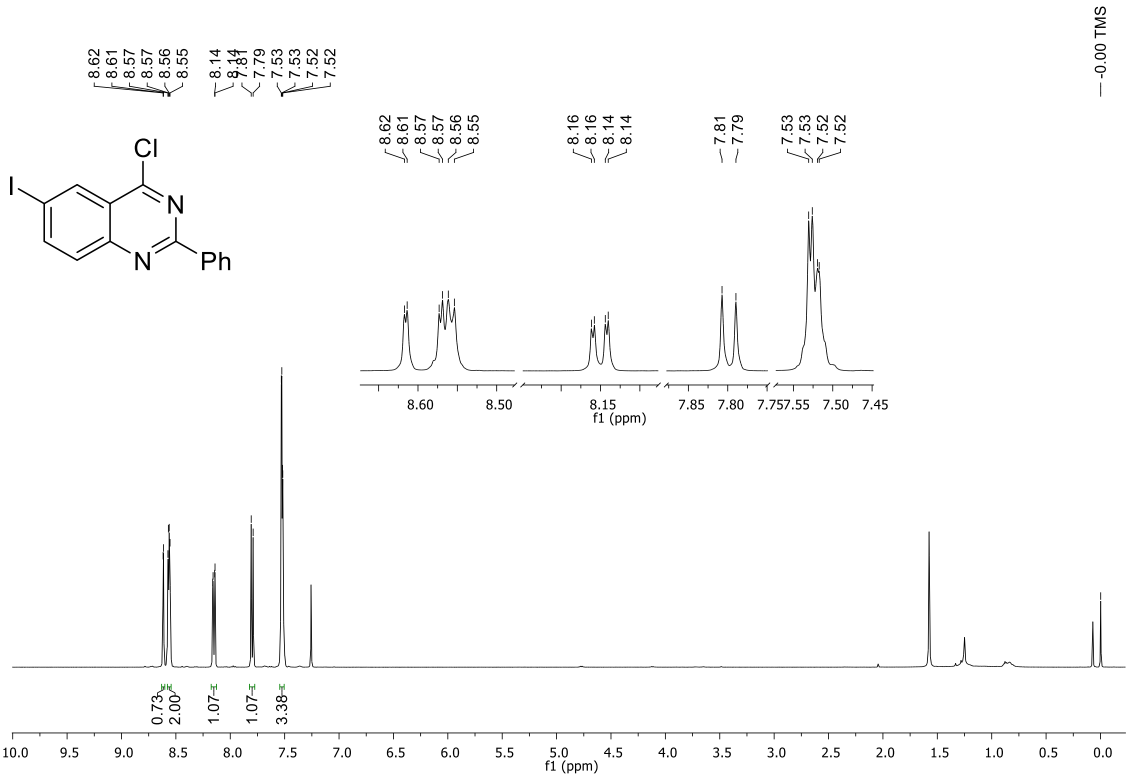

^{13}C NMR (125 MHz, $CDCl_3$, ppm) of 4-chloro-6-iodo-2-phenylquinazoline (**8b**)

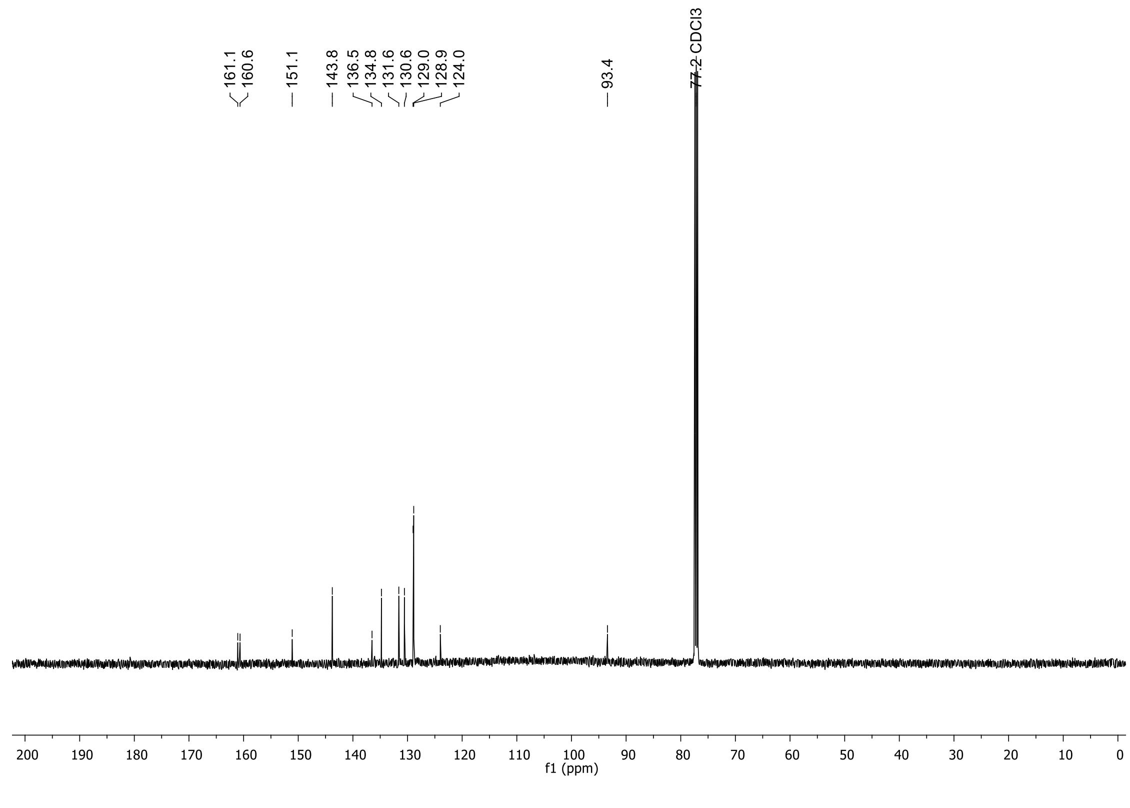

^{1}H NMR (400 MHz, $CDCl_3$, ppm) of 6-iodo-*N*-(4-methoxyphenyl)-*N*-methyl-2-phenylquinazolin-4-amine (**10a**)

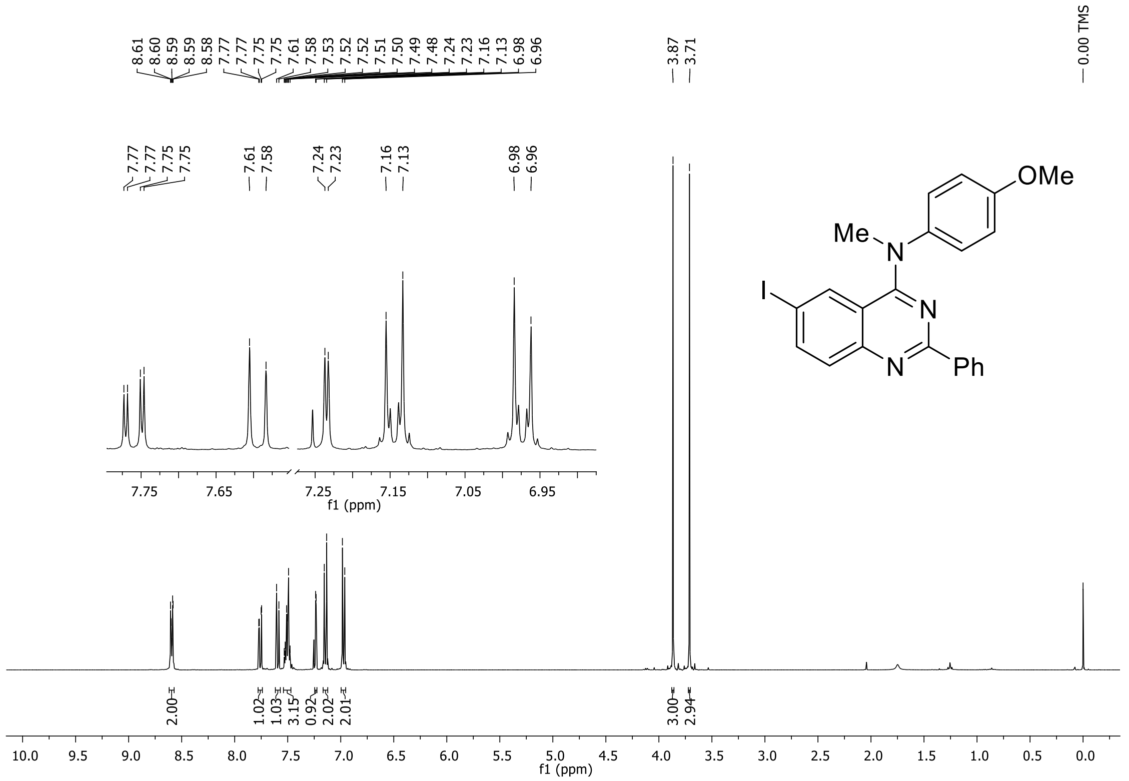

^{13}C NMR (100 MHz, $CDCl_3$, ppm) of 6-iodo-*N*-(4-methoxyphenyl)-*N*-methyl-2-phenylquinazolin-4-amine (**10a**)

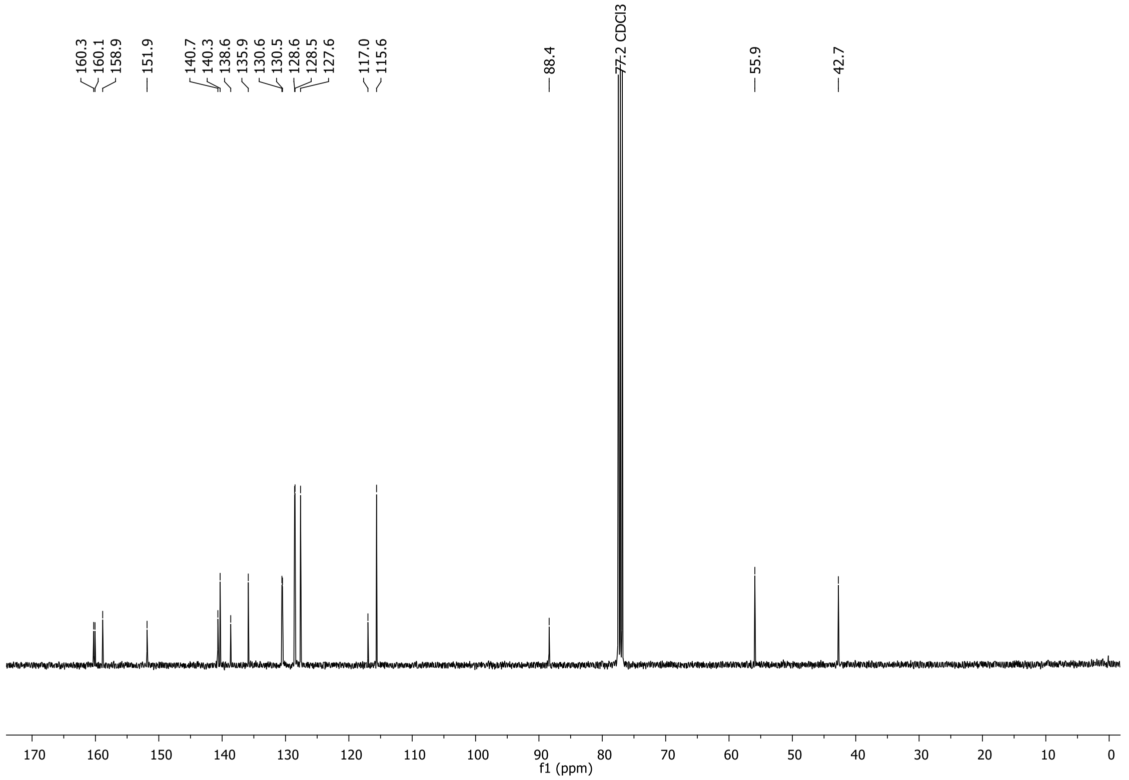

^{1}H NMR (400 MHz, $CDCl_3$, ppm) of 6-Bromo-*N*-(4-methoxyphenyl)-*N*-methyl-2-phenylquinazolin-4-amine (**10b**)

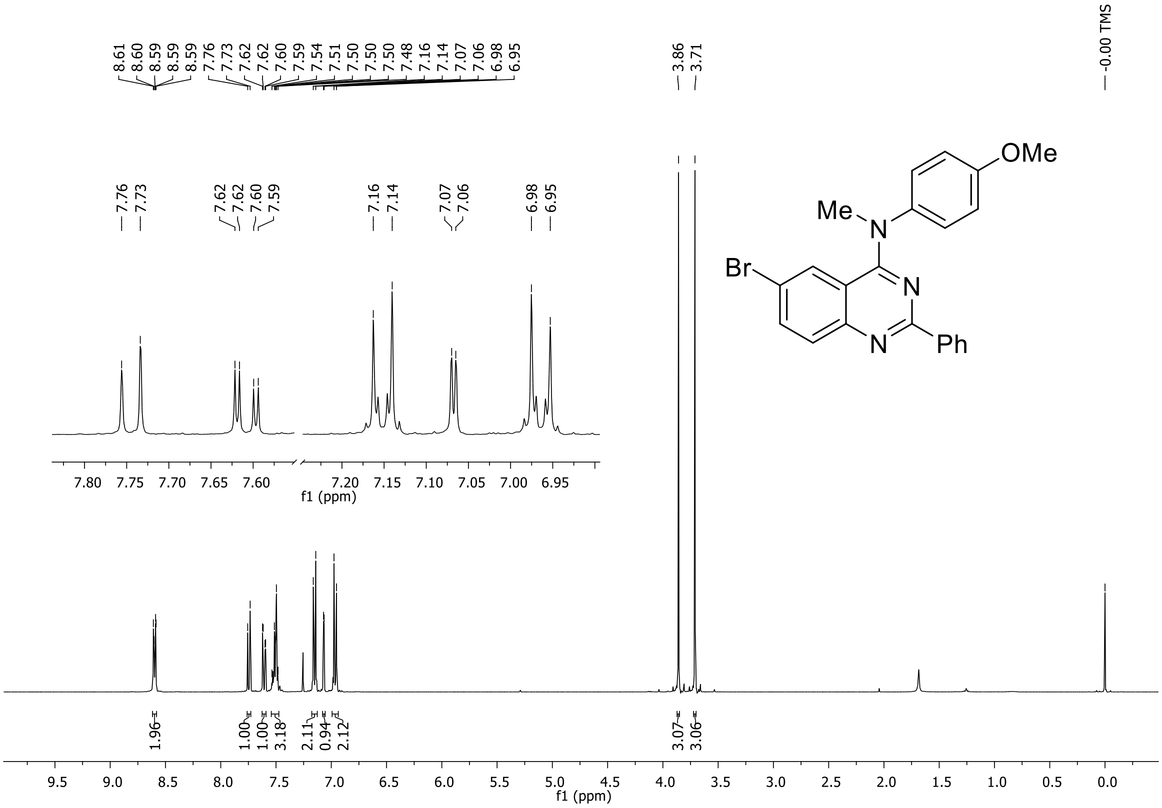

^{13}C NMR (100 MHz, $CDCl_3$, ppm) of 6-Bromo-*N*-(4-methoxyphenyl)-*N*-methyl-2-phenylquinazolin-4-amine (**10b**)

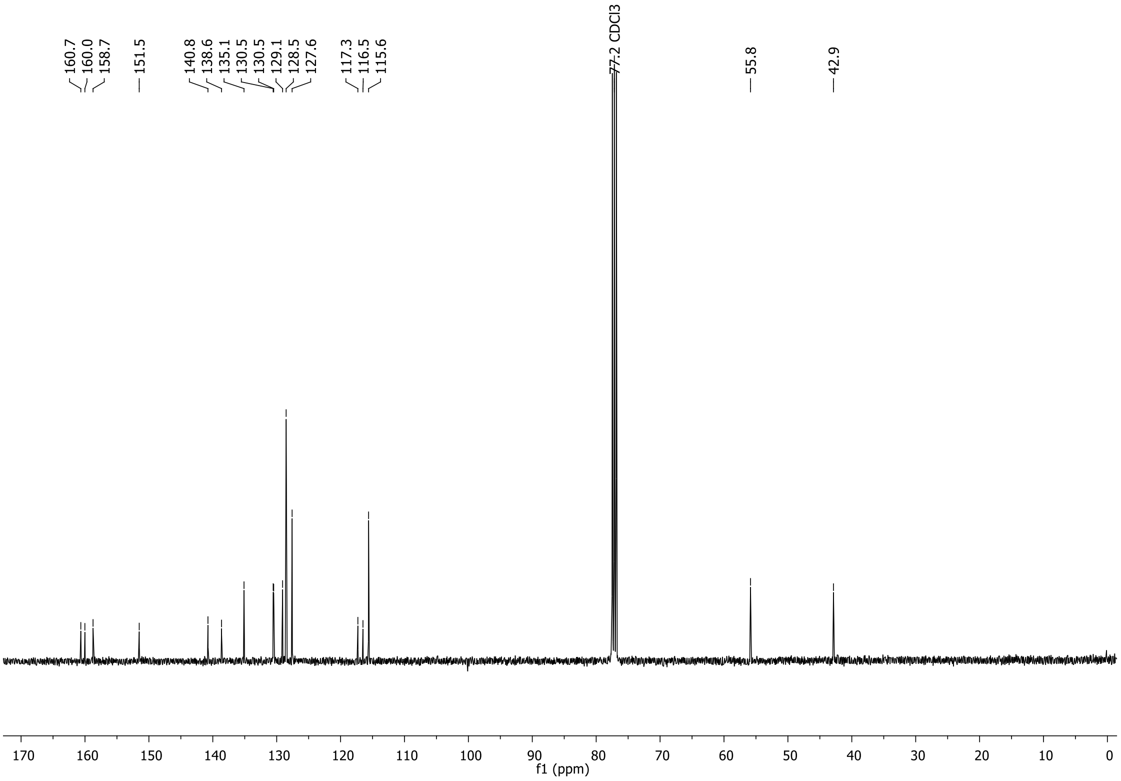

^{1}H NMR (400 MHz, $CDCl_3$, ppm) of 6-iodo-*N*-(3-methoxyphenyl)-*N*-methyl-2-phenylquinazolin-4-amine (**10c**)

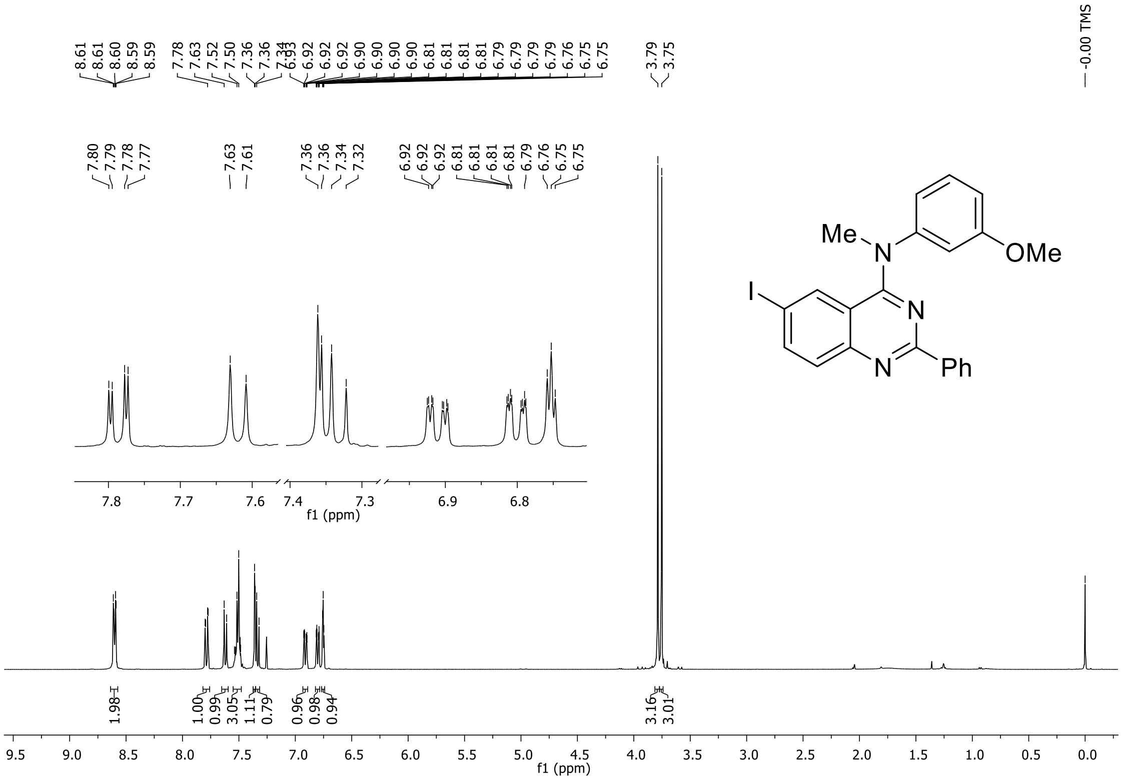

^{13}C NMR (100 MHz, $CDCl_3$, ppm) of 6-iodo-*N*-(3-methoxyphenyl)-*N*-methyl-2-phenylquinazolin-4-amine (**10c**)

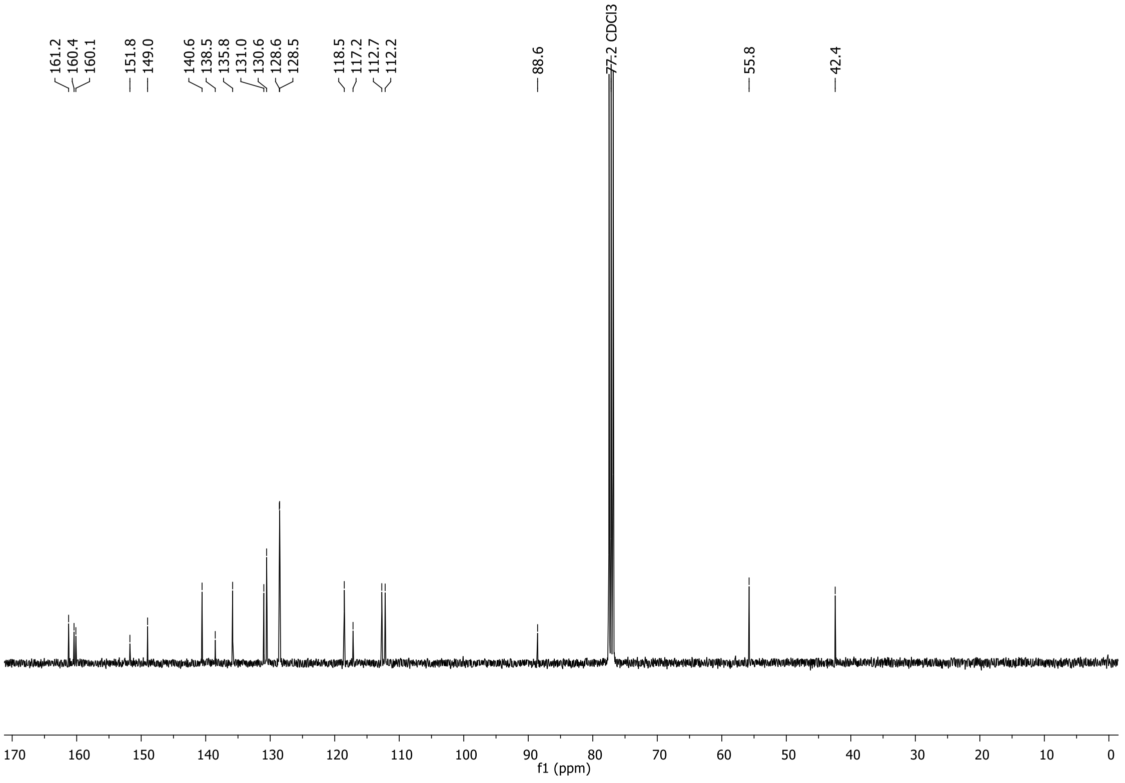

^{1}H NMR (400 MHz, $CDCl_3$, ppm) of 6-Bromo-*N*-(3-methoxyphenyl)-*N*-methyl-2-phenylquinazolin-4-amine (**10d**)

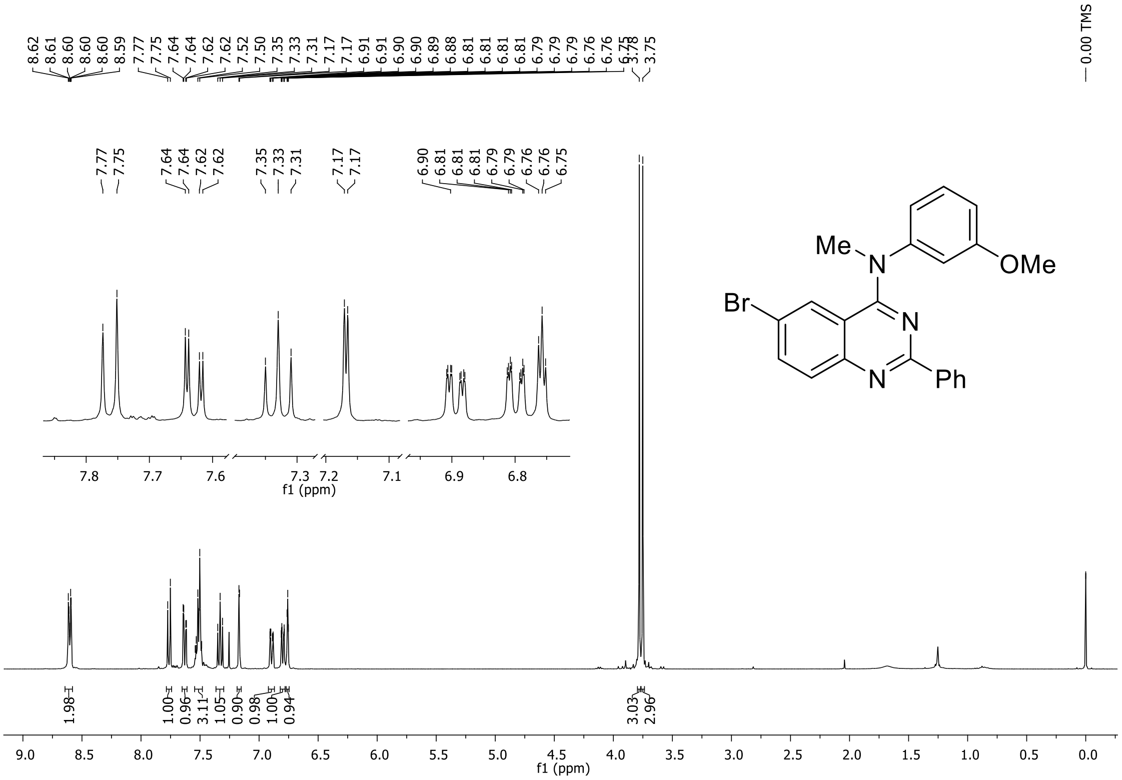

^{13}C NMR (100 MHz, $CDCl_3$, ppm) of 6-Bromo-*N*-(3-methoxyphenyl)-*N*-methyl-2-phenylquinazolin-4-amine (**10d**)

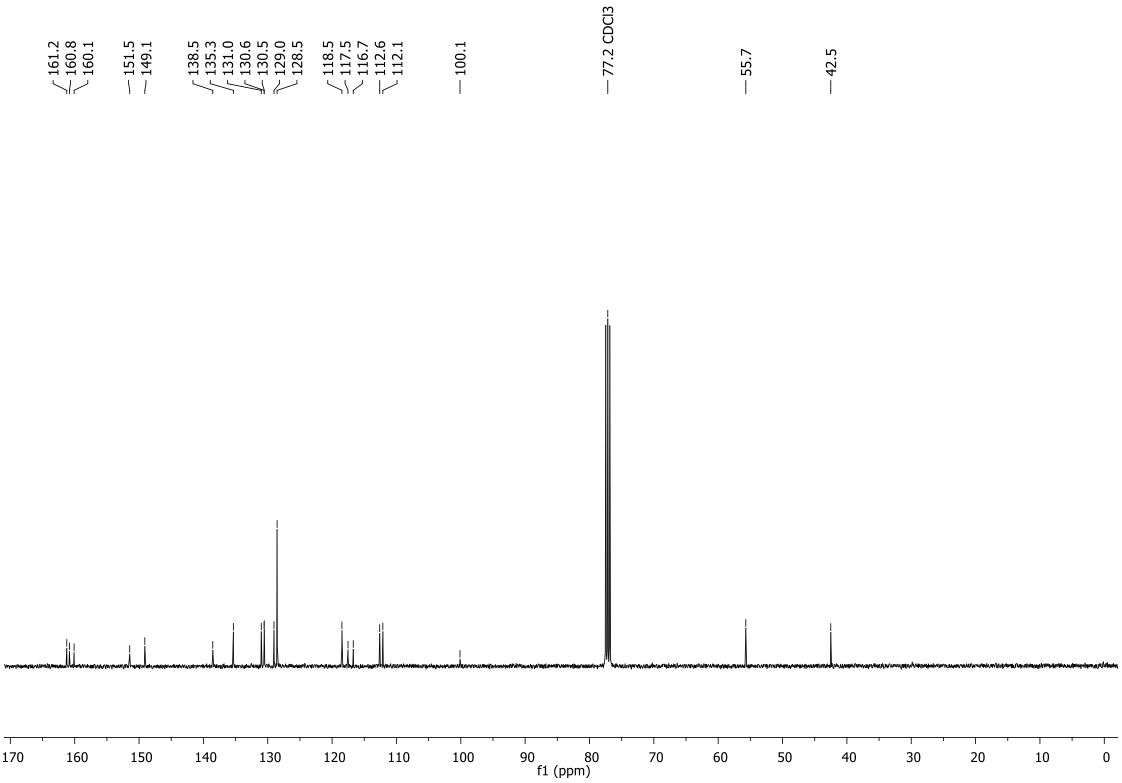

^{1}H NMR (400 MHz, $CDCl_3$, ppm) of 6-iodo-*N*-(2-methoxyphenyl)-*N*-methyl-2-phenylquinazolin-4-amine (**10e**)

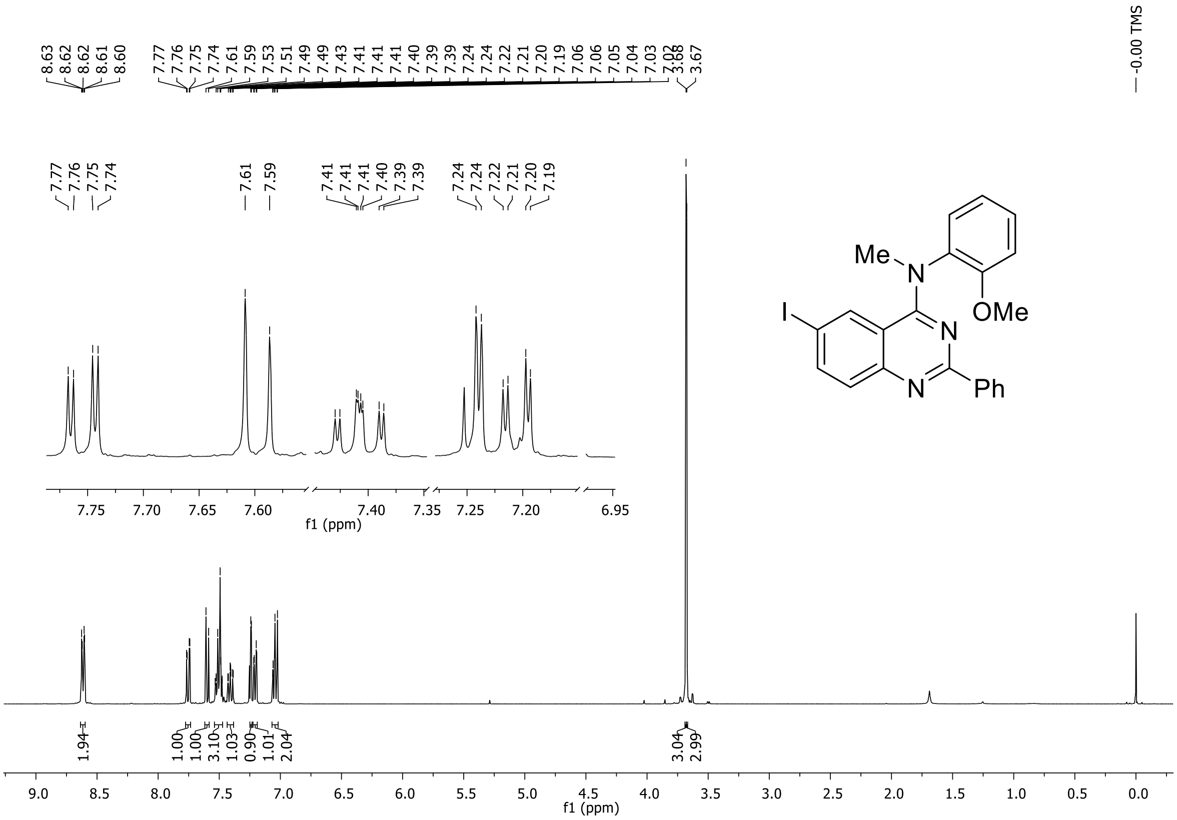

^{13}C NMR (100 MHz, $CDCl_3$, ppm) of 6-iodo-*N*-(2-methoxyphenyl)-*N*-methyl-2-phenylquinazolin-4-amine (**10e**)

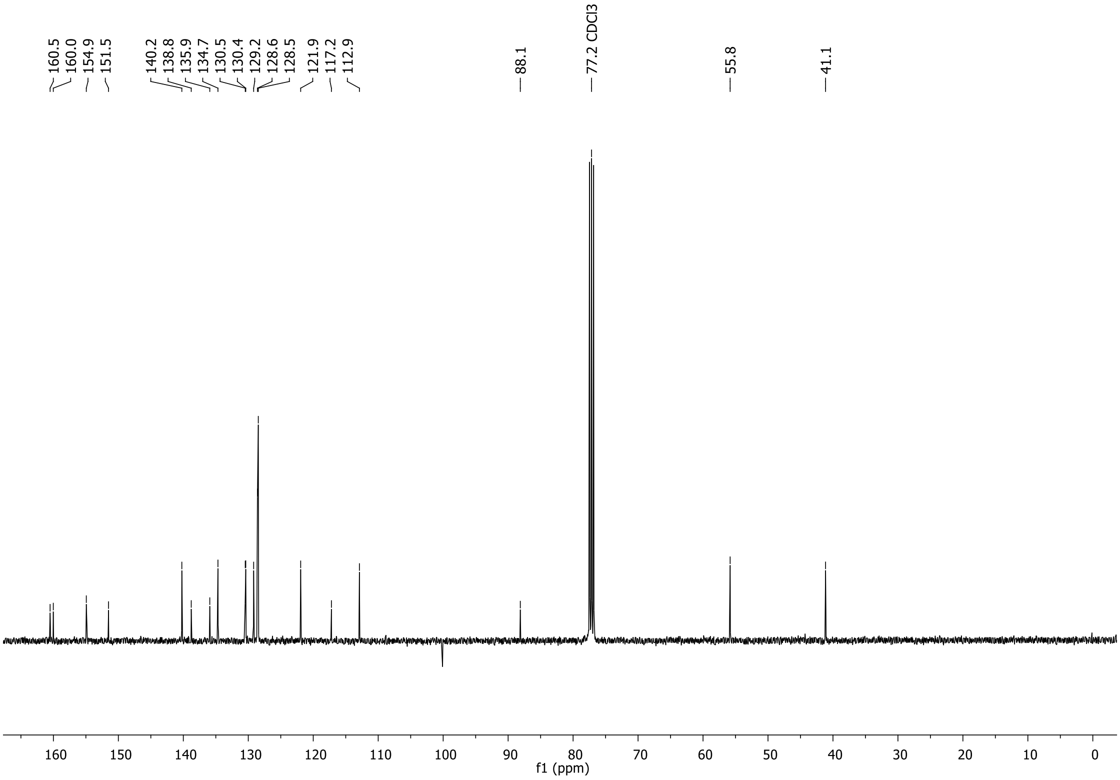

^1H NMR (400 MHz, $CDCl_3$, ppm) of 6-Bromo-*N*-(2-methoxyphenyl)-*N*-methyl-2-phenylquinazolin-4-amine (**10f**)

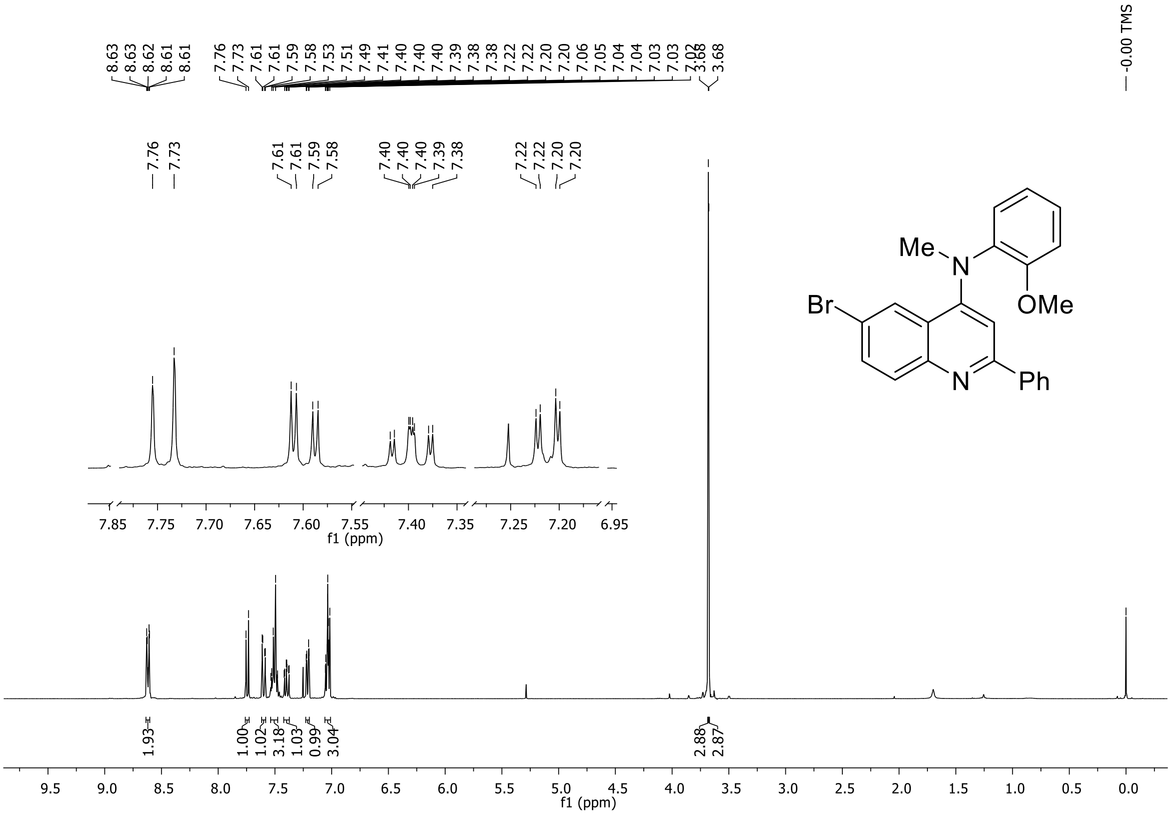

^{13}C NMR (100 MHz, $CDCl_3$, ppm) of 6-Bromo-*N*-(2-methoxyphenyl)-*N*-methyl-2-phenylquinazolin-4-amine (**10f**)

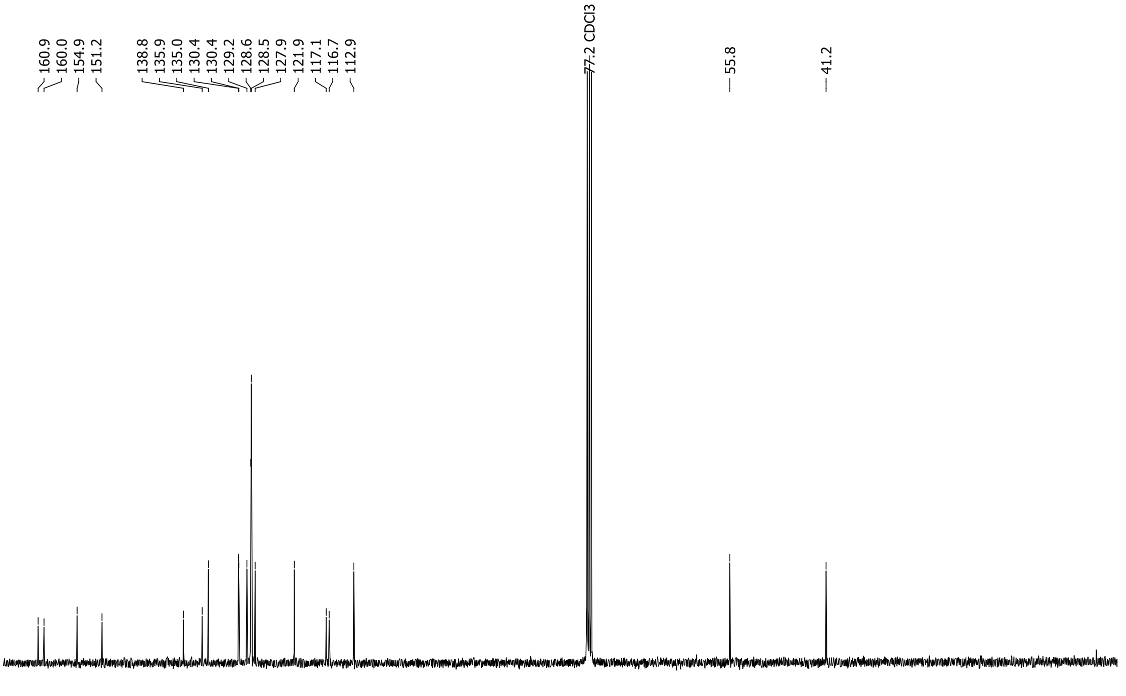

^1H NMR (400 MHz, $CDCl_3$, ppm) of 6-iodo-*N*-methyl-2-phenyl-*N*-(3-tolyl)quinazolin-4-amine (**10i**)

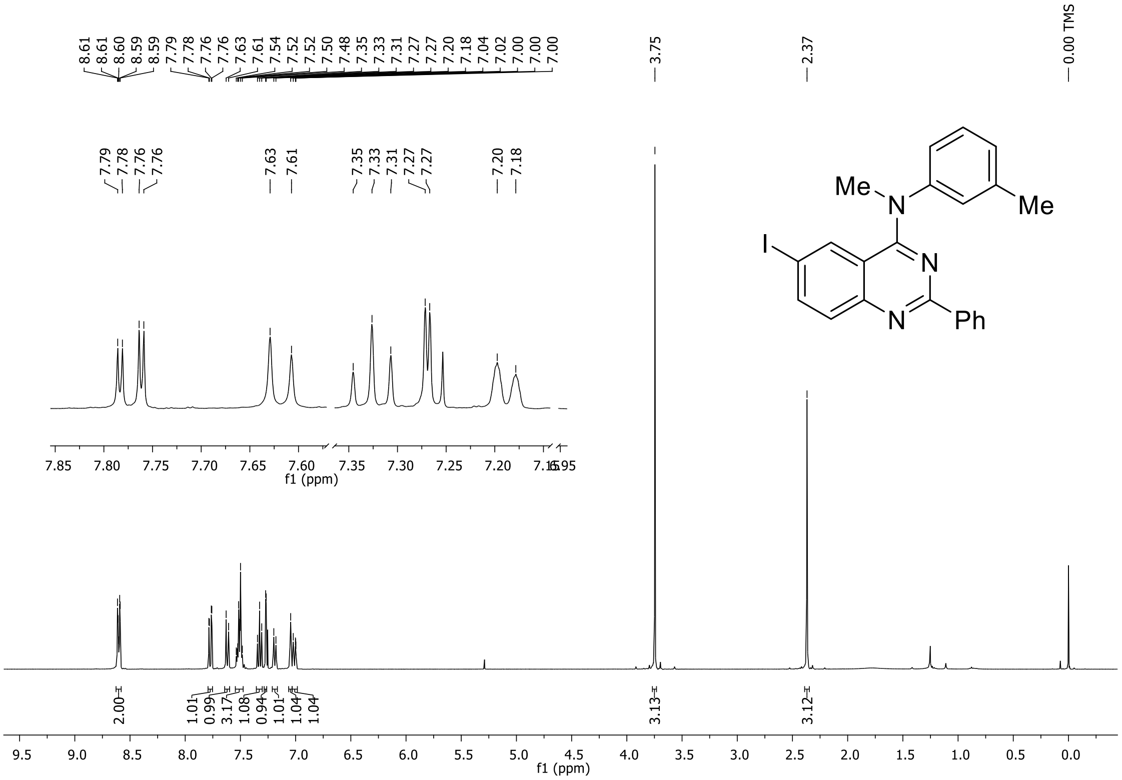

^{13}C NMR (100 MHz, $CDCl_3$, ppm) of 6-iodo-*N*-methyl-2-phenyl-*N*-(3-tolyl)quinazolin-4-amine (**10i**)

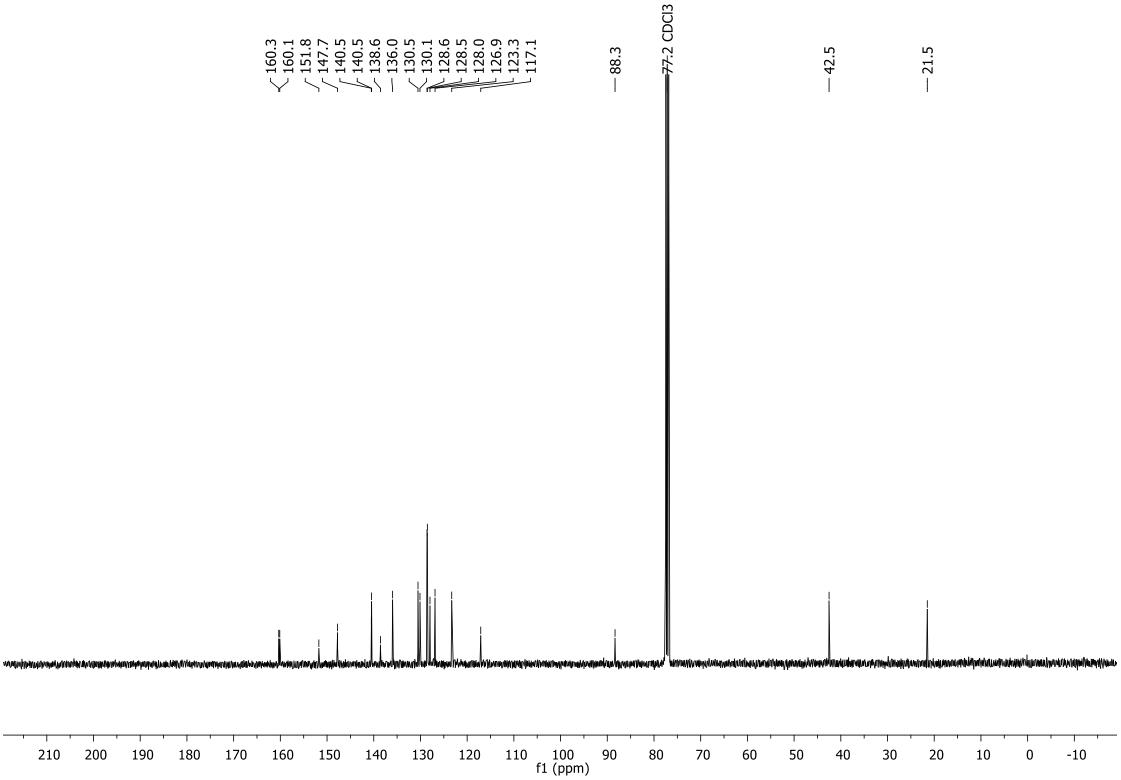

^{1}H NMR (400 MHz, $CDCl_3$, ppm) of 6-Bromo-*N*-methyl-2-phenyl-*N*-(3-tolyl)quinazolin-4-amine (**10j**)

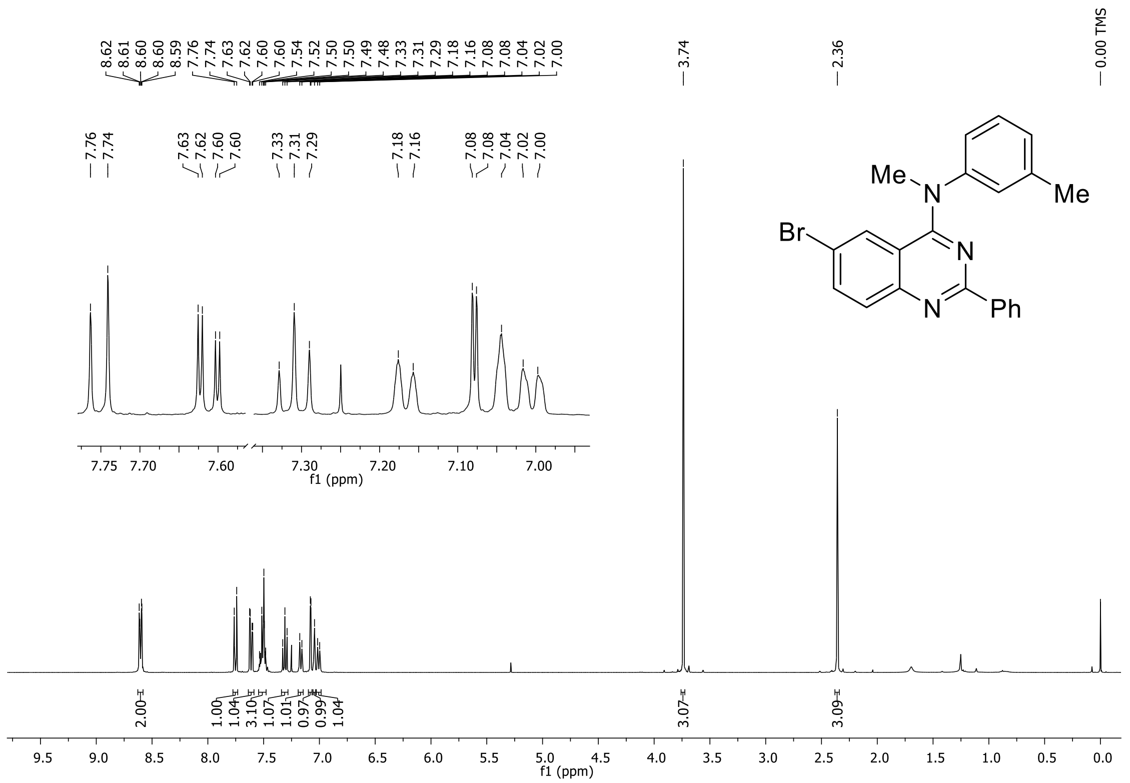

^{13}C NMR (100 MHz, $CDCl_3$, ppm) of 6-Bromo-*N*-methyl-2-phenyl-*N*-(3-tolyl)quinazolin-4-amine (**10j**)

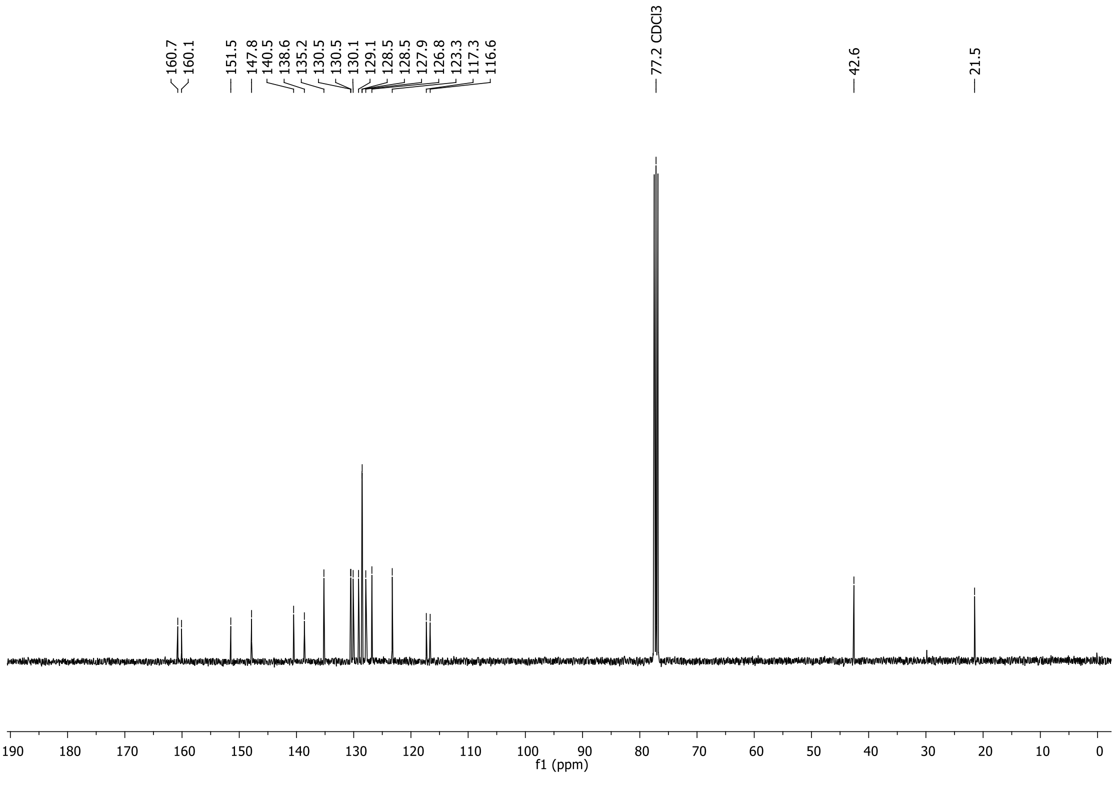

^{1}H NMR (400 MHz, $CDCl_3$, ppm) of *N*-(3-Bromophenyl)-6-iodo-*N*-methyl-2-phenylquinazolin-4-amine (**10k**)

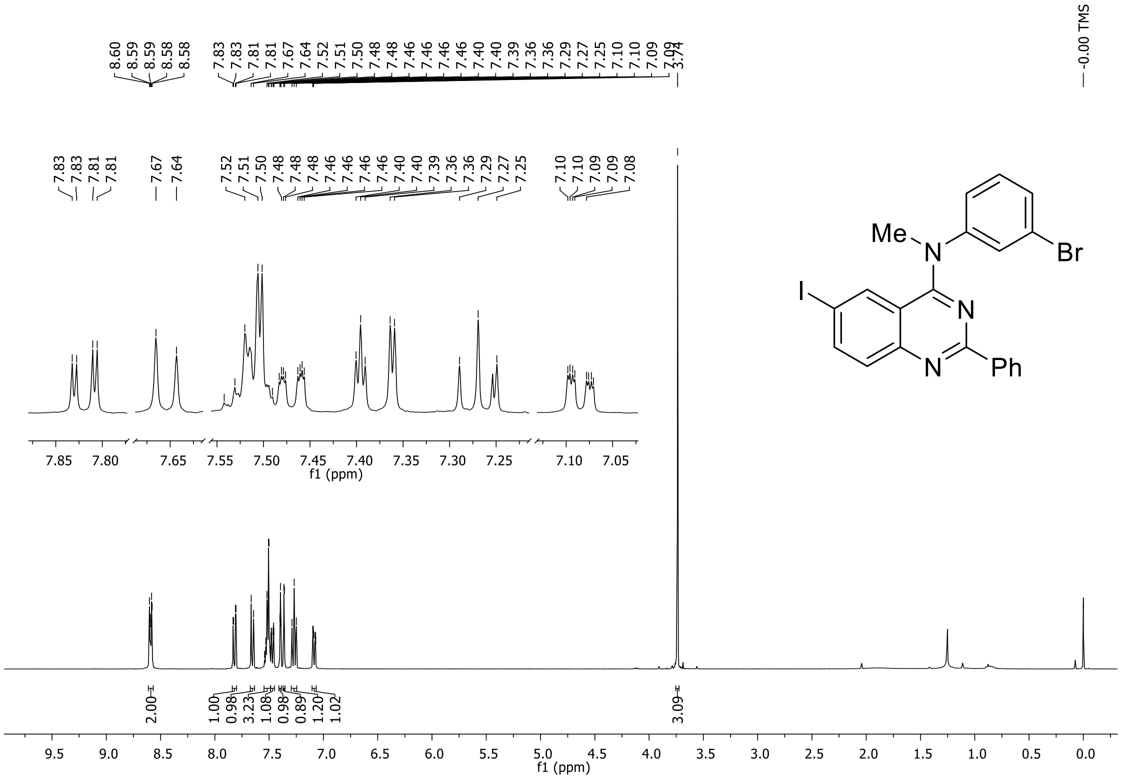

^{13}C NMR (100 MHz, $CDCl_3$, ppm) of *N*-(3-Bromophenyl)-6-iodo-*N*-methyl-2-phenylquinazolin-4-amine (**10k**)

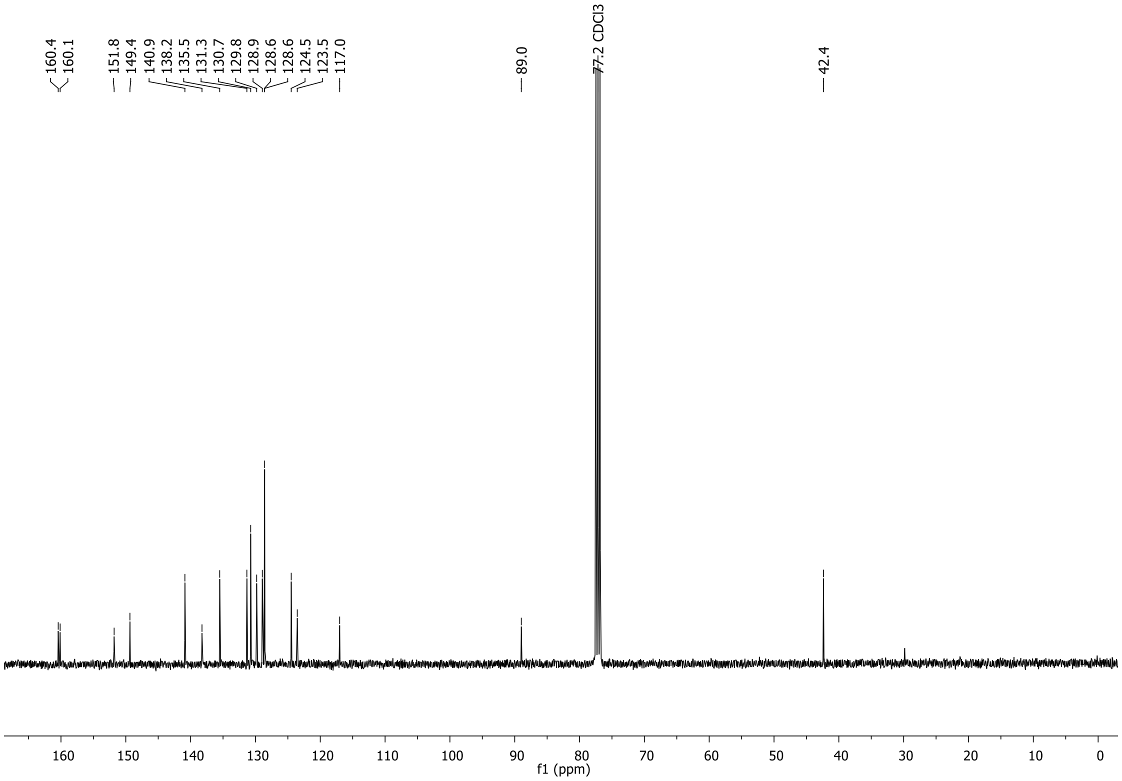

^{1}H NMR (400 MHz, CDCl$_3$, ppm) of 6-Bromo-*N*-(3-bromophenyl)-*N*-metil-2-phenylquinazolin-4-amine (**10l**)

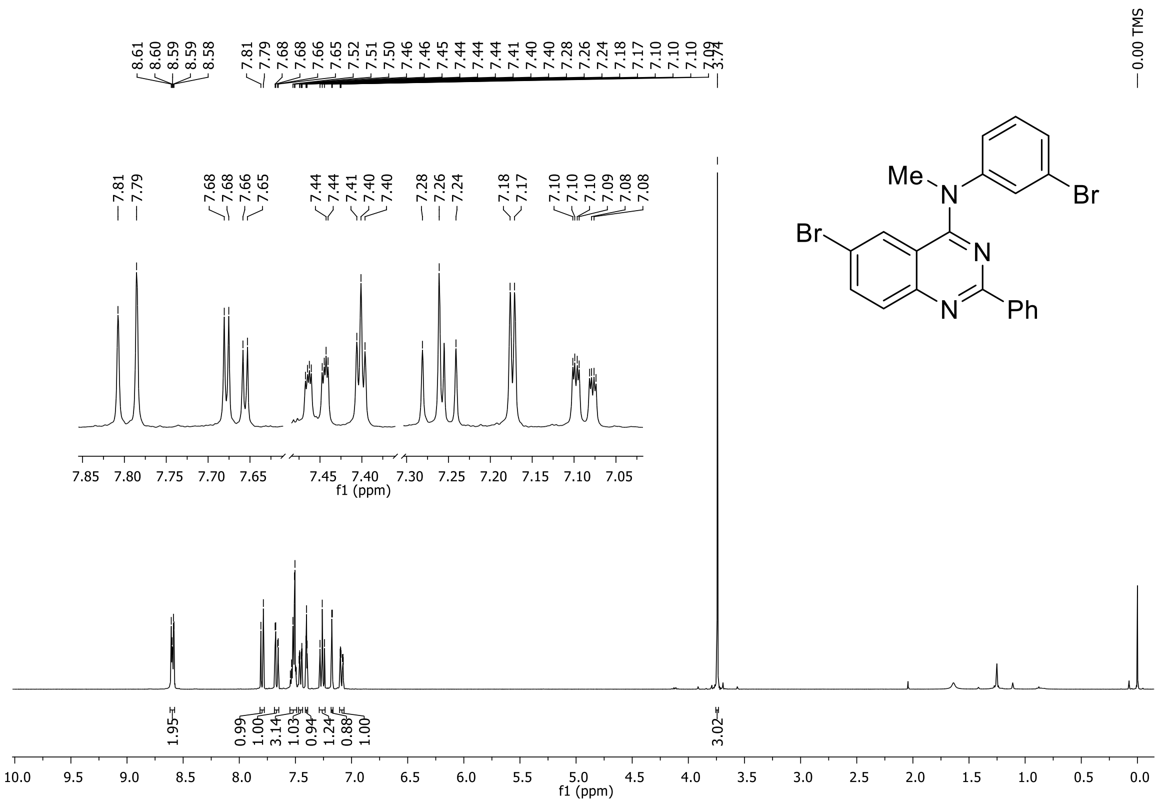

^{13}C NMR (100 MHz, CDCl$_3$, ppm) of 6-Bromo-*N*-(3-bromophenyl)-*N*-metil-2-phenylquinazolin-4-amine (**10l**)

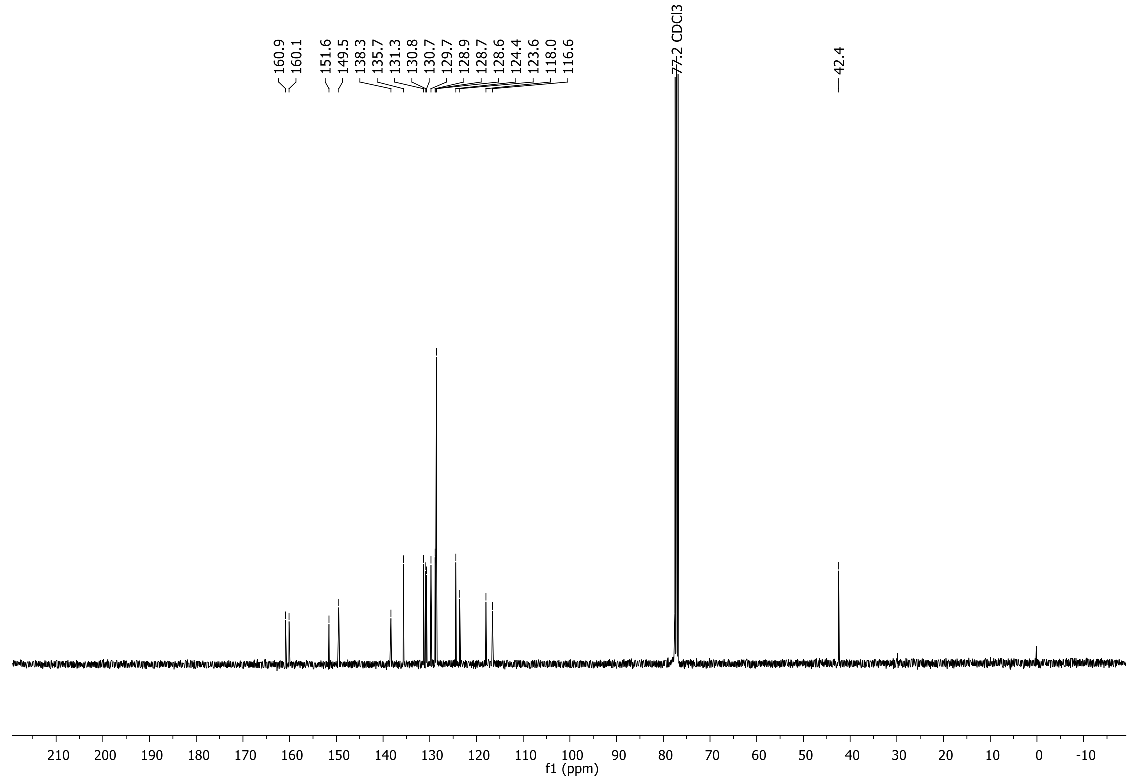

^{1}H NMR (400 MHz, $CDCl_3$, ppm) of *N*-(4-fluorophenyl)-6-iodo-*N*-methyl-2-phenylquinazolin-4-amine (**10m**)

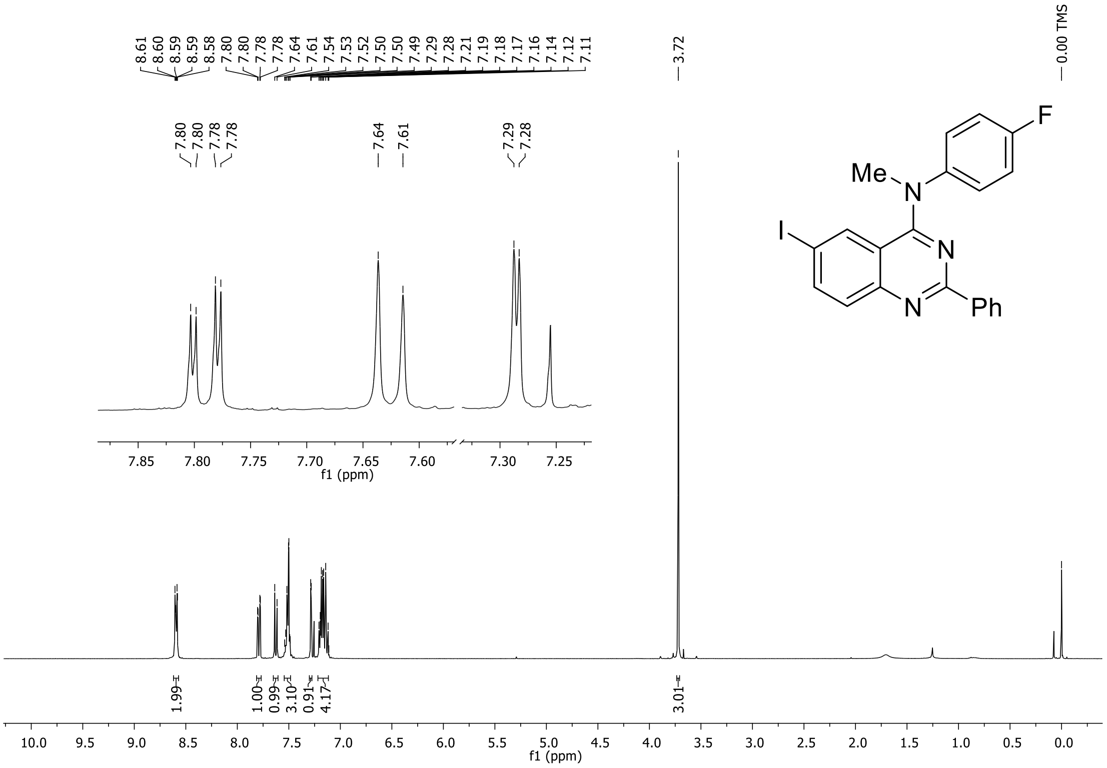

^{13}C NMR (100 MHz, $CDCl_3$, ppm) of *N*-(4-fluorophenyl)-6-iodo-*N*-methyl-2-phenylquinazolin-4-amine (**10m**)

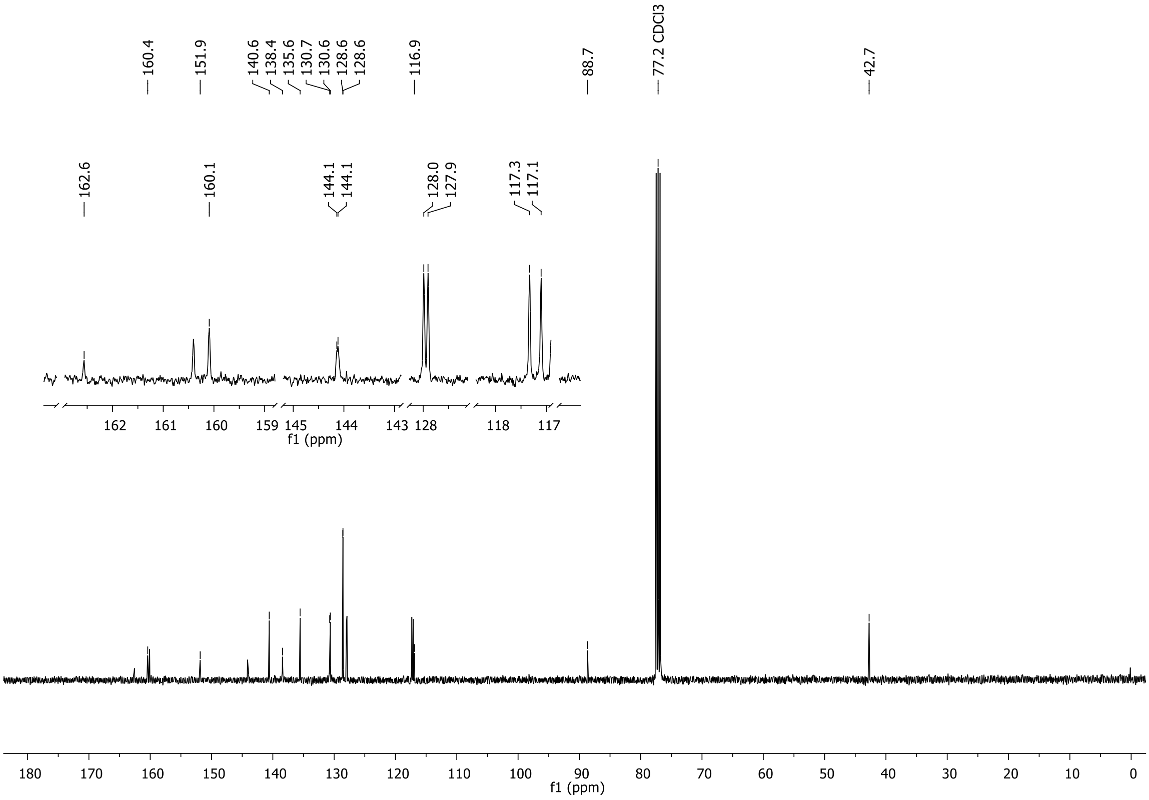

^{1}H NMR (400 MHz, $CDCl_3$, ppm) of 6-Bromo-*N*-(4-fluorophenyl)-*N*-methyl-2-phenylquinazolin-4-amine (**10n**)

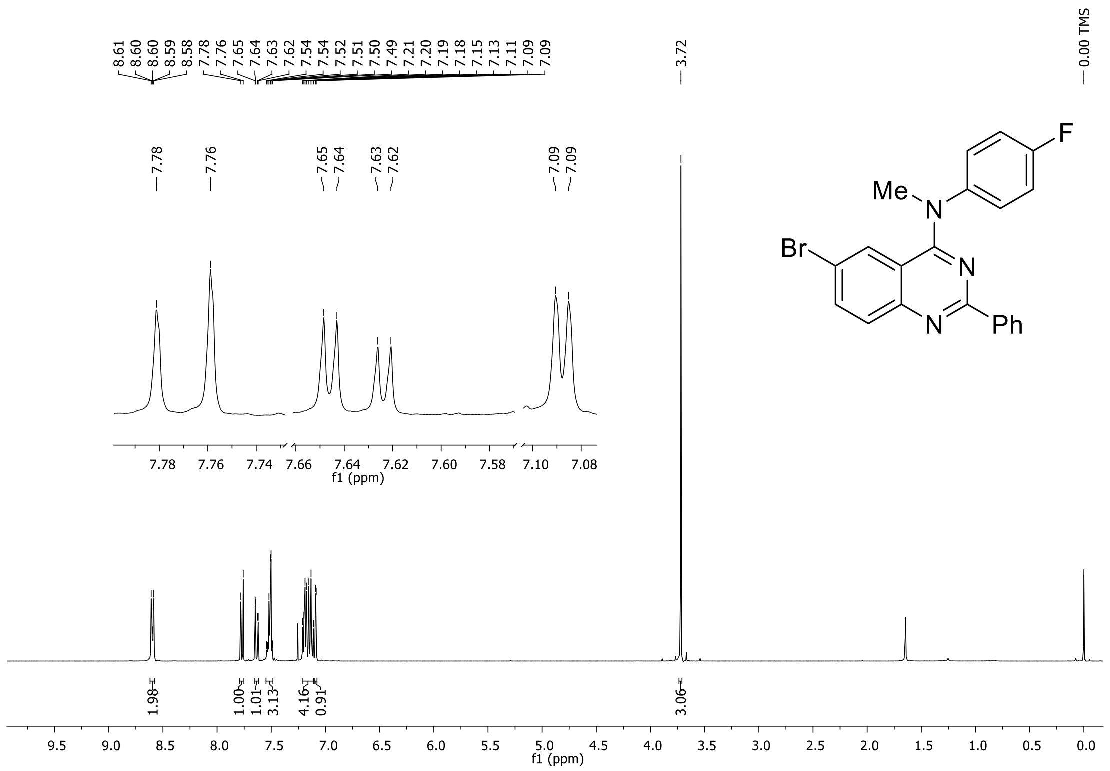

^{13}C NMR (100 MHz, $CDCl_3$, ppm) of 6-Bromo-*N*-(4-fluorophenyl)-*N*-methyl-2-phenylquinazolin-4-amine (**10n**)

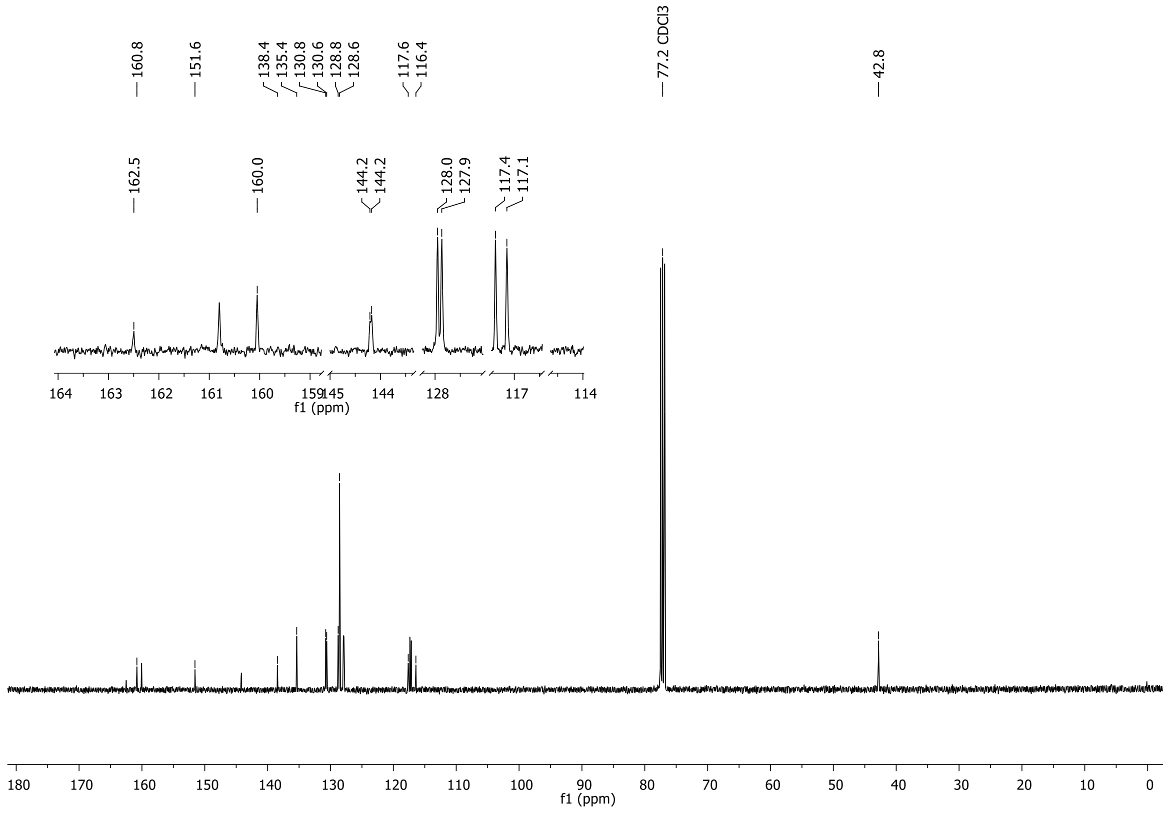

^{1}H NMR (400 MHz, $CDCl_3$, ppm) of *N*-(3-fluorophenyl)-6-iodo-*N*-methyl-2-phenylquinazolin-4-amine (**10o**)

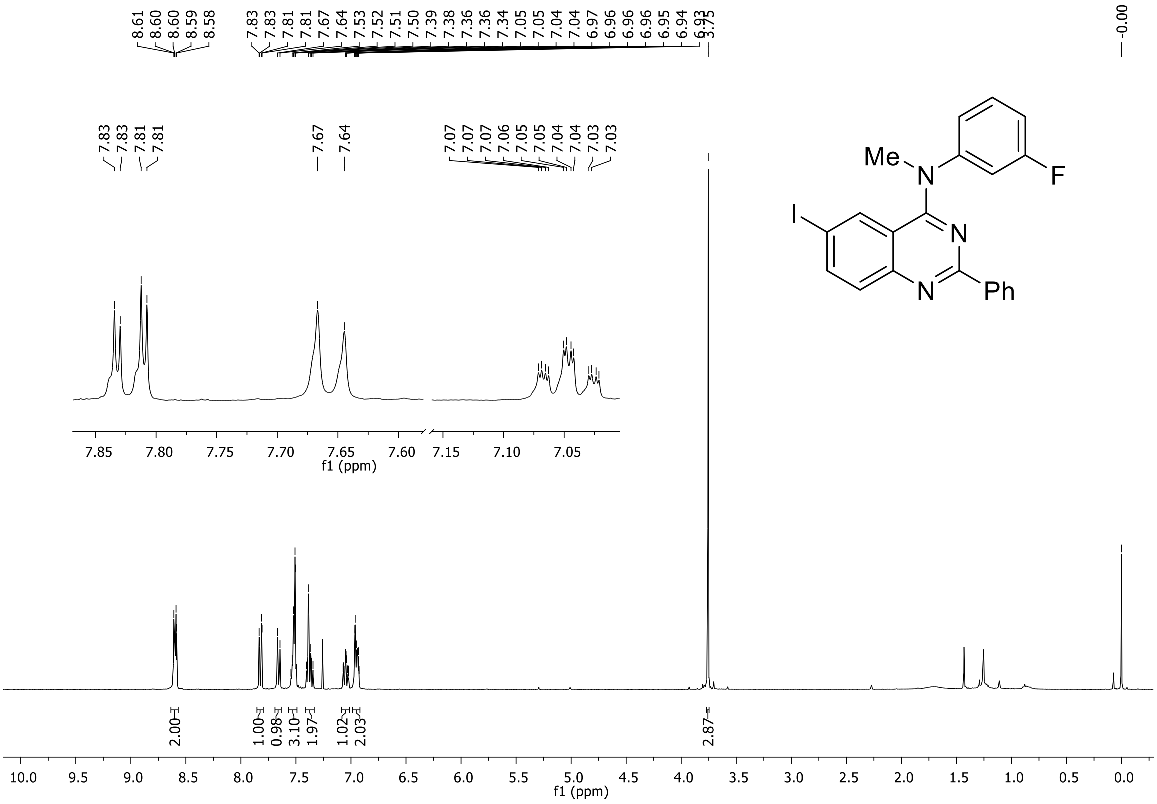

^{13}C NMR (100 MHz, $CDCl_3$, ppm) of *N*-(3-fluorophenyl)-6-iodo-*N*-methyl-2-phenylquinazolin-4-amine (**10o**)

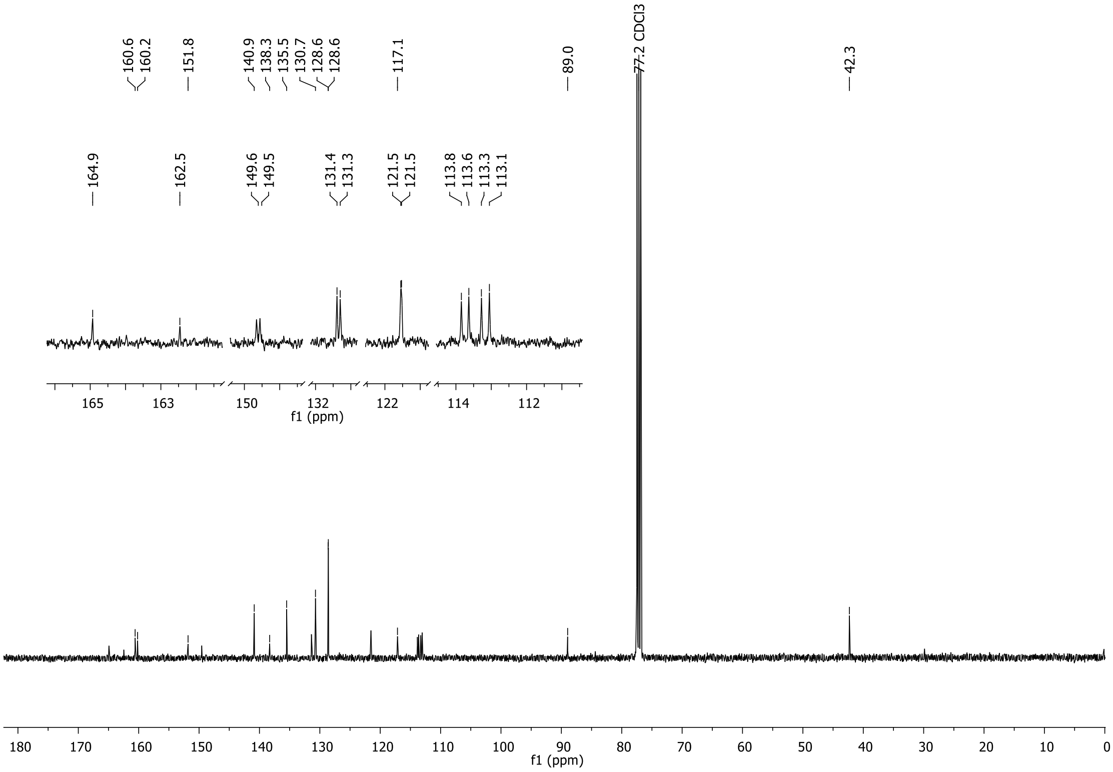

^{1}H NMR (400 MHz, $CDCl_3$, ppm) of 6-Bromo-*N*-(3-fluorophenyl)-*N*-methyl-2-phenylquinazolin-4-amine (**10p**)

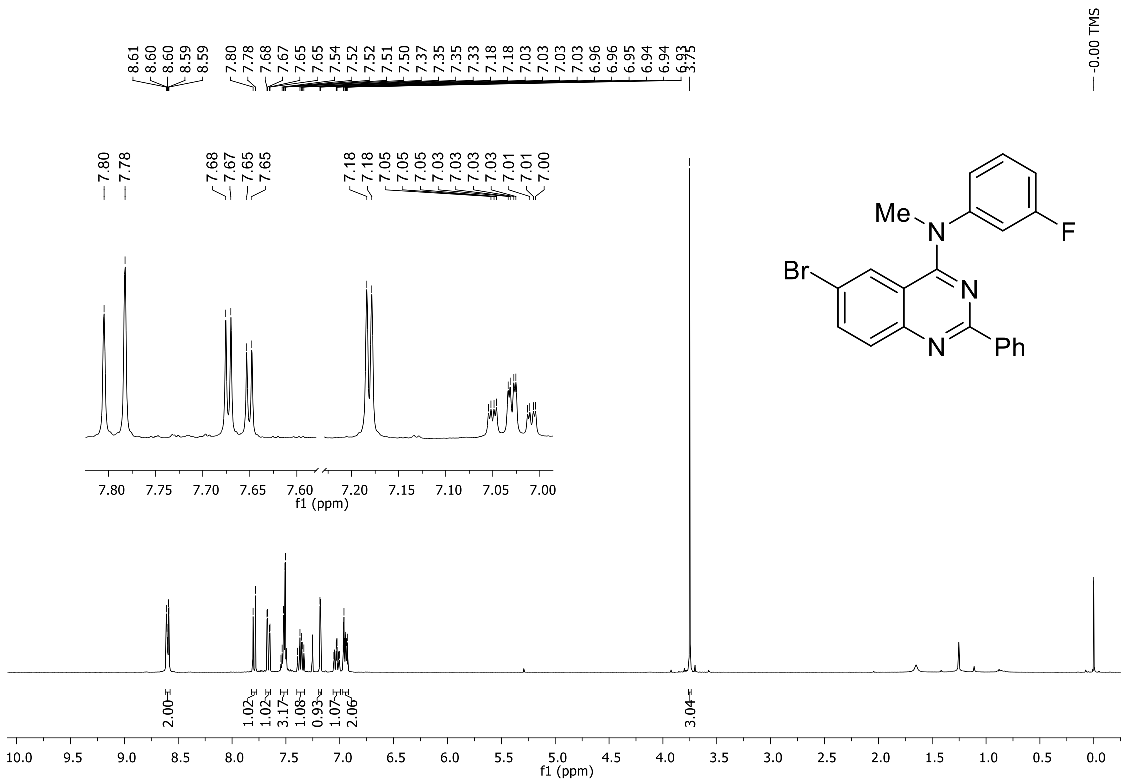

^{13}C NMR (100 MHz, $CDCl_3$, ppm) of 6-Bromo-*N*-(3-fluorophenyl)-*N*-methyl-2-phenylquinazolin-4-amine (**10p**)

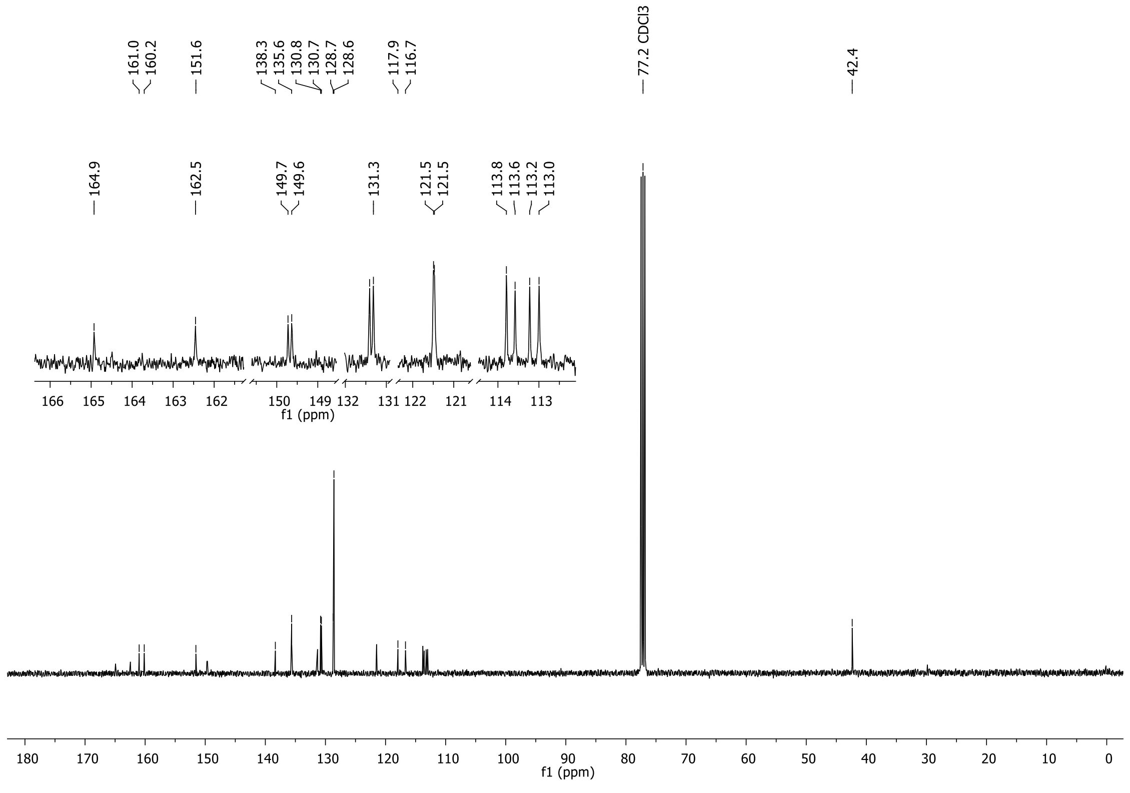

^{1}H NMR (400 MHz, $CDCl_3$, ppm) of 6-iodo-2-phenyl-*N*-(2-tolyl)quinazolin-4-amine (**15a**)

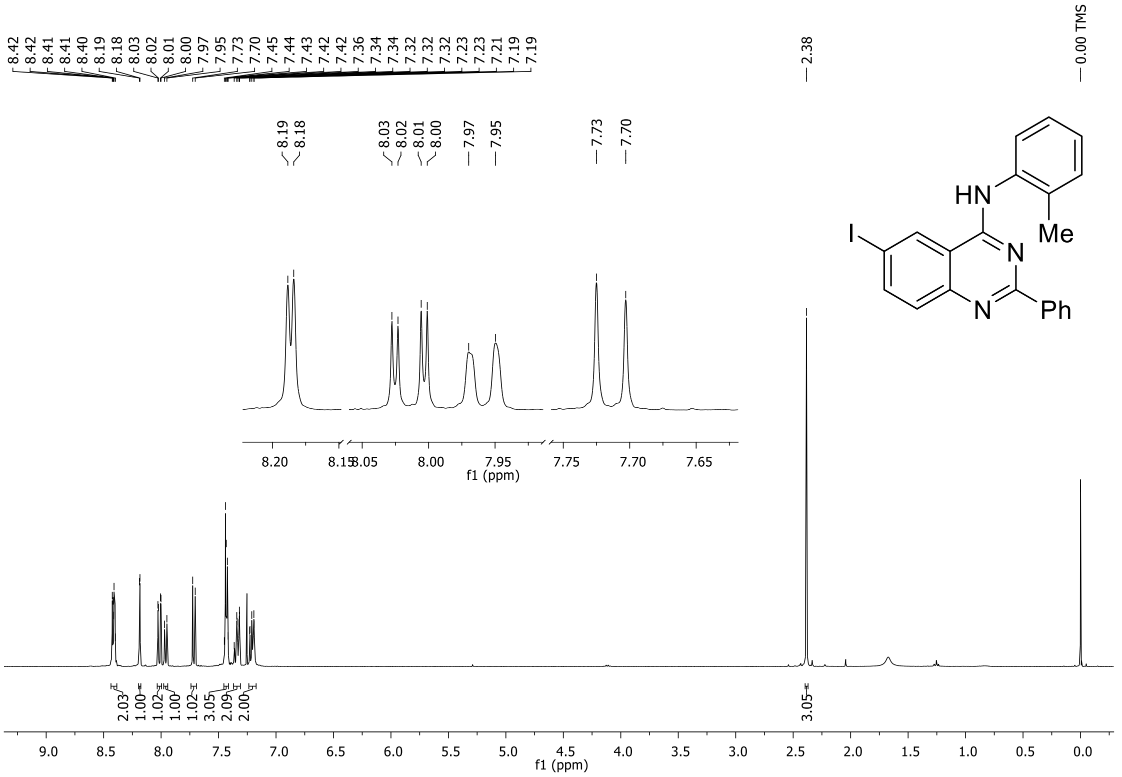

^{13}C NMR (100 MHz, $CDCl_3$, ppm) of 6-iodo-2-phenyl-*N*-(2-tolyl)quinazolin-4-amine (**15a**)

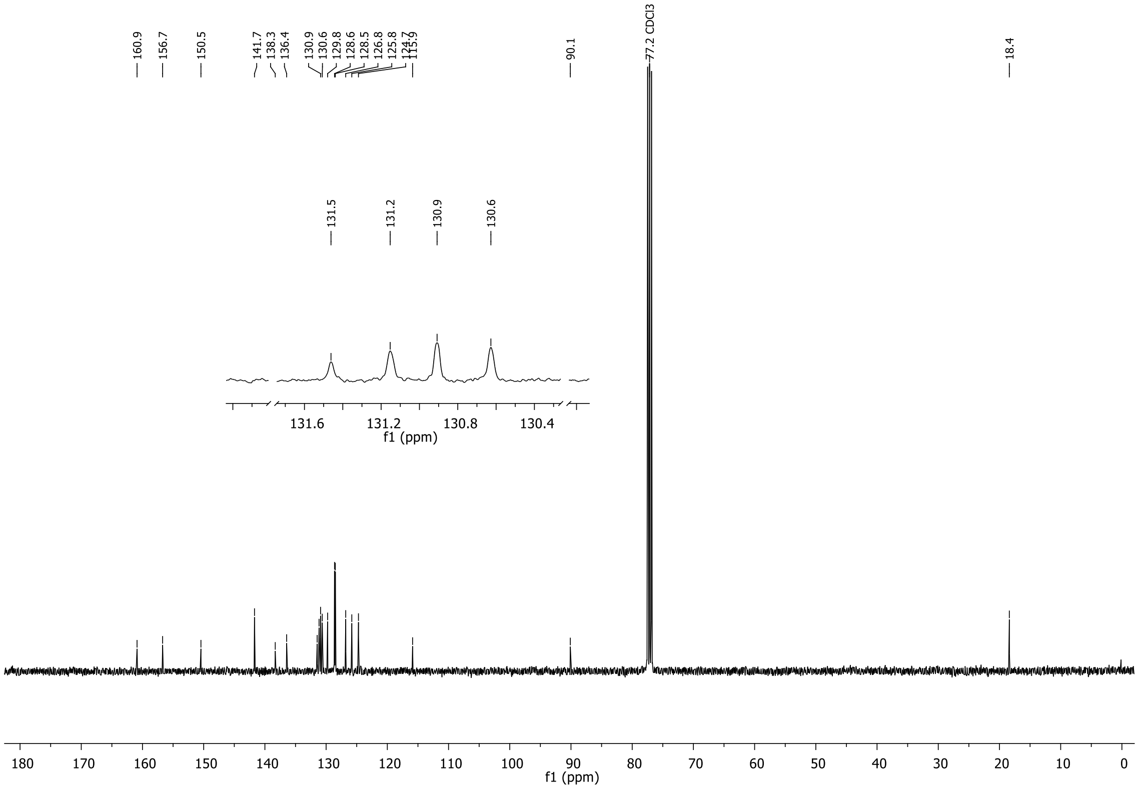

^{1}H NMR (400 MHz, $CDCl_3$, ppm) of 6-Bromo-2-phenyl-*N*-(2-tolyl)quinazolin-4-amine (**15b**)

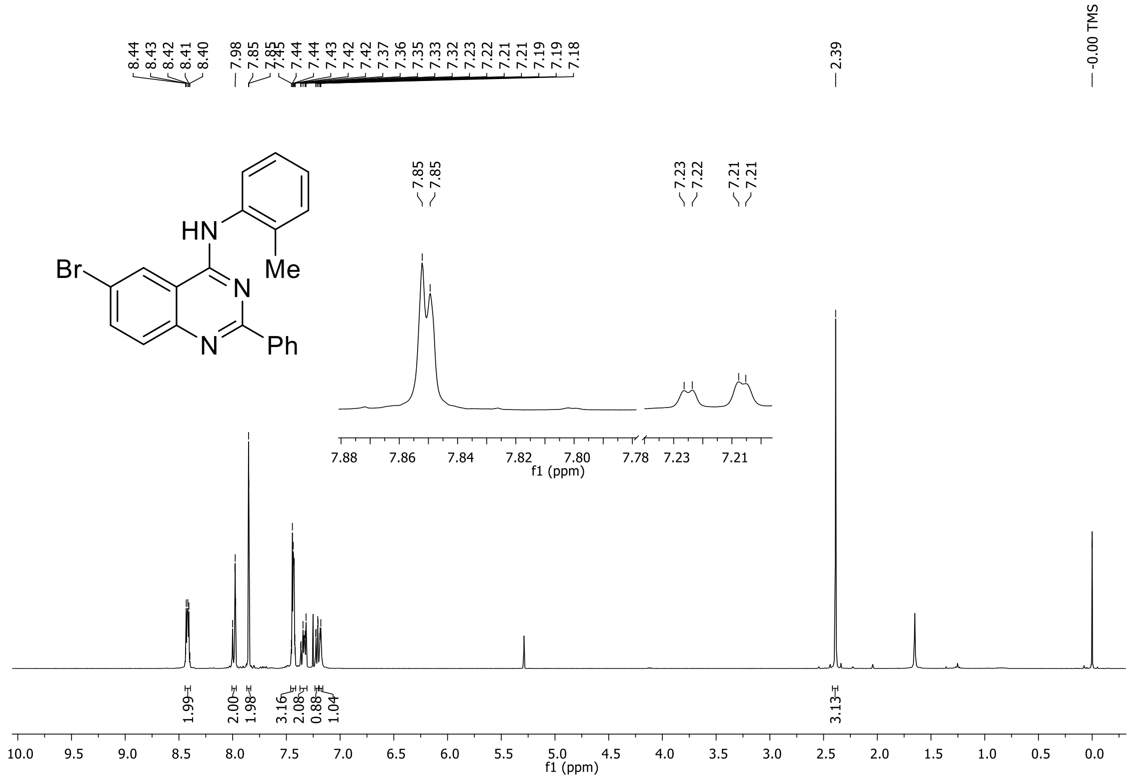

^{13}C NMR (100 MHz, $CDCl_3$, ppm) of 6-Bromo-2-phenyl-*N*-(2-tolyl)quinazolin-4-amine (**15b**)

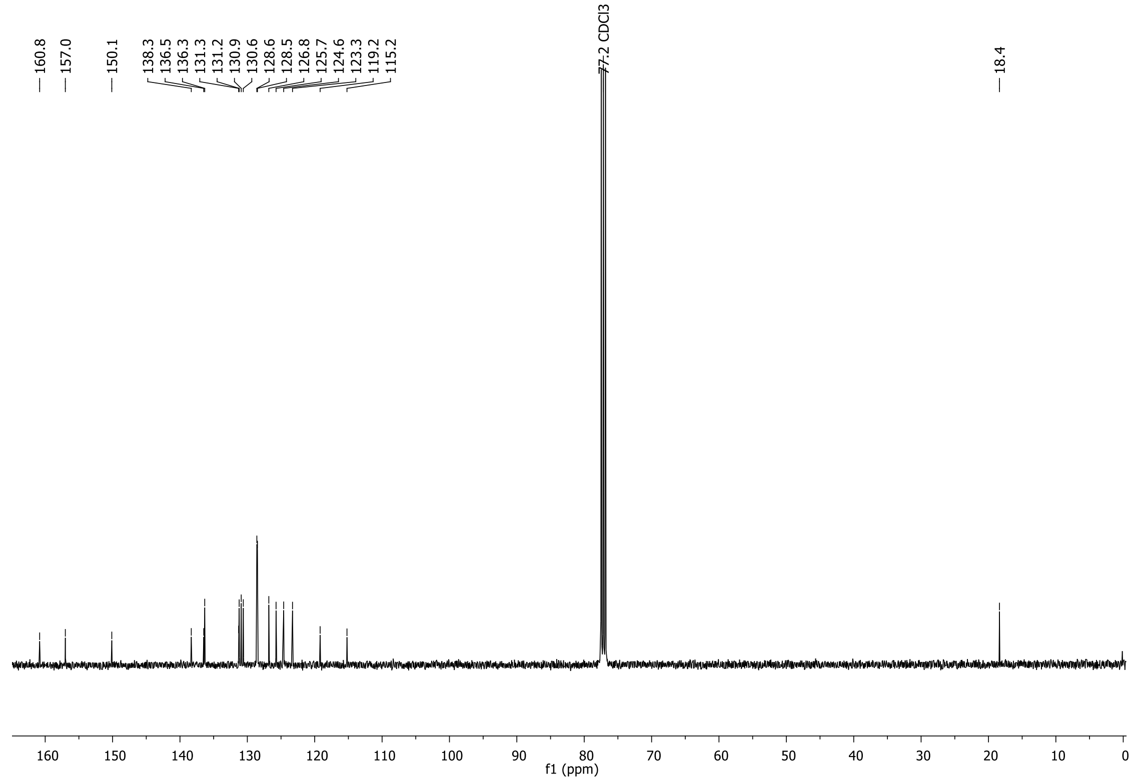

^{1}H NMR (400 MHz, $CDCl_3$, ppm) of *N*-(2-fluorophenyl)-6-iodo-2-phenylquinazolin-4-amine (**15c**)

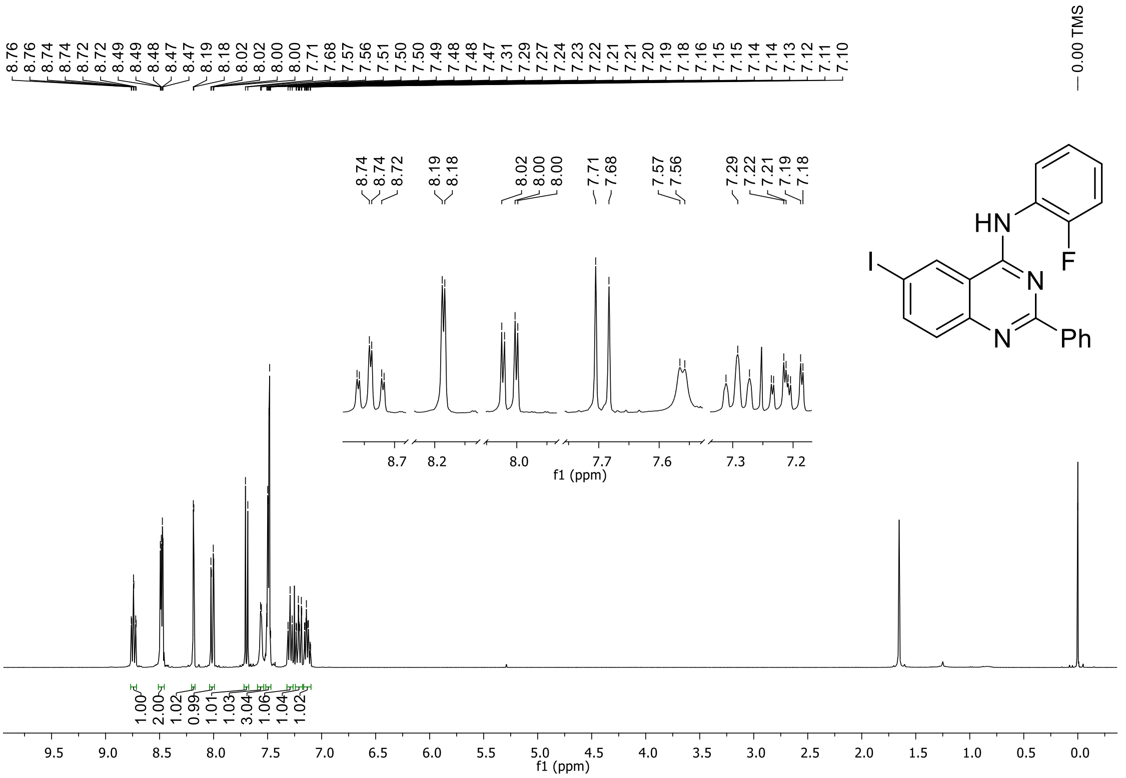

^{13}C NMR (100 MHz, $CDCl_3$, ppm) of *N*-(2-fluorophenyl)-6-iodo-2-phenylquinazolin-4-amine (**15c**)

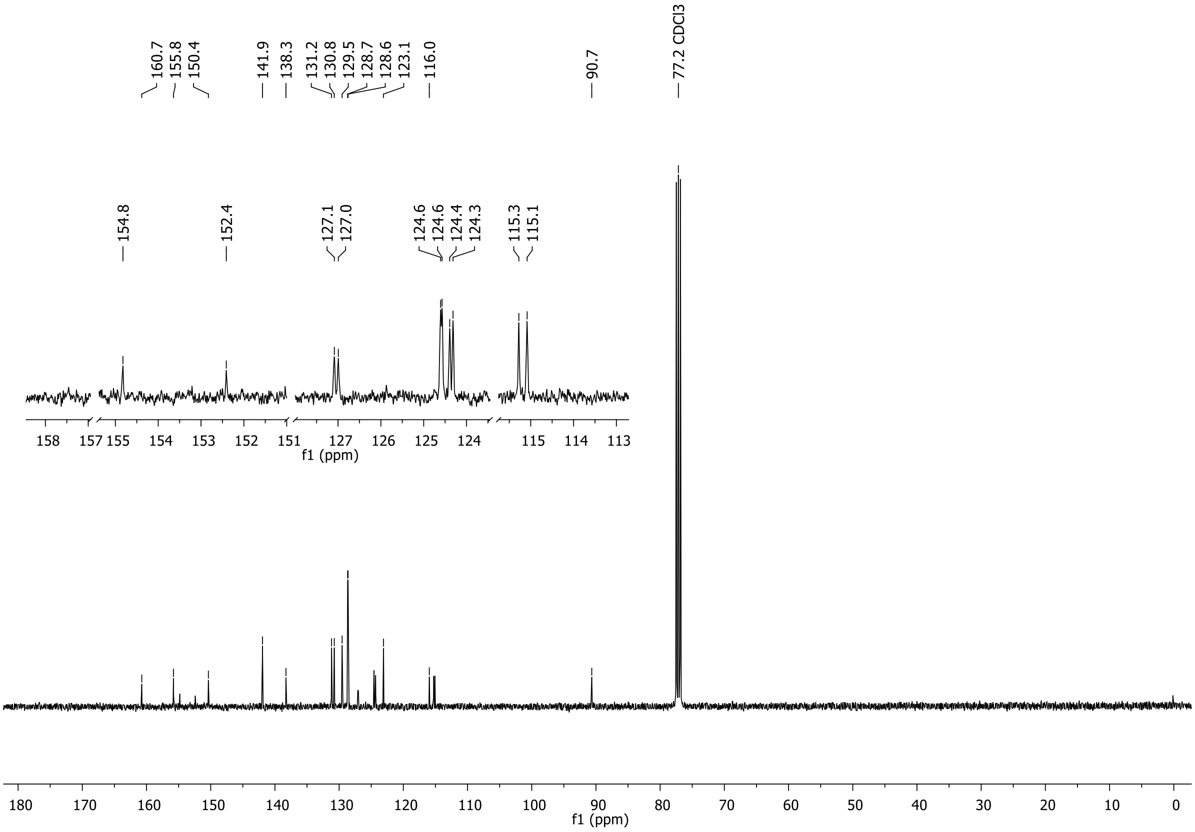

^{1}H NMR (400 MHz, $CDCl_3$, ppm) of 6-Bromo-*N*-(2-fluorophenyl)-2-phenylquinazolin-4-amine (**15d**)

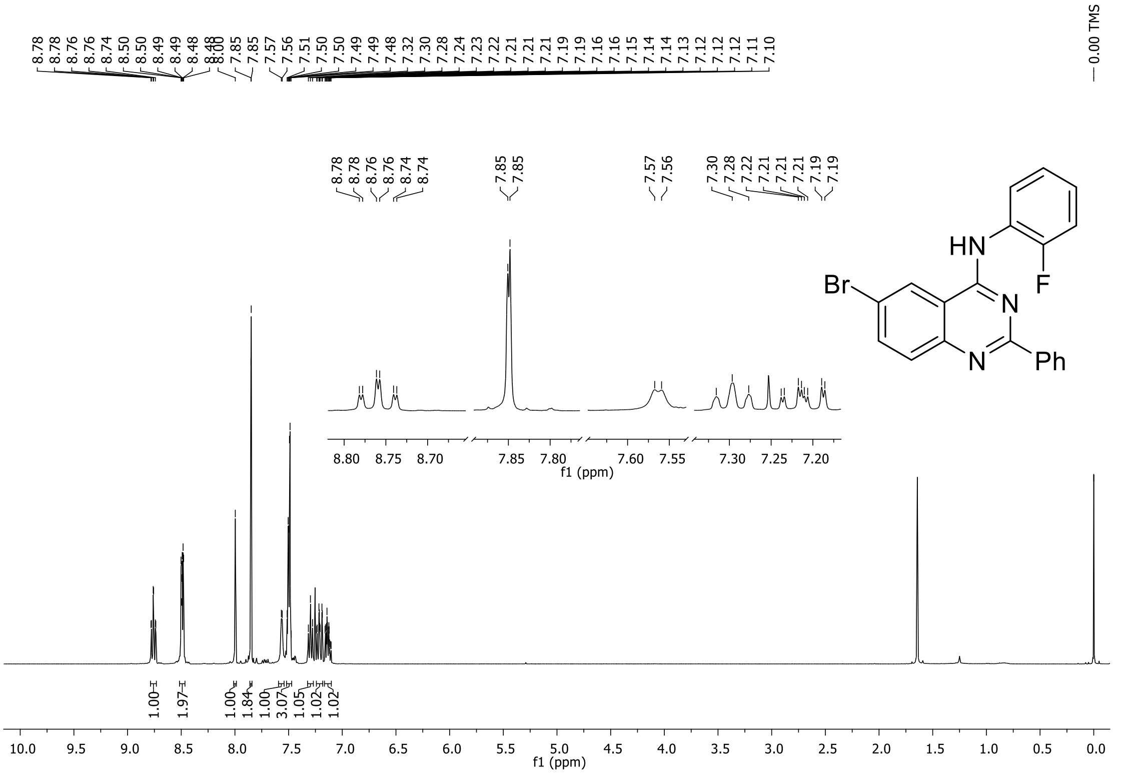

^{13}C NMR (100 MHz, $CDCl_3$, ppm) of 6-Bromo-*N*-(2-fluorophenyl)-2-phenylquinazolin-4-amine (**15d**)

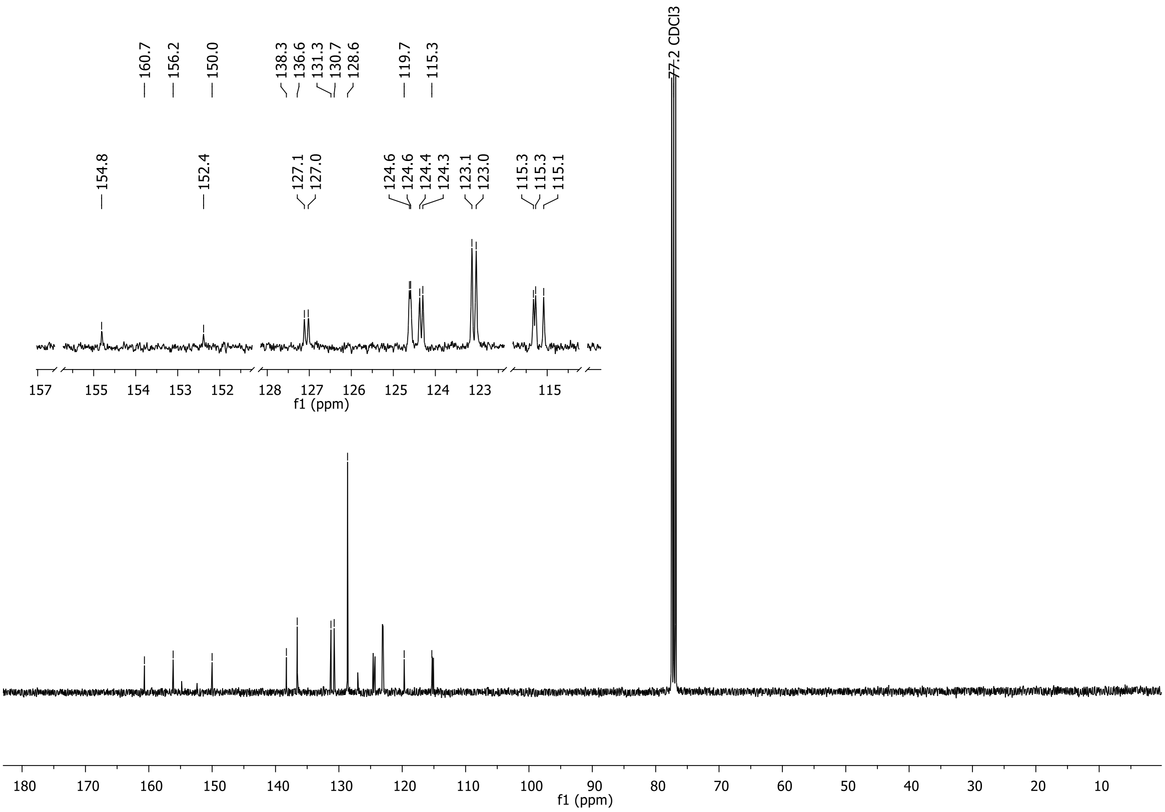

^{1}H NMR (400 MHz, DMSO-d6, ppm) of 6-bromo-*N*-(3-chlorophenyl)-2-phenylquinazolin-4-amine (**15e**)

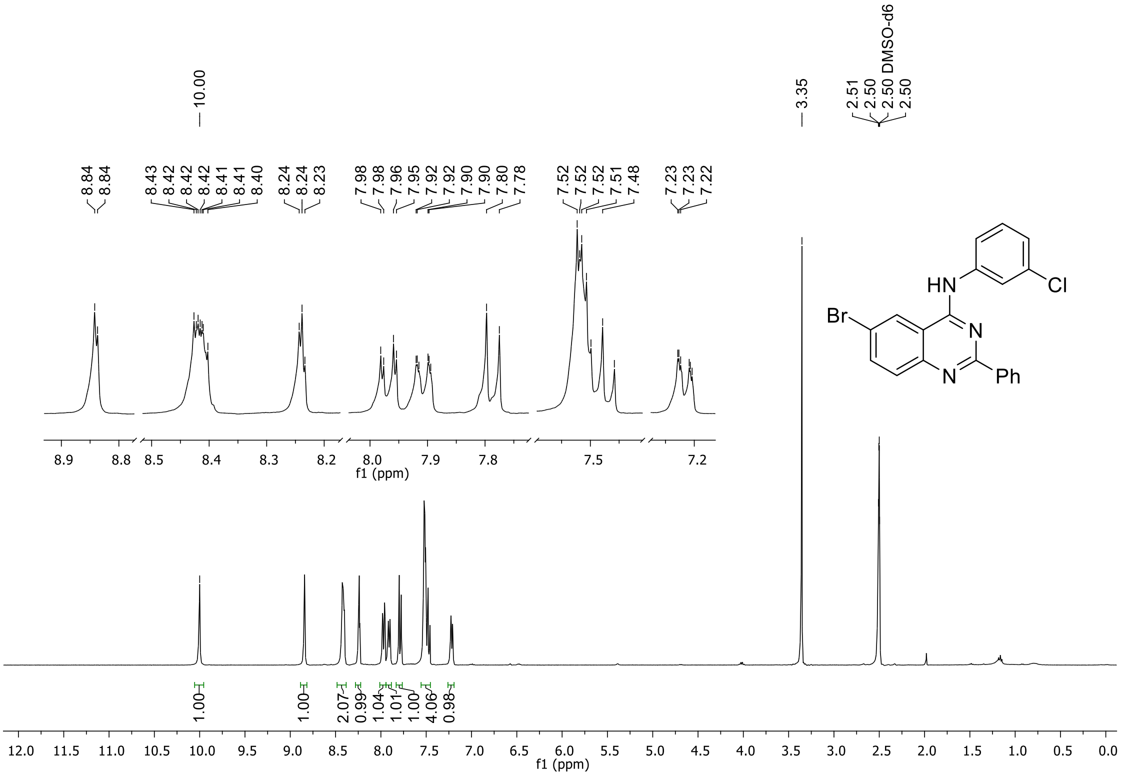

^{13}C NMR (100 MHz, DMSO-d6, ppm) of 6-bromo-*N*-(3-chlorophenyl)-2-phenylquinazolin-4-amine (**15e**)

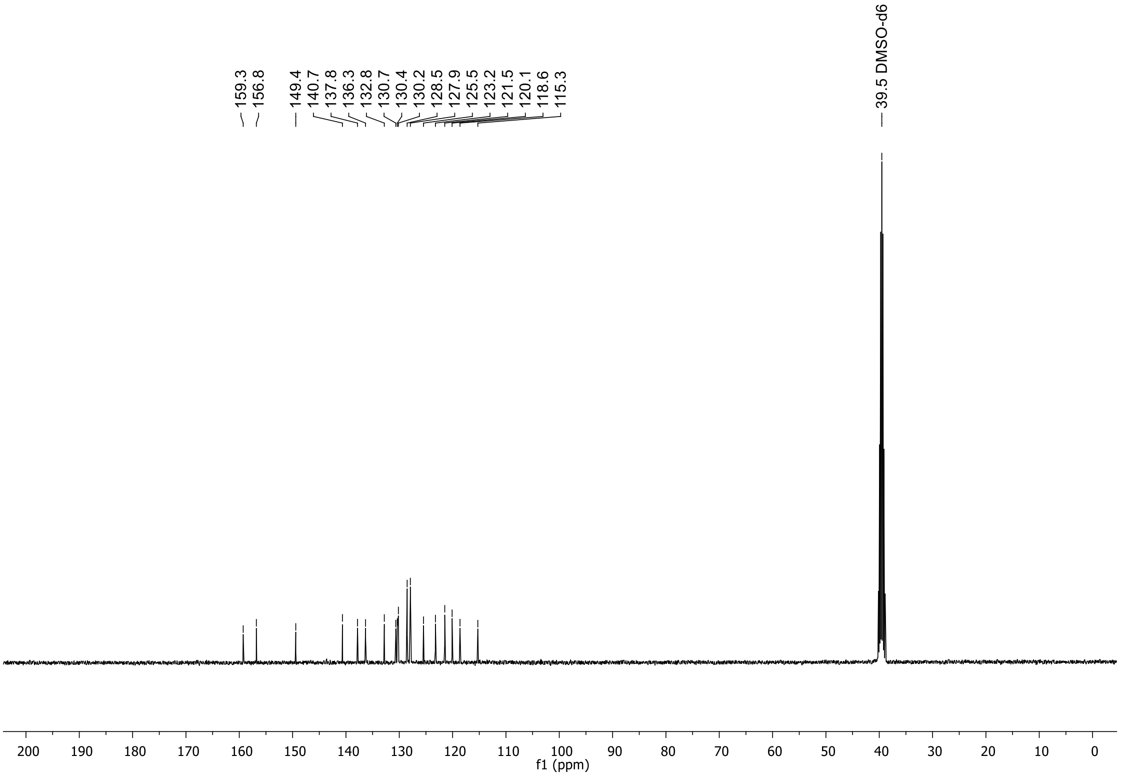

^{1}H NMR (400 MHz, DMSO-d6, ppm) of 6-bromo-*N*-(4-fluorophenyl)-2-phenylquinazolin-4-amine (**15f**)

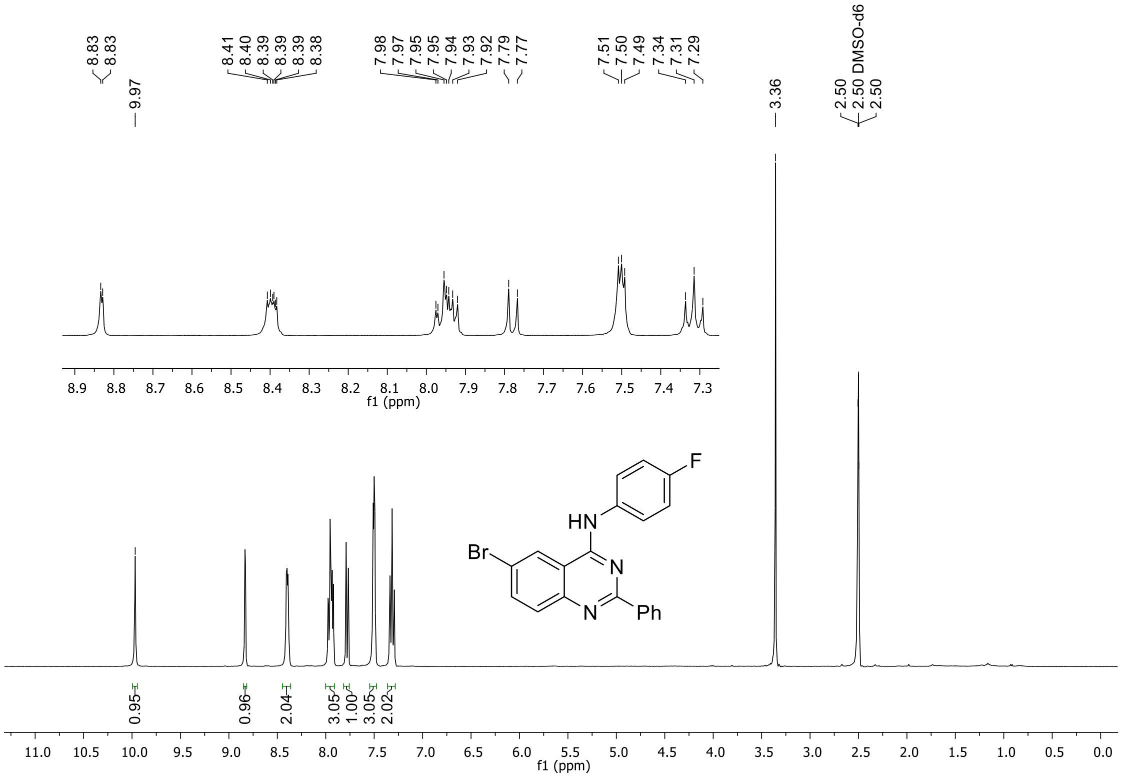

^{13}C NMR (100 MHz, DMSO-d6, ppm) of 6-bromo-*N*-(4-fluorophenyl)-2-phenylquinazolin-4-amine (**15f**)

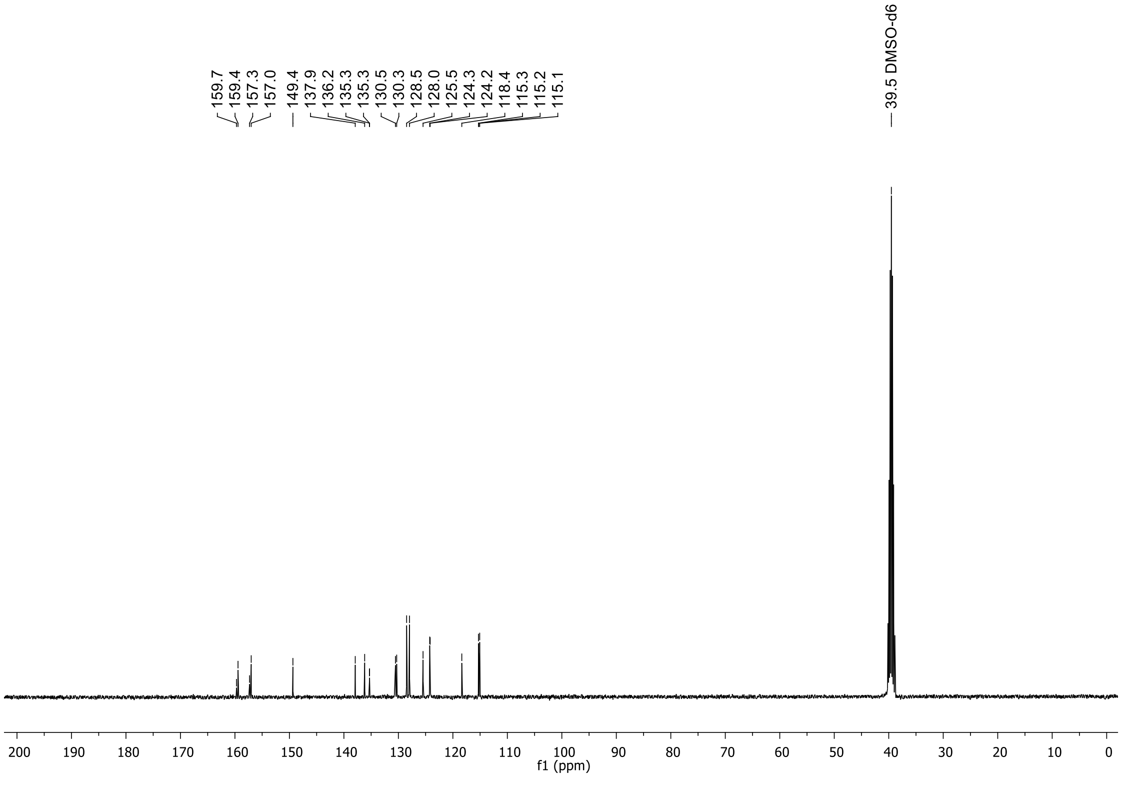

^{1}H NMR (400 MHz, $CDCl_3$, ppm) of 6-bromo-*N*-(4-methoxyphenyl)-2-phenylquinazolin-4-amine (**15g**)

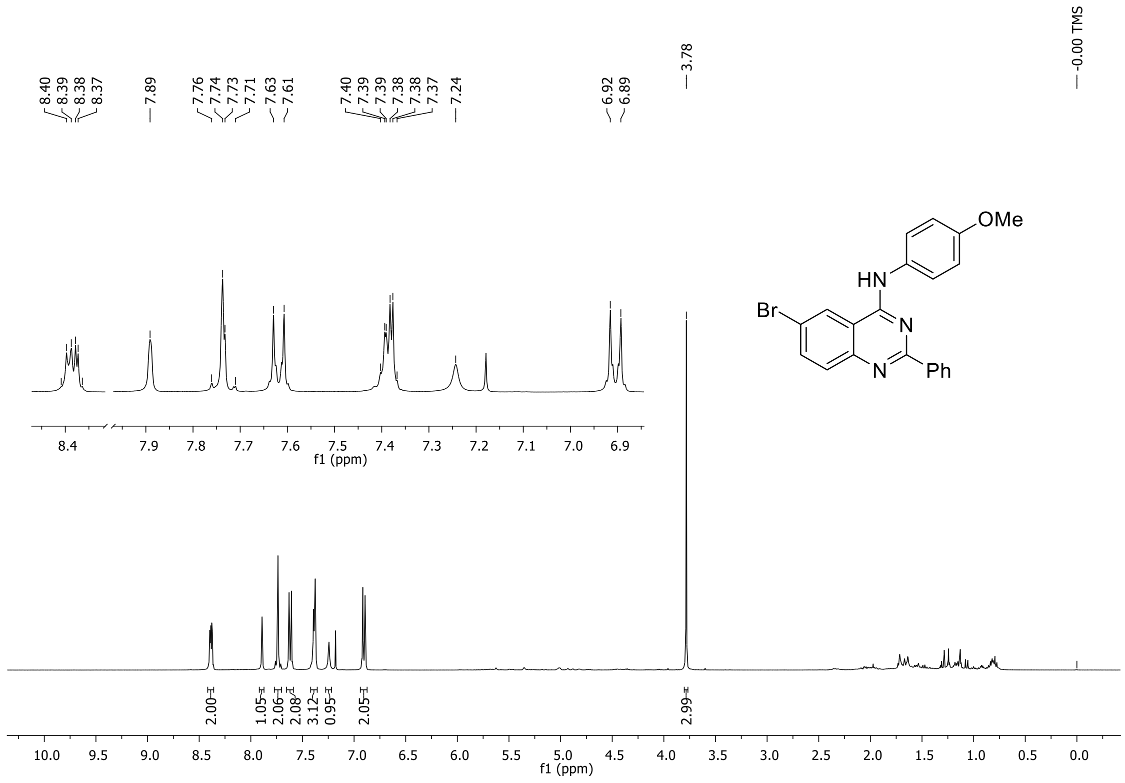

^{13}C NMR (100 MHz, $CDCl_3$, ppm) of 6-bromo-*N*-(4-methoxyphenyl)-2-phenylquinazolin-4-amine (**15g**)

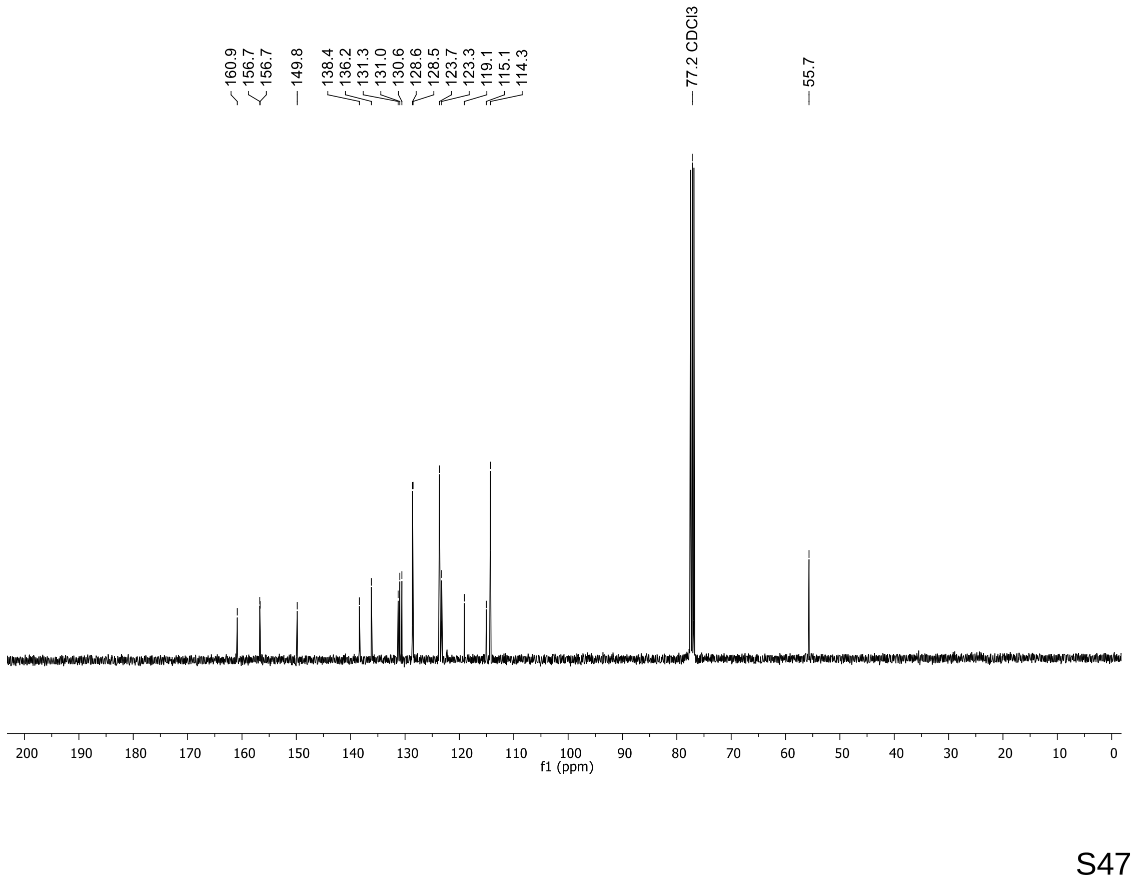

^{1}H NMR (400 MHz, $CDCl_3$, ppm) of 6-iodo-*N*-methyl-2-phenyl-*N*-(2-tolyl)quinazolin-4-amine (**10g**)

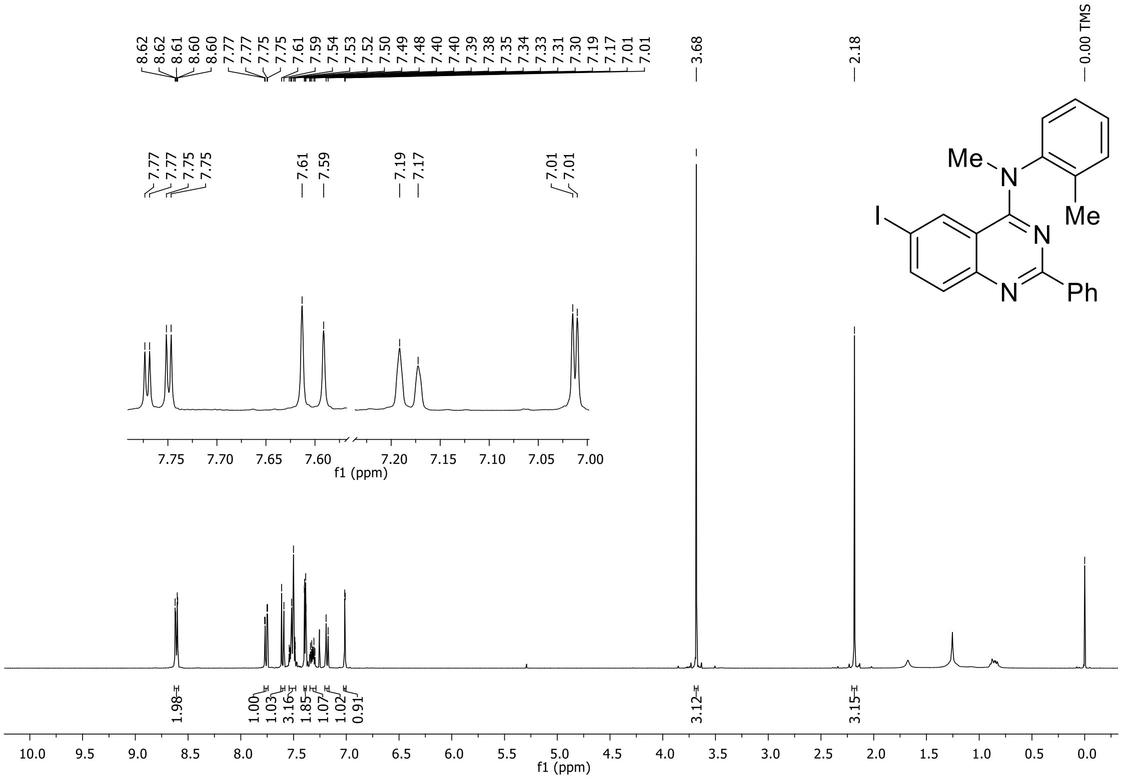

^{13}C NMR (100 MHz, $CDCl_3$, ppm) of 6-iodo-*N*-methyl-2-phenyl-*N*-(2-tolyl)quinazolin-4-amine (**10g**)

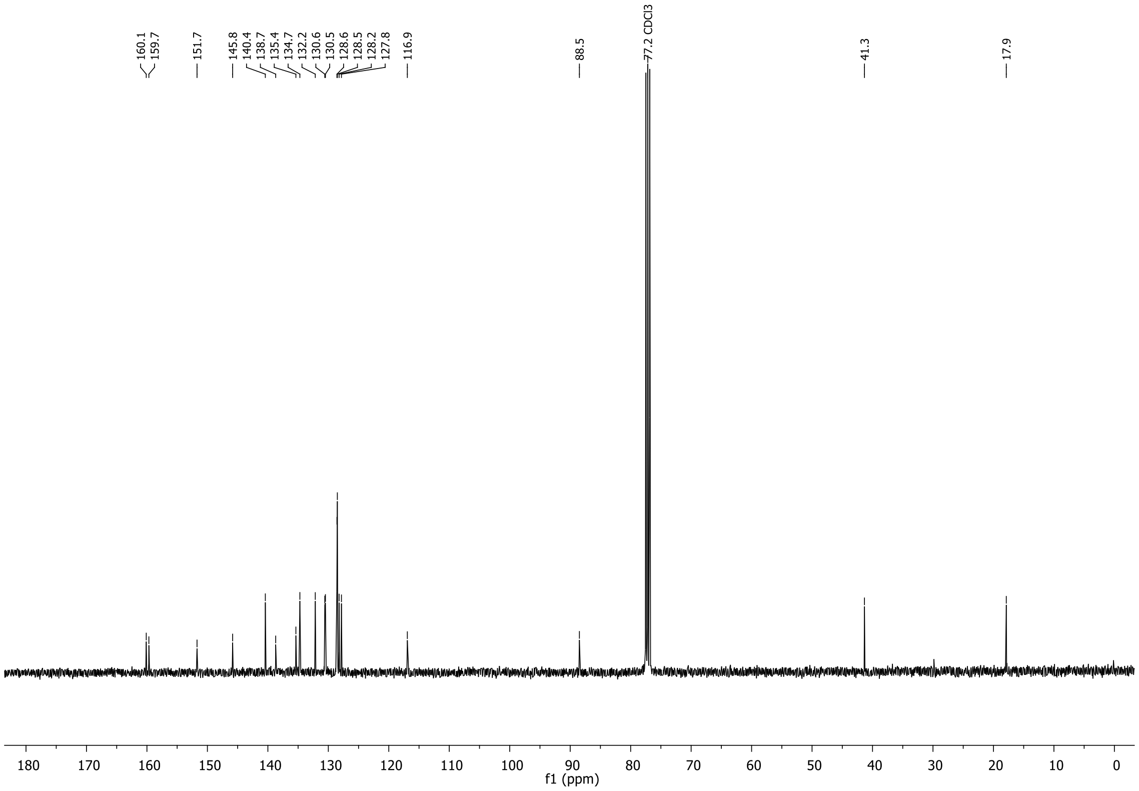

^{1}H NMR (400 MHz, $CDCl_3$, ppm) of 6-Bromo-*N*-methyl-2-phenyl-*N*-(2-tolyl)quinazolin-4-amine (**10h**)

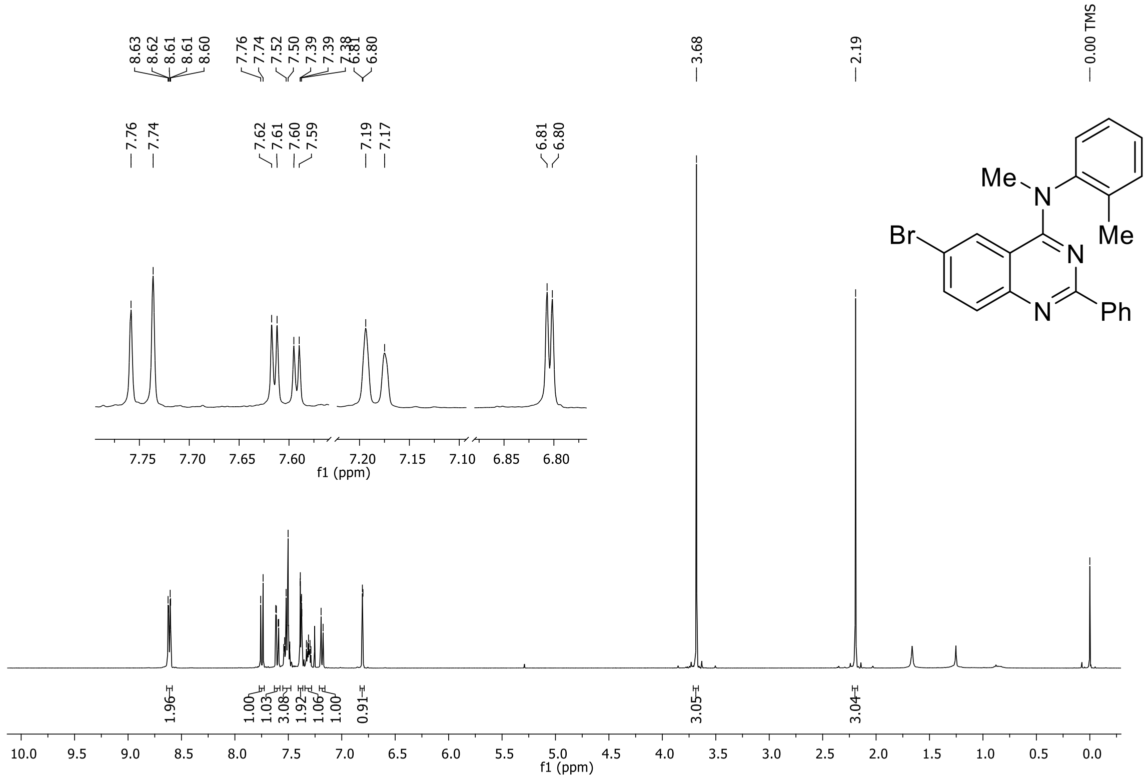

^{13}C NMR (100 MHz, $CDCl_3$, ppm) of 6-Bromo-*N*-methyl-2-phenyl-*N*-(2-tolyl)quinazolin-4-amine (**10h**)

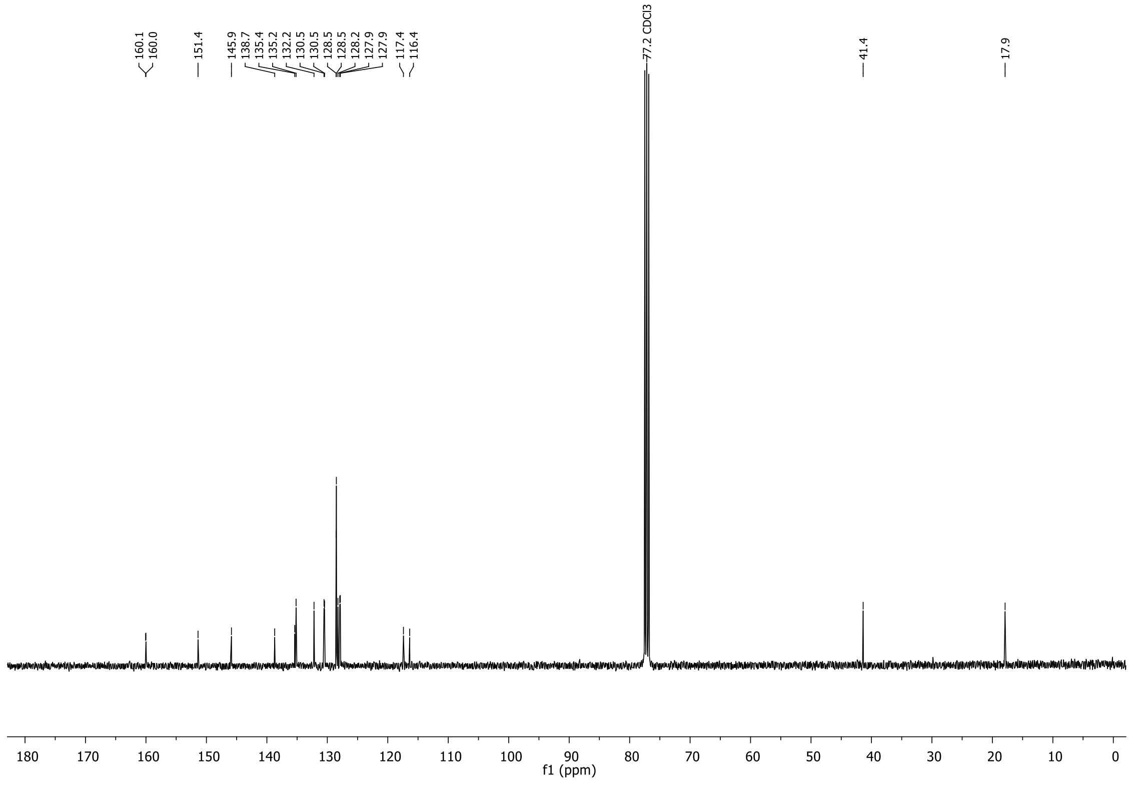

^{1}H NMR (400 MHz, $CDCl_3$, ppm) of *N*-(2-fluorophenyl)-6-iodo-*N*-methyl-2-phenylquinazolin-4-amine (**10q**)

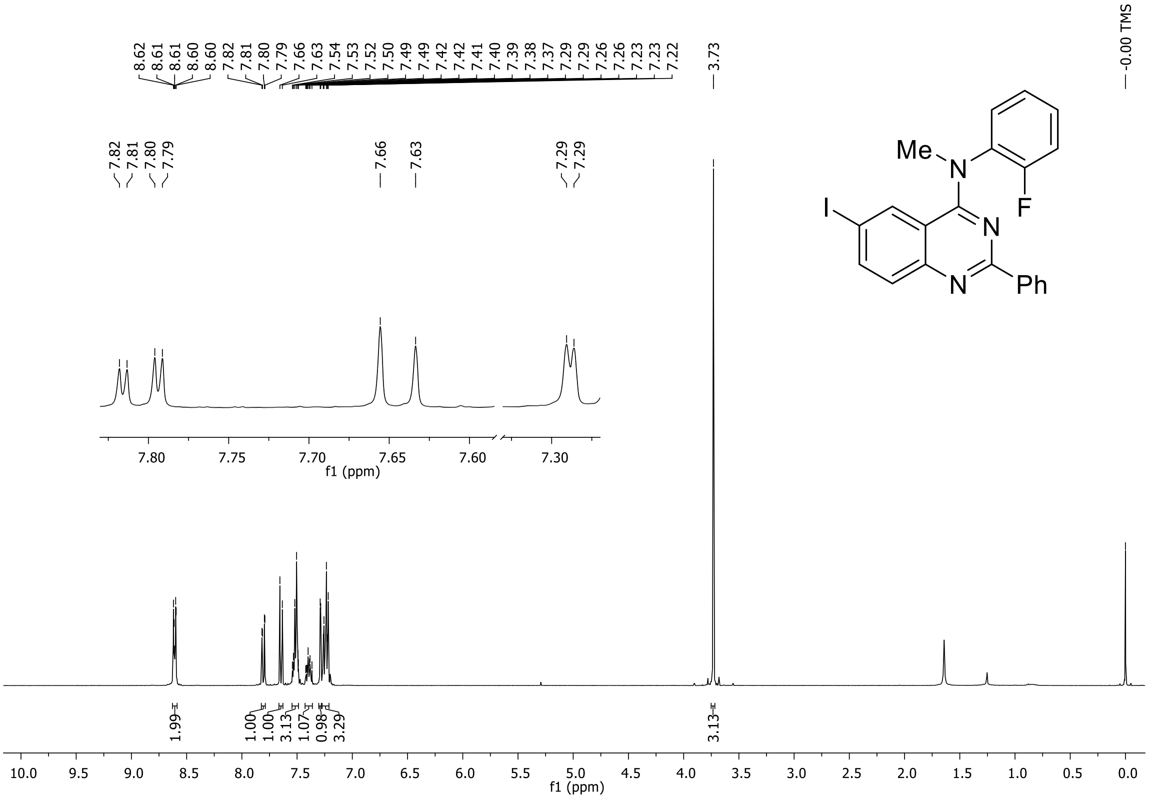

^{13}C NMR (100 MHz, $CDCl_3$, ppm) of *N*-(2-fluorophenyl)-6-iodo-*N*-methyl-2-phenylquinazolin-4-amine (**10q**)

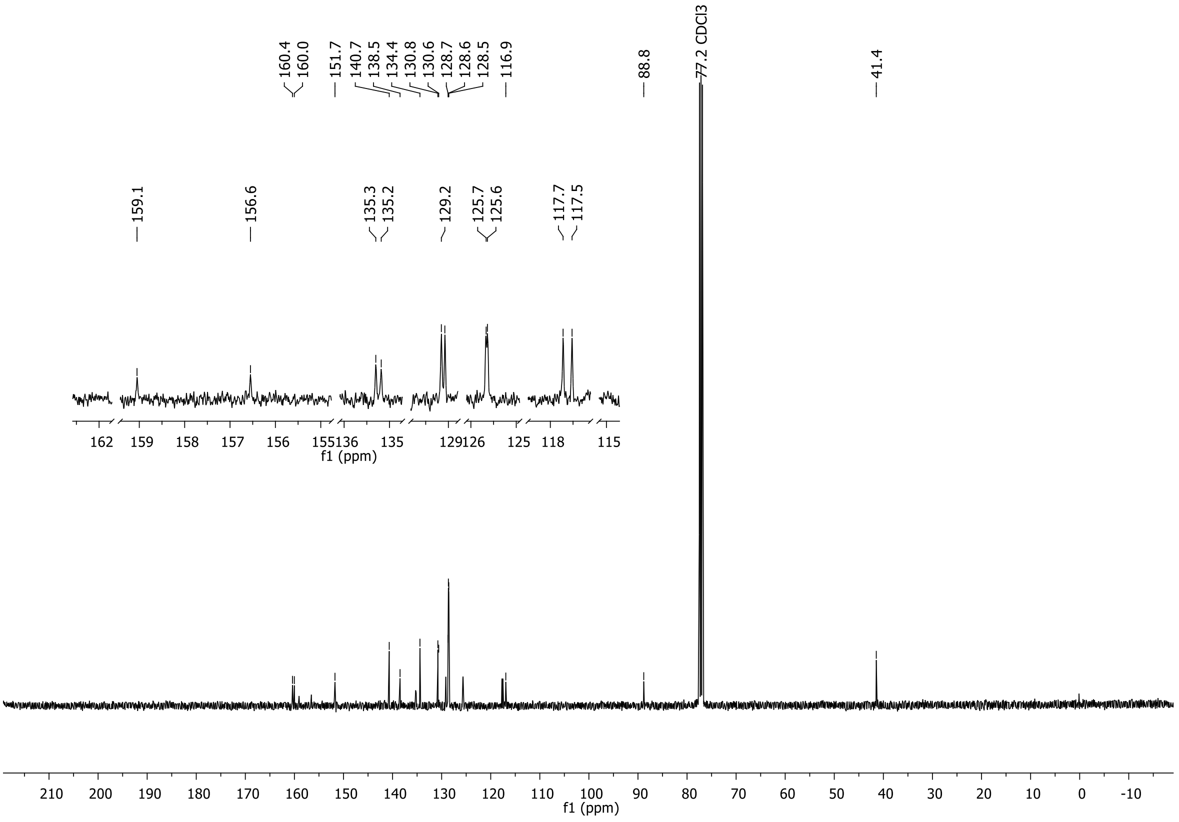

^{1}H NMR (400 MHz, $CDCl_3$, ppm) of 6-Bromo-*N*-(2-fluorophenyl)-*N*-methyl-2-phenylquinazolin-4-amine (**10r**)

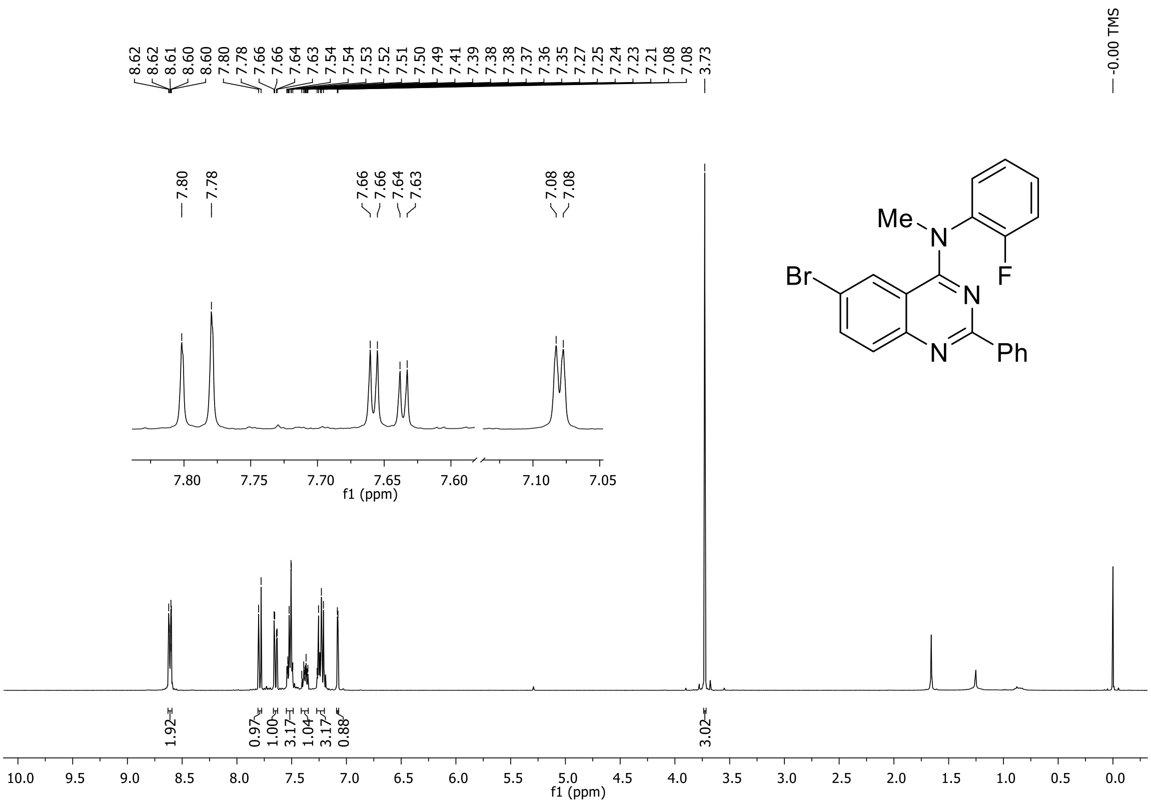

^{13}C NMR (100 MHz, $CDCl_3$, ppm) of 6-Bromo-*N*-(2-fluorophenyl)-*N*-methyl-2-phenylquinazolin-4-amine (**10r**)

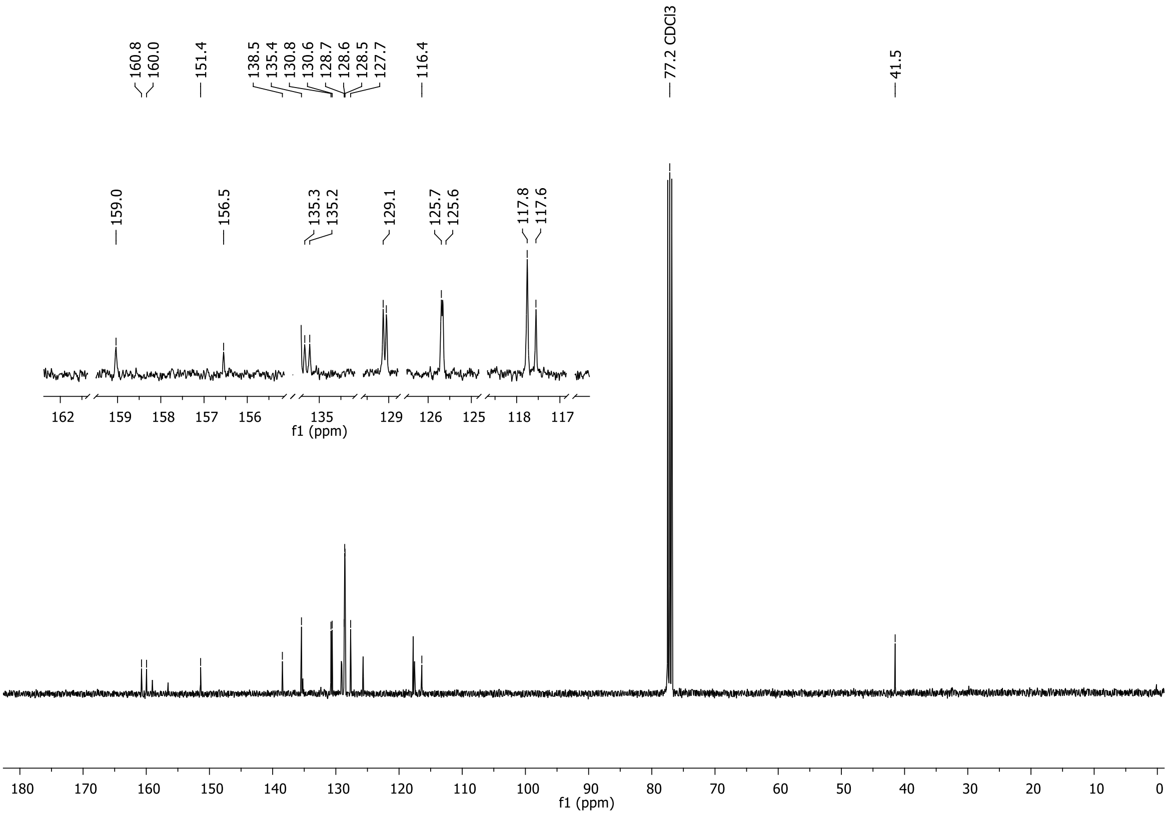

^{1}H NMR (400 MHz, $CDCl_3$, ppm) of 4-chloro-2-methylquinazoline (**17**)

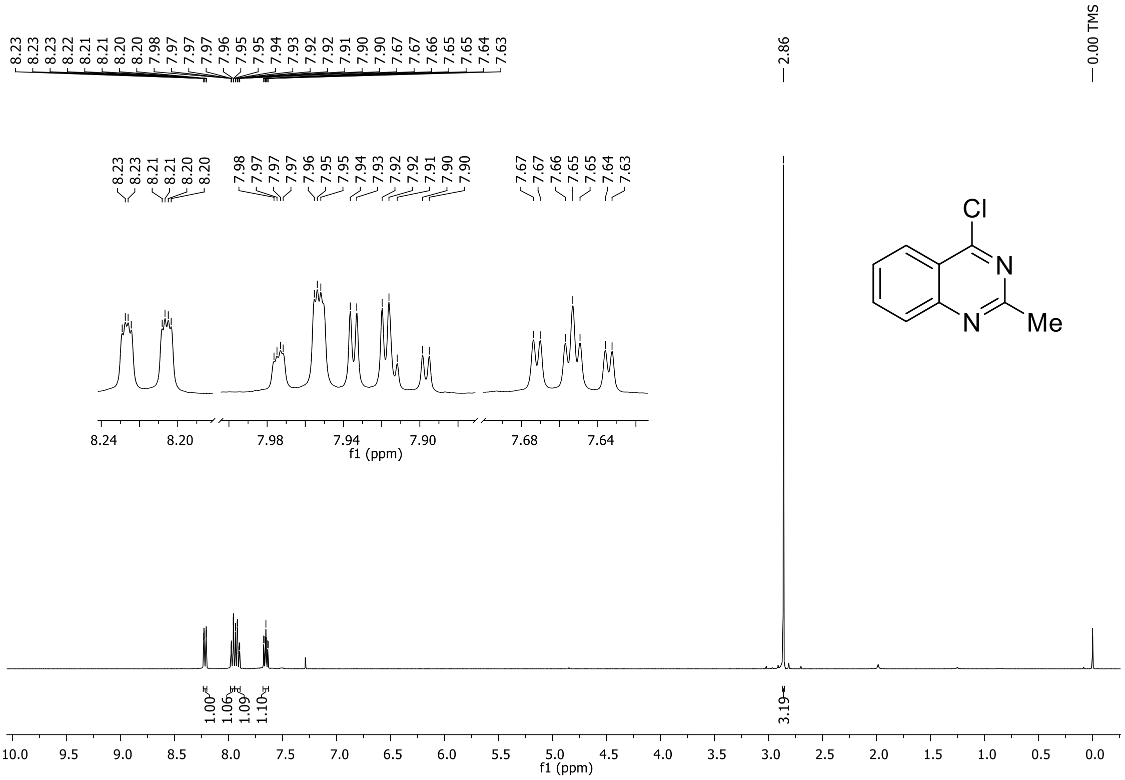

^{13}C NMR (100 MHz, $CDCl_3$, ppm) of 4-chloro-2-methylquinazoline (**17**)

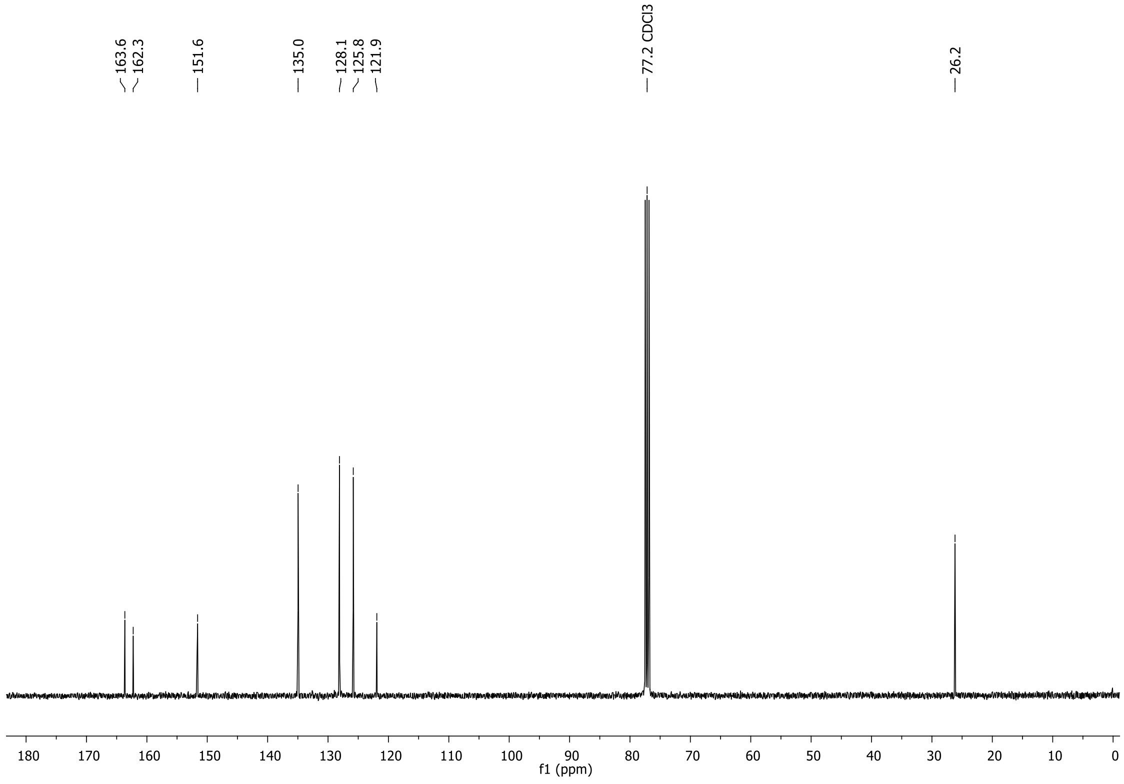

^{1}H NMR (400 MHz, $CDCl_3$, ppm) of *N*-(4-methoxyphenyl)-*N*,2-dimethyl-quinazolin-4-amine (Verubulin, **4**).

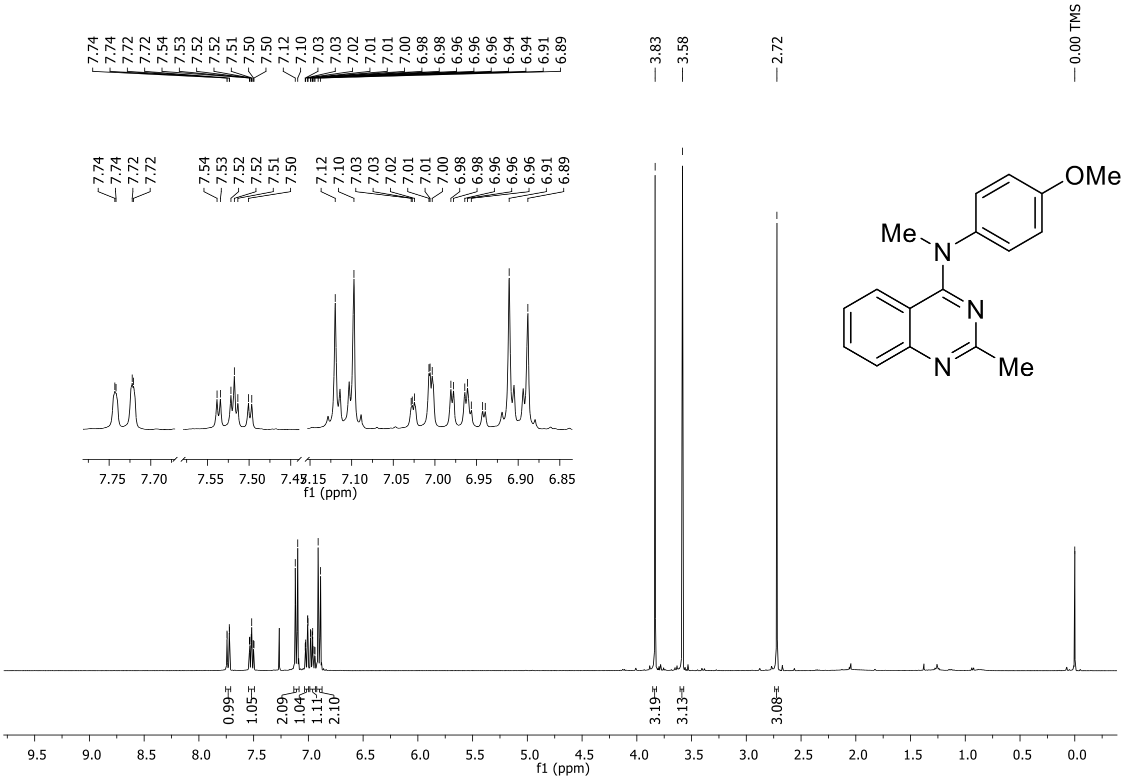

^{13}C NMR (100 MHz, $CDCl_3$, ppm) of *N*-(4-methoxyphenyl)-*N*,2-dimethyl-quinazolin-4-amine (Verubulin, **4**).

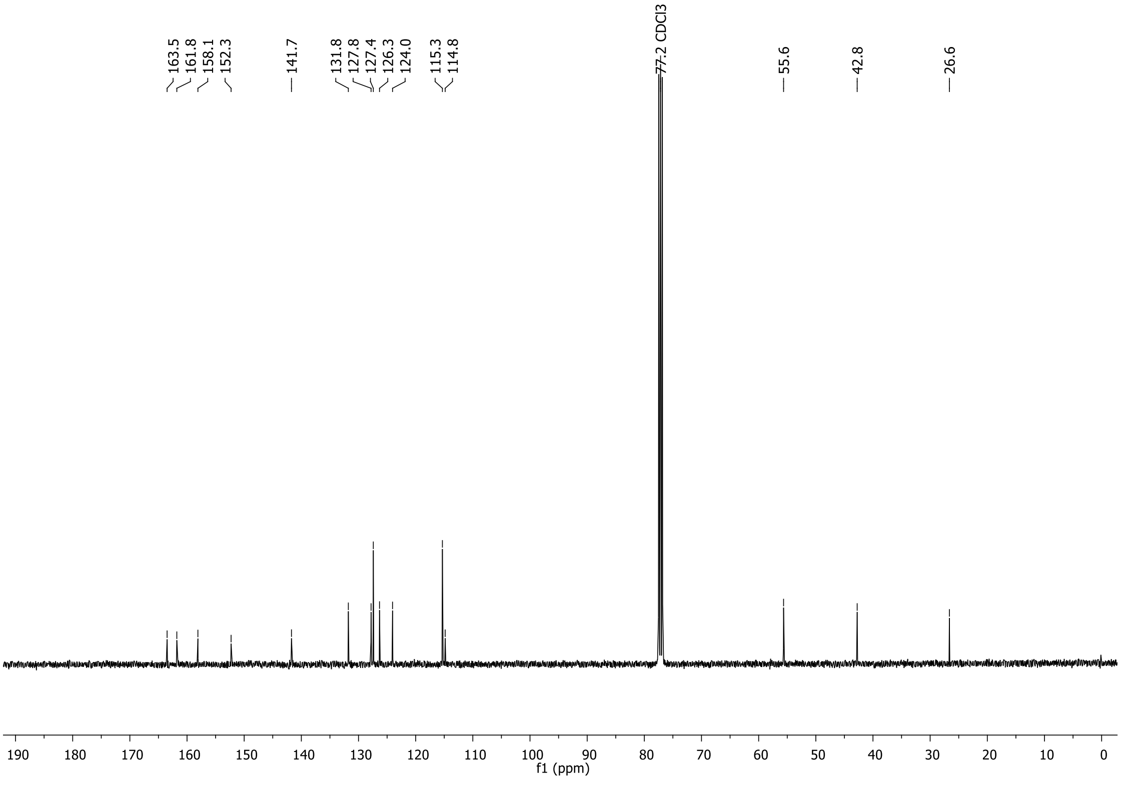

^{1}H NMR (400 MHz, $CDCl_3$, ppm) of *N*-methyl-*N*-phenylquinazolin-4-amine (**18**)

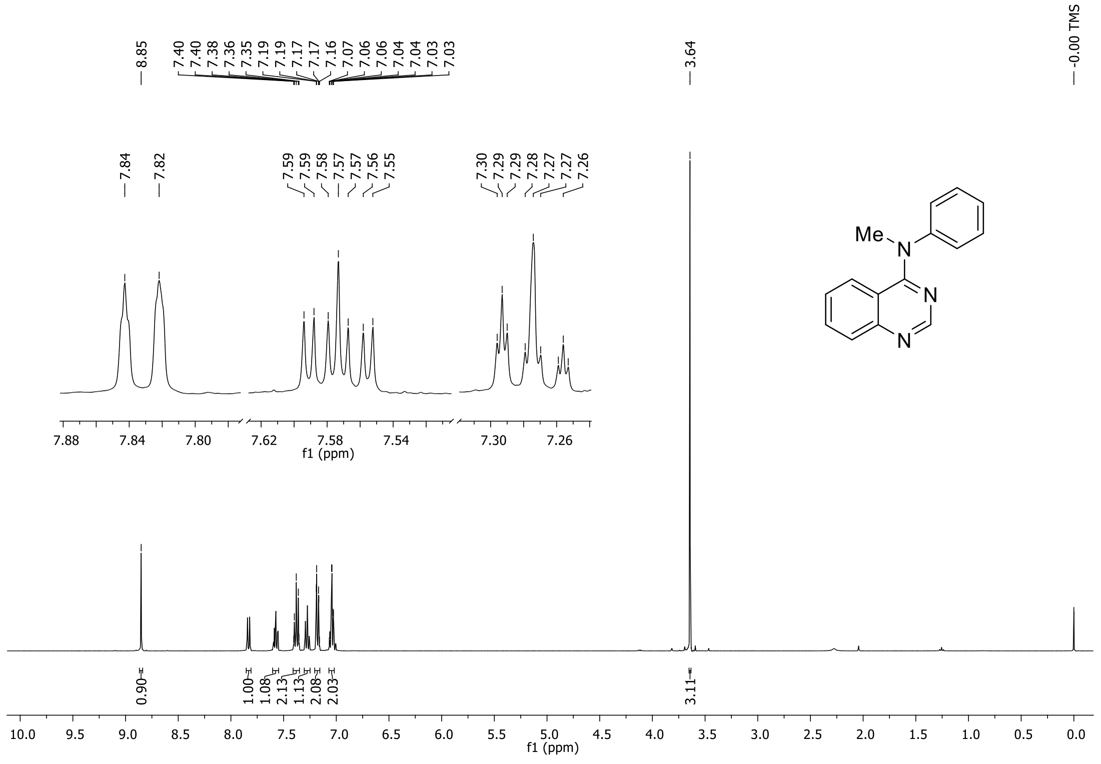

^{13}C NMR (100 MHz, $CDCl_3$, ppm) of *N*-methyl-*N*-phenylquinazolin-4-amine (**18**)

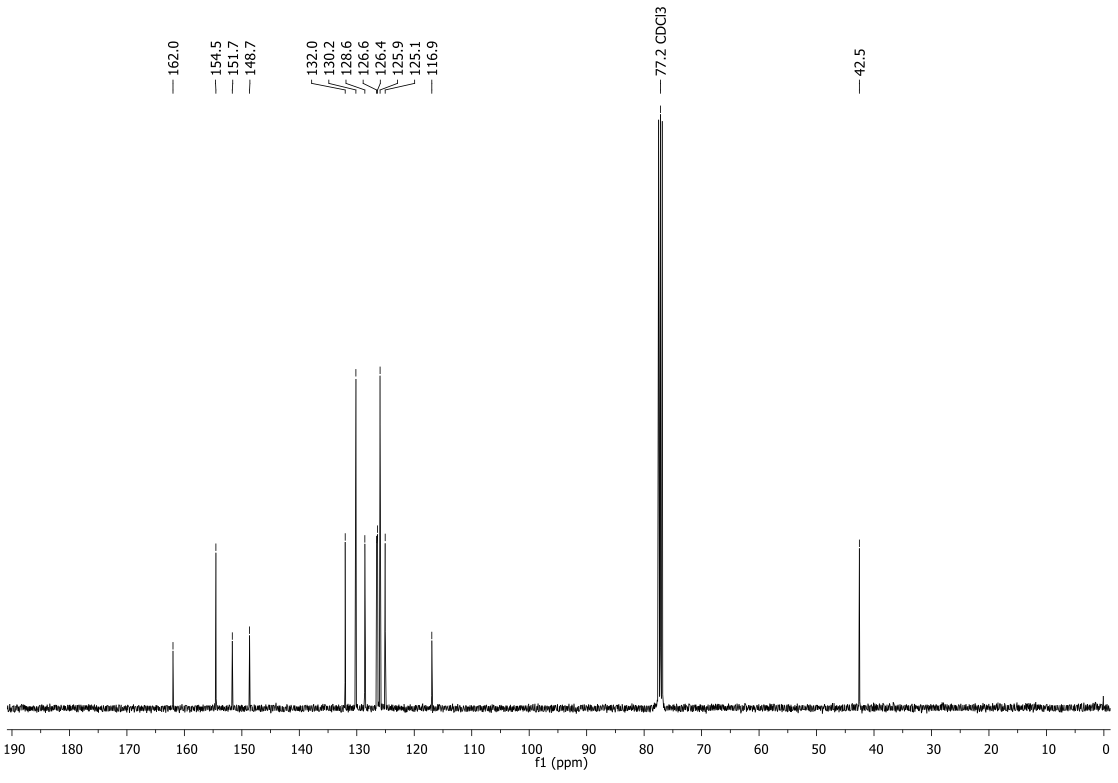

^{1}H NMR (400 MHz, $CDCl_3$, ppm) of *N*-(4-fluorophenyl)-*N*,2-dimethylquinazolin-4-amine (**19**)

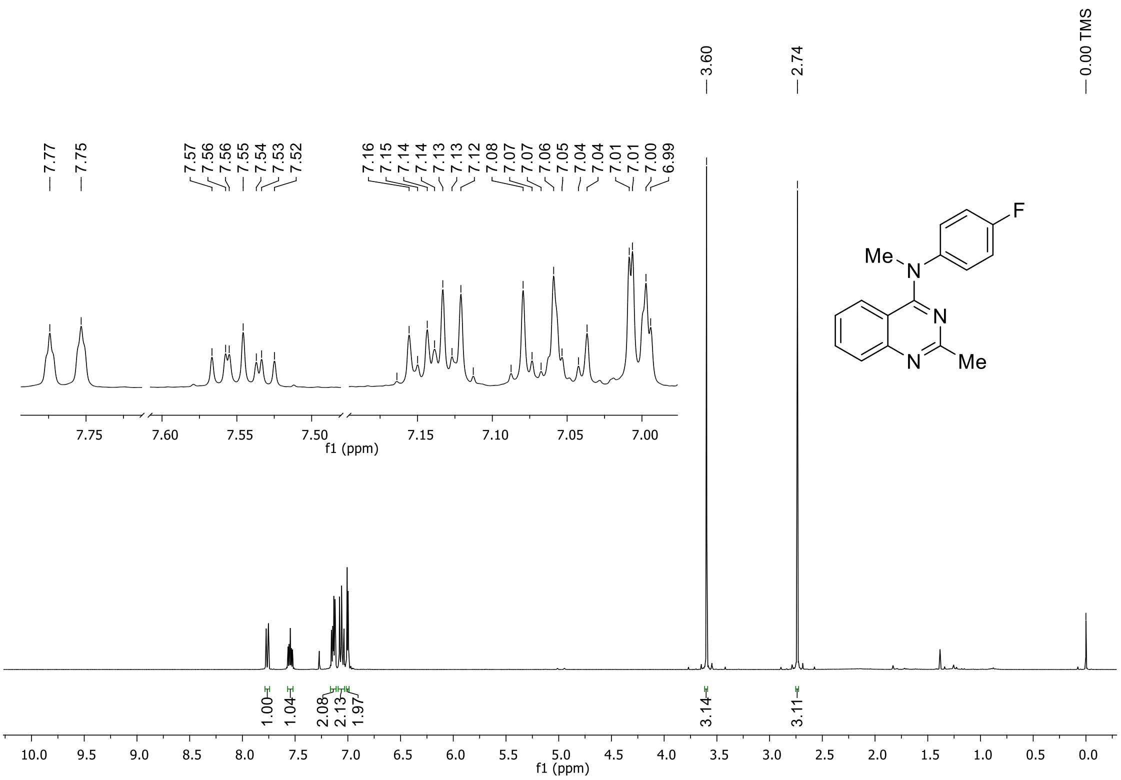

^{13}C NMR (100 MHz, $CDCl_3$, ppm) of *N*-(4-fluorophenyl)-*N*,2-dimethylquinazolin-4-amine (**19**)

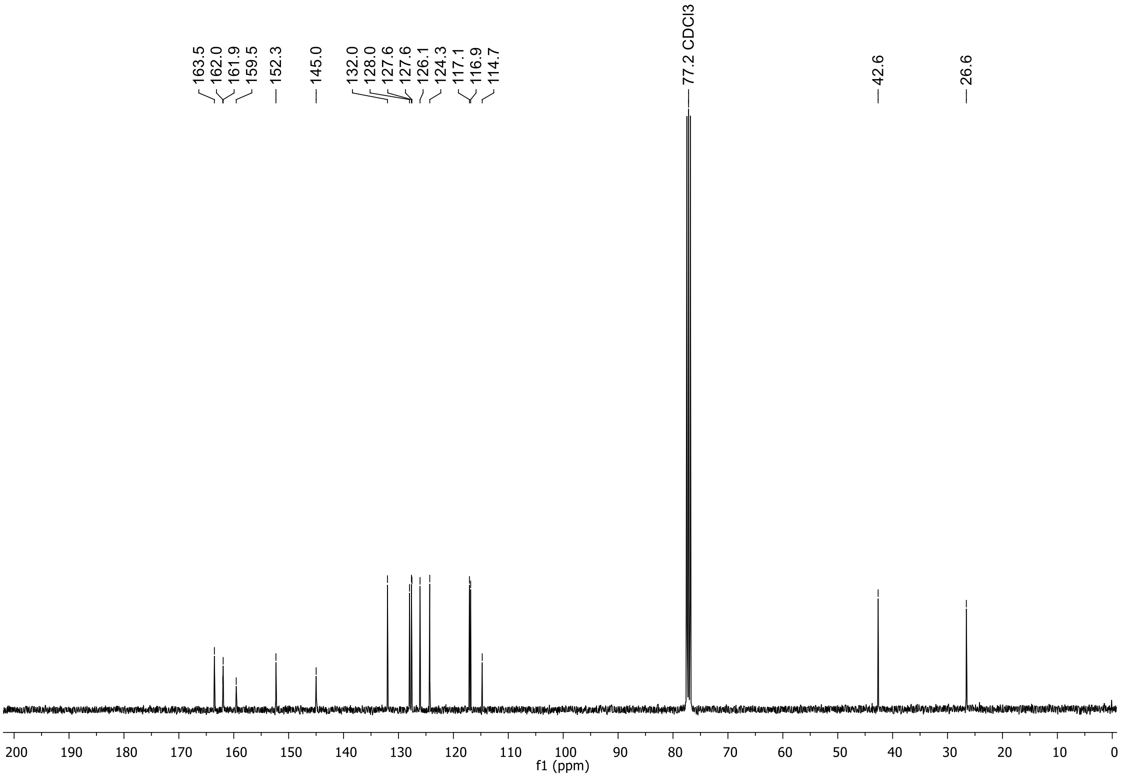

^{1}H NMR (400 MHz, $CDCl_3$, ppm) of *N*,2-dimethyl-*N*-phenylquinazolin-4-amine (**20**)

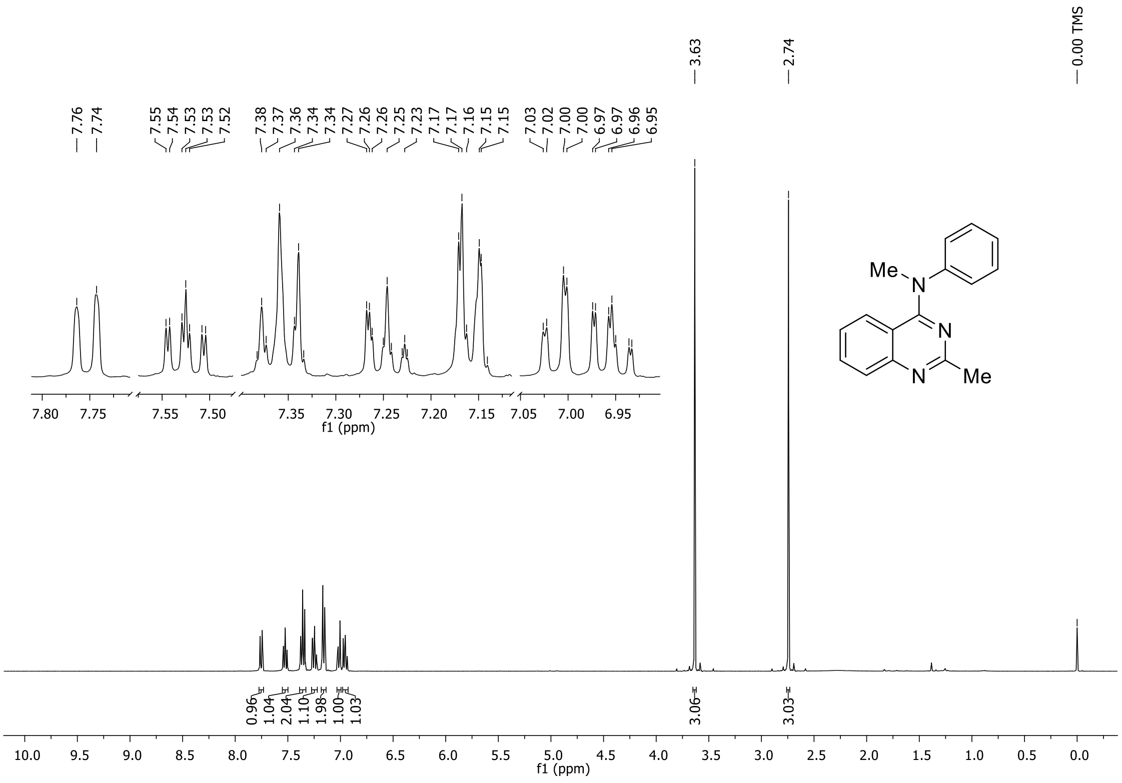

^{13}C NMR (100 MHz, $CDCl_3$, ppm) of *N*,2-dimethyl-*N*-phenylquinazolin-4-amine (**20**)

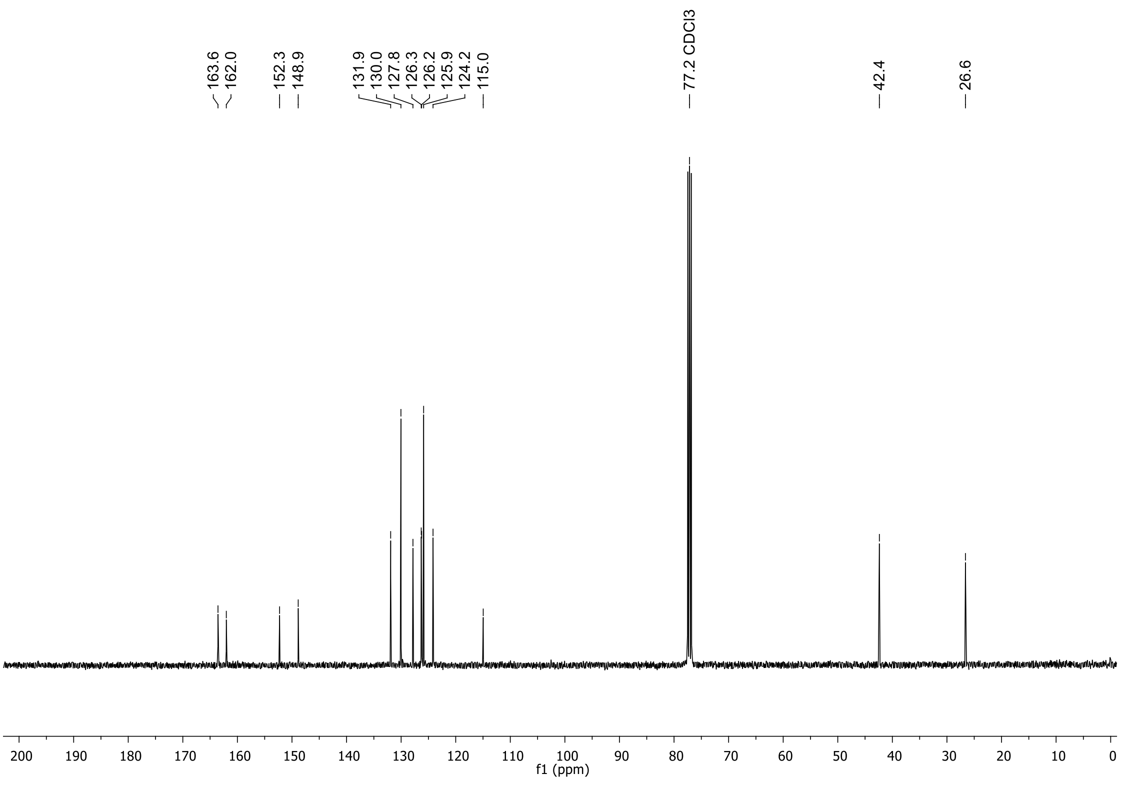

3. Biological assays

Antiproliferative assay

The antiproliferative activity of the synthesized compounds was initially screened against HCT-116 (human colorectal carcinoma), MCF-7 (human breast adenocarcinoma), and T98G (human glioblastoma) cell lines. The ability to inhibit at least 75% of cell growth evaluated through the colorimetric MTT (3-(4,5-dimethylthiazol-2-yl)-2,5-diphenyltetrazolium bromide) assay[1] was used to identify active compounds. A 10 mM stock solution in DMSO was prepared for each compound before the tests. DMSO and doxorubicin were used as negative and positive control, respectively. The cancer cells were seeded into a 96-well microplate at a density of 0.6×10^4 cells/well for HCT-116, 1×10^4 cells/well for MCF-7, and 0.2×10^4 cells/well for T98G and maintained for 24 h. After, cells were treated with the 4-anilinoquinazolines (hydrochloride salts) at the concentrations of 50 µM for initial tests. Inhibition curves were obtained using increasing concentrations from 0.0032 to 50 µM, in a dilution factor of five-fold for the novel 4-anilinoquinazolines, erlotinib hydrochloride, and gefitinib. For verubulin, we used concentrations ranging from 0.0032 to 500 nM. After the application of compounds, cells were incubated for 72 h. The supernatant was substituted by culture medium (150 µL/well) containing MTT (0.5 mg/mL) and the cells were incubated for an additional time, 3 h. Then, the supernatant was removed and the microplate was dried during at least 1 h. The precipitated formazan was dissolved in DMSO (150 µL/well) and the absorbance intensity was measured at 570 nm using a multi-well scanning spectrophotometer (Multiskan FC, Fisher Scientific, USA) [5]. Two or more experiments were carried out in duplicate.

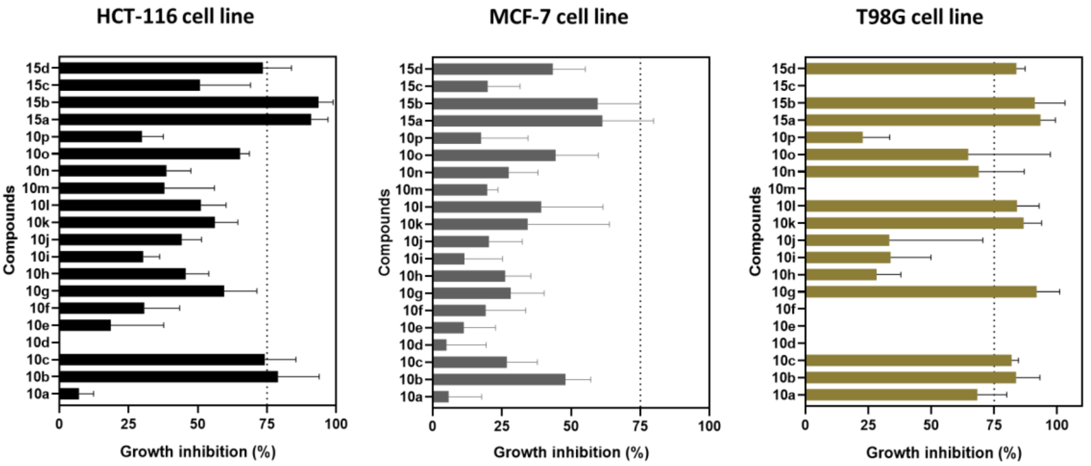

Figure S1: Percentage of inhibition of cell proliferation by novel 4-anilinoquinazolines in HCT-116, MCF-7 and T98G cell lines. Compounds were evaluated at 50 µM, and identified as active by inhibition of 75% of cell proliferation. Some 4-anilinoquinazolines not presented cell growth inhibition in HCT-116 and T98G. Two or more experiments were performed (n ≥ 2).

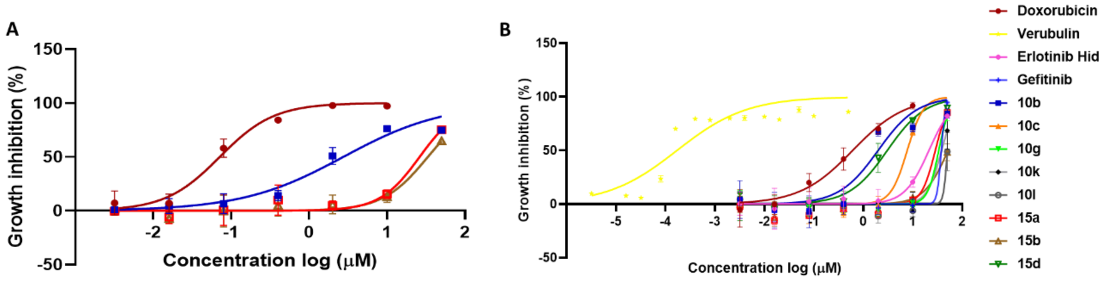

Figure S2: Dose-response curves of MTT assays to determine the IC_{50} values of 4-anilinoquinazolines on growth inhibition at 72 h of exposition. (A) Dose-response curves of 4-anilinoquinazolines in HCT-116 cells. (B) Dose-response curves of 4-anilinoquinazolines, erlotinib hydrochloride, gefitinib, and verubulin in T98G cells. Doxorubicin was used as positive control. Analyses were performed by GraphPad Prism 8.0 software using data from 2 independent experiments.

4. References

[1] Paumo, H. K.; Mphahlele, M. J.; Rhyman, L.; Ramasami, P. *Tetrahedron* **2016**, *72*, 123–133.

[2] Kijrungphaiboon, W.; Chantarasriwong, O.; Chavasiri, W. *Tetrahedron Lett.* **2012**, *53*, 674–677.

[3] Gallo, R. D. C.; Ferreira, I. M.; Casagrande, G. A.; Pizzuti, L.; Oliveira-Silva, D.; Raminelli, C. *Tetrahedron Lett.* **2012**, *53*, 5372–5375.

[4] Sirisoma, N.; Kasibhatla, S.; Pervin, A.; Zhang, H.; Jiang, S.; Willardsen, J. A.; Anderson, M. B.; Baichwal, V.; Mather, G. G.; Jessing, K.; Hussain, R.; Hoang, K.; Pleiman, C. M.; Tseng, B.; Drewe, J.; Cai, S. X. *J. Med. Chem.* **2008**, *51*, 4771–4779.

[5] Mosmann, T. *J. Immunol. Methods* **1983**, *65*, 55–63.
